# Alliance of Proteomics and Genomics to Unravel the Specificities of Sahara Bacterium *Deinococcus deserti*

**DOI:** 10.1371/journal.pgen.1000434

**Published:** 2009-03-27

**Authors:** Arjan de Groot, Rémi Dulermo, Philippe Ortet, Laurence Blanchard, Philippe Guérin, Bernard Fernandez, Benoit Vacherie, Carole Dossat, Edmond Jolivet, Patricia Siguier, Michael Chandler, Mohamed Barakat, Alain Dedieu, Valérie Barbe, Thierry Heulin, Suzanne Sommer, Wafa Achouak, Jean Armengaud

**Affiliations:** 1 CEA, DSV, IBEB, Laboratory of Microbial Ecology of the Rhizosphere and Extreme Environments (LEMiRE), Saint-Paul-lez-Durance, France; 2 CNRS, UMR 6191, Biologie Vegetale et Microbiologie Environnementales, Saint-Paul-lez-Durance, France; 3 Aix-Marseille Université, Saint-Paul-lez-Durance, France; 4 CEA, DSV, IBEB, Lab Biochim System Perturb, Bagnols-sur-Cèze, France; 5 CEA, Institut de Génomique, Génoscope, Evry Cedex, France; 6 Université Paris-Sud 11, CNRS UMR 8621, CEA LRC42V, Institut de Génétique et Microbiologie, Université Paris Sud, Orsay Cedex, France; 7 Laboratoire de Microbiologie et Génétique Moléculaire, CNRS UMR5100, Toulouse Cedex, France; Progentech, United States of America

## Abstract

To better understand adaptation to harsh conditions encountered in hot arid deserts, we report the first complete genome sequence and proteome analysis of a bacterium, *Deinococcus deserti* VCD115, isolated from Sahara surface sand. Its genome consists of a 2.8-Mb chromosome and three large plasmids of 324 kb, 314 kb, and 396 kb. Accurate primary genome annotation of its 3,455 genes was guided by extensive proteome shotgun analysis. From the large corpus of MS/MS spectra recorded, 1,348 proteins were uncovered and semiquantified by spectral counting. Among the highly detected proteins are several orphans and *Deinococcus*-specific proteins of unknown function. The alliance of proteomics and genomics high-throughput techniques allowed identification of 15 unpredicted genes and, surprisingly, reversal of incorrectly predicted orientation of 11 genes. Reversal of orientation of two *Deinococcus*-specific radiation-induced genes, *ddrC* and *ddrH*, and identification in *D. deserti* of supplementary genes involved in manganese import extend our knowledge of the radiotolerance toolbox of *Deinococcaceae*. Additional genes involved in nutrient import and in DNA repair (i.e., two extra *recA*, three translesion DNA polymerases, a photolyase) were also identified and found to be expressed under standard growth conditions, and, for these DNA repair genes, after exposure of the cells to UV. The supplementary nutrient import and DNA repair genes are likely important for survival and adaptation of *D. deserti* to its nutrient-poor, dry, and UV-exposed extreme environment.

## Author Summary

*D. deserti* belongs to the *Deinococcaceae*, a family of bacteria characterized by an exceptional ability to withstand the lethal effects of DNA-damaging agents, including ionizing radiation, UV light, and desiccation. It was isolated from Sahara surface sands, an extreme and nutrient-poor environment, regularly exposed to intense UV radiation, cycles of extreme temperatures, and desiccation. The evolution of organisms that are able to survive acute irradiation doses of 15,000 Gy is difficult to explain given the apparent absence of highly radioactive habitats on Earth over geologic time. Thus, it seems more likely that the natural selection pressure for the evolution of radiation-resistant bacteria was chronic exposure to nonradioactive forms of DNA damage, in particular those promoted by desiccation. Here, we report the first complete genome sequence of a bacterium, *D. deserti* VCD115, isolated from hot, arid desert surface sand. Accurate genome annotation of its 3,455 genes was guided by extensive proteome analysis in which 1,348 proteins were uncovered after growth in standard conditions. Supplementary genes involved in manganese import, in nutrient import, and in DNA repair were identified and are likely important for survival and adaptation of *D. deserti* to its hostile environment.

## Introduction

The surface sands of hot arid deserts are exposed to intense ultraviolet (UV) radiation, cycles of extreme temperatures, and desiccation. Nevertheless, an extensive diversity of bacterial species has been identified in such extreme and nutrient-poor environments [1],[2]. To better understand how life is adapted to these specific environmental conditions, we are characterizing *Deinococcus deserti* strain VCD115, recently isolated from upper sand layers of the Sahara [3].

*D. deserti* belongs to the *Deinococcaceae*, a family of extremely radiation tolerant bacteria that comprises more than 30 species in a single genus. Deinococci have been isolated from a wide range of environments, such as soil, water, air, faeces, hot springs and irradiated food [4]. Fourteen of the currently recognized *Deinococcus* species were isolated from arid environments, like desert soil and antarctic rock. Among the Deinococci, *Deinococcus radiodurans* is by far the best characterized and was first isolated more than 50 years ago from canned meat that had been exposed to a high dose of ionizing radiation [5]. Its genome sequence was published in 1999 [6]. More recently, the genome sequence of the slightly thermophilic *Deinococcus geothermalis*, isolated from a hot spring in Italy [7], was determined [8].

Heavy UV- and desiccation-induced damage to membranes, proteins and nucleic acids is lethal to most organisms. Vegetative bacteria that survive these stresses must therefore either protect vital components from damage and/or repair them efficiently, especially upon rehydration [9]. For *D. radiodurans*, it was suggested that its tolerance to high doses of ionizing radiation is a consequence of its response to natural DNA damaging conditions such as desiccation [10],[11]. Its extreme resistance phenotype has not been fully explained, but has been proposed to result from a combination of different molecular mechanisms and physiological determinants (for reviews, see [4],[12]). Repair of massive DNA damage in *D. radiodurans* involves widespread DNA repair proteins, such as RecA and PolA [13]. In addition, the use of microarrays resulted in the identification of various hypothetical genes highly induced following gamma irradiation or desiccation [11]. Of these, *ddrA*, *ddrB*, *ddrC*, *ddrD* and *pprA* genes were shown to be involved in DNA repair or radiation tolerance [11], and the proteins DdrA (DNA extremity protection) [14] and PprA (stimulation of ligase activity) [15] were characterized *in vitro*. Tolerance to desiccation and radiation does not only imply DNA repair but also protection of proteins from oxidative damage by a high intracellular Mn/Fe concentration ratio [16]. Moreover, *D. radiodurans* encodes plant protein homologs involved more exclusively in its tolerance to desiccation [17].

*D. radiodurans* is one of the first bacteria whose genomes were completely sequenced [6],[18]. Since this pioneering work, more than 600 bacterial genomes have been sequenced. However, very few of these organisms have been thoroughly analyzed at both the genome and proteome levels. Over recent years, various proteomic strategies based on liquid chromatography-coupled tandem mass spectrometry (LC-MS/MS) technology were developed as an aid to genomic annotation [19]. Validation of the existence of orphan genes, prediction of short genes, annotation of genes with unusual codon usage, accurate determination of start codons, and post-translational modifications can be deduced through detection and sequencing of peptides [20],[21]. Such proteogenomic strategies were initially used to re-analyse previously annotated and published genomes such as the relatively small bacterial genome of *Mycoplasma pneumoniae* [22], and then larger genomes such as that of *Shewanella oneidensis* [23]. They were further applied to the primary annotation of a newly sequenced but related bacterium, *Mycoplasma mobile* [24]. Transcriptomics may also be helpful to validate gene predictions, and may lead to gene discovery when tiling arrays that span the entire genome are used [25].

To learn more about the mechanisms of adaptation of *D. deserti* to the harsh environmental conditions found in the Sahara desert, we determined and accurately annotated its entire 3.86 Mb genome sequence in parallel with an extensive proteome analysis. After the 777 kb genome of *M. mobile*, this is the second bacterial genome whose primary annotation was guided by proteomics. We developed a new strategy that allowed 40% coverage of the whole theoretical proteome for a standard cultivation condition only. In addition, the proteome analysis allowed correction of unexpected gene prediction errors. Comparative genomics combined with proteomics data revealed novel *Deinococcus*-specific proteins that could be involved in stress tolerance mechanisms, as well as specific characteristics of *D. deserti* VCD115 that may contribute to survival and adaptation of this unique bacterium to dry desert soil.

## Results

### Resistance of *D. deserti* to desiccation, UV and gamma radiation

**Figure 1:**
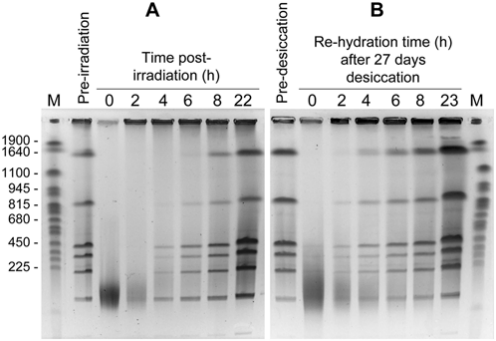
Kinetics of genome reconstitution in *D. deserti* after gamma radiation and after desiccation. *D. deserti* cells were subjected to 6.8 kGy gamma rays (Panel A) or 27 days of dessication (Panel B). Genomic DNA, purified from cells before irradiation or desiccation and at different times after irradiation or rehydration, was digested with PmeI and SwaI, resulting in 8 DNA fragments for an intact genome, and separated by pulsed-field gel electrophoresis (PFGE). Lanes M, Yeast Chromosome PFG Marker (New England Biolabs). Lengths (in kb) of several marker fragments are indicated on the left.

Besides its resistance to high doses of gamma and UV radiation [3], *D. deserti* also tolerated prolonged desiccation, with about 50% survival after 40 days of desiccation. Genome repair of *D. radiodurans* has been analyzed with cells grown and recovered in rich medium. As *D. deserti* is unable to grow in rich media, the fate of its DNA after exposure to a high dose of gamma radiation (6.8 kGy) or after 27 days of desiccation was analyzed with cells pre-grown and recovered in tenfold diluted tryptic soy broth (TSB/10). Like gamma-irradiation, desiccation generated numerous double-strand DNA breaks in *D. deserti*. An intact genome was reconstituted within six to eight hours under these conditions (Figure 1). Therefore, *D. deserti*'s tolerance to desiccation is related to efficient DNA repair as found in *D. radiodurans* [10] rather than DNA protection mechanisms as observed in *Nostoc commune* [26].

### Genome sequence and structure: general features

The genome of *D. deserti* VCD115 is composed of four replicons: a main chromosome (2.82 Mb) and three plasmids, P1 (325 kb), P2 (314 kb) and P3 (396 kb) (Genbank accession numbers CP001114, CP001115, CP001116 and CP001117, respectively). Table 1 presents their main characteristics. Counting of sequence reads gave the relative abundance of each replicon: 1∶1∶1∶1 (±2%). Genome size and equimolarity of the four DNA entities were consistent with PFGE results (data not shown). The main chromosome contains three 16S rRNA genes at different locations, and three clusters located elsewhere that each contain one 23S and one 5S rRNA gene. A single cluster containing one 16S, one 23S and one 5S rRNA gene is also present on plasmid P1. The rRNA genes on P1 are identical to the corresponding genes on the chromosome.

**Table 1:** General characteristics of the *D. deserti* genome.

Molecule	Chromosome	Plasmid P1	Plasmid P2	Plasmid P3	All
**General characteristics**					
Size (bp)	2,819,842	324,711	314,317	396,459	3,855,329
GC content (%)	63.38	60.7	63.53	61.41	62.96
Coding density (%)	85.53	72.53	83.74	78.56	84.31
rRNAs	9	3	—	—	12
tRNAs	48	—	—	—	48
miscRNAs^a^	5	—	—	—	5
Repeat content (%)	<1	<1	<1	<1	<1
IS content^b^ (%)	0.3	2.05	1.46	1.97	0.71
**Proteins**					
Protein-coding genes	2,594	262	250	349	3455
Detected by MS	1,155	44	83	66	1,348
With assigned function	1,785	160	191	249	2,385
Detected by MS	918	34	65	53	1,070
Conserved hypothetical	611	32	32	45	720
Detected by MS	210	4	11	9	234
Hypothetical	198	70	27	55	350
Detected by MS	27	6	7	4	44

a Other non-coding RNAs: one RNase P RNA, one SRP RNA, one tmRNA, two THI elements (TPP riboswitch).

b Complete and partial IS elements.

Nine complete and different insertion sequences (IS), designated IS*Dds1* to IS*Dds9* according to the standard IS nomenclature with a total of 13 copies and belonging to 6 distinct IS families, were identified in *D. deserti* (Table S1; see also www-is.biotoul.fr). One of these, IS*Dds1* (an IS*3* family member), is present in 5 copies, whereas each of the remaining 8 ISs is present in only one copy. Remarkably, *D. deserti* contains many fewer IS elements than *D. radiodurans* and *D. geothermalis* (Table S1): 13, 46 and 72, respectively. The IS family composition also differs in these species; for example, complete IS*200*/IS*605* elements are absent in *D. deserti* but present in the other two Deinococci, while complete IS*3* elements, present in *D. deserti*, are absent in *D. radiodurans* and *D. geothermalis*.

### Proteome fractionation and extra-large MS/MS shotgun analysis

We investigated several strategies based on 1D SDS-PAGE and shotgun nanoLC-MS/MS to fractionate the *D. deserti* proteome from cells collected either in exponential phase or stationary phase: longer migration, increase of the percentage of acrylamide for covering low molecular weight proteins, additional separation by chromatography prior to SDS-PAGE. Ammonium sulphate precipitation followed by phenyl sepharose chromatography was a good means to considerably expand the proteome coverage, as measured by the number of proteins detected and their peptide coverage. Small coding sequences (CDSs) for non-conserved proteins are difficult to predict. Moreover, many small prokaryotic proteins are likely to show low gene expression levels as suggested by their low average codon adaptation index [27]. These low molecular weight proteins (below 50 kDa) represent two thirds of the theoretical proteome. We therefore identified these systematically using appropriate SDS-PAGE conditions. After 369 nanoLC-MS/MS runs on ESI-ion trap mass spectrometers, a large corpus of MS/MS spectra (264,251) was acquired. The MASCOT search engine was then used to identify tryptic peptides using various in-house *D. deserti* polypeptide databases that will be detailed below. Up to 11,129 unique peptides were identified when stringent search parameters were applied, corresponding to 1,348 proteins (Tables S2 and S3). Such 3D-fractionation resulted in an increased number of protein identifications compared to a single 1D SDS-PAGE shotgun, as well as a better protein sequence coverage with an average of 27% of the protein sequences determined by MS/MS.

### Genome annotation guided by proteome data: a continuous back and forth strategy

**Figure 2:**
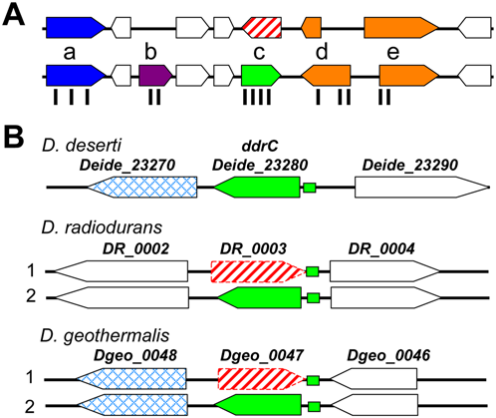
Gene prediction corrections revealed by proteomics and comparative genomics. (A) Schematic representation of the proteogenomic annotation strategy of *D. deserti* genome. First, genes were automatically predicted (upper part). Peptides detected by MS, represented by vertical black bars, were then located on the corresponding nucleic acid loci (lower part). Manual validation revealed various cases: (a) validation of predicted genes, (b) detection of non-predicted genes, (c) reversal of gene orientation, and (d, e) correction of start codons. (B) Reversal of *ddrC* gene orientation in *D. radiodurans* and *D. geothermalis*. Several data revealed that the orientation of *DR_0003* and *Dgeo_0047* (red broken arrows) has to be reversed, resulting in genes highly homologous to correctly predicted *Deide_23280* (green arrows). Previous and corrected annotations are labeled 1 and 2, respectively. Green boxes represent the radiation response motif upstream of correct *ddrC* genes. Flanking genes *Deide_23270* and *Dgeo_0048* are homologs.

The complete bacterial genome was automatically searched for CDSs with FrameD, software well suited for gene prediction in GC-rich bacterial genomes [28]. For proteogenomic annotation (Figure 2A), two polypeptide databases were constructed: a first version of CDS list (CDS1) containing 3,051 genes predicted by FrameD, and an open reading frame (ORF) list, ORF0. The latter comprised all six reading frames (65,801) with at least 100 nucleotides of length that were translated. We searched the two polypeptide databases with a preliminary corpus containing 33,480 MS/MS spectra. A large difference between the two lists of identified proteins was observed, i.e. 344 proteins found in CDS1 and 423 in the ORF0 database, indicating that approximately one fifth of the genes were not correctly predicted with the settings chosen for FrameD inspection. These results led us to further annotate intergenic regions with the recently developed Med 2.0 algorithm [29]. A second version of CDS list (CDS2) was established and contained 486 additional polypeptides. The CDS2 and ORF0 polypeptide databases were searched with the final corpus of MS/MS spectra that was collected in the meantime. Again, some differences between the two lists of identified proteins were observed, leading to the discovery and manual validation of 15 new genes. We further manually inspected intergenic regions and found 21 additional genes. Finally, we manually checked the annotation and translational start codons of the entire set of genes to create a refined CDS database, CDS3, containing 3,455 CDSs (Table 1). Using CDS3, 1,348 proteins were validated with stringent search parameters (Tables S2 and S3). These shotgun data indicate that at least 40% of the genes were expressed in the standard growth conditions at a sufficient level to be detected by MS. Among the 3,455 genes found in the *D. deserti* genome, 720 are predicted to code for conserved hypothetical proteins and 350 for orphans (Table 1). Our MS data definitively validate the expression of 234 of the former and 44 of the latter (Table S2).

We checked more specifically for N-terminal peptides in the CDS3 and ORF0 lists and verified their adequacy with predicted translational start codons. We found 212 distinct peptide signatures corresponding to 145 N termini of proteins (Table S3). These data confirmed the starts of 112 proteins but also corrected the starts of 33 polypeptides that were incorrectly predicted even after manual inspection.

### Reversal of direction of several ORFs upgrades DNA damage response knowledge

Besides detecting non-predicted coding sequences and correcting start codons, comparing theoretical annotation and MS data led to the discovery of another unexpected computational prediction error in *D. deserti* (Figure 2A). Deide_19980 was first predicted on the forward strand. As four identified peptides correspond exactly to the polypeptide encoded on the reverse strand at the same locus, we corrected the orientation of this gene and replaced *Deide_19980* with *Deide_19972* in the opposite orientation. Remarkably, comparative genomics with *D. radiodurans* and *D. geothermalis* suggests various prediction errors in these species as well (Table 2). For example, we found four different peptides substantiating the existence of Deide_15980, initially thought to be a *D. deserti*-specific protein (Figure S1). However, protein sequences highly homologous to Deide_15980 are found when *DR_0869* (*D. radiodurans*) and *Dgeo_0511* (*D. geothermalis*) are translated in the reverse direction. Therefore, *DR_0869* and *Dgeo_0511* should be reassessed, possibly reversing their orientation. *D. deserti*'s genome was further manually scrutinized for such errors. For 35 *D. deserti* genes, previously unpredicted homologs were identified in *D. radiodurans* and/or *D. geothermalis*, and these include nine additional instances of reversal of gene orientation (Table 2). Twenty-six of the conserved but differently annotated loci are present in each of the three sequenced *Deinococcus* genomes. Interestingly, two of these correspond to DNAdamage response genes *ddrC* (*DR_0003*; *Dgeo_0047*) and *ddrH* (*DR_0438*). Their gene products have not been characterized, but *DR_0003* has been inactivated, resulting in decreased radiation resistance [11]. Homologs of the reported DdrC and DdrH proteins were not found in the *D. deserti* protein database. However, when the orientations of *DR_0003* and *Dgeo_0047* are reversed, proteins highly homologous to Deide_23280 are found (Figures 2B and S2). The data strongly suggest that *DR_0003* and *Dgeo_0047* were incorrectly annotated and that *Deide_23280* and its homologs are the correct genes. Indeed, Deide_23280 and homologs are much more similar to each other than the DR_0003 homologs (Figure S2); specific RT-PCR experiments showed that *Deide_23280* is transcribed in *D. deserti* (Figure S3); and a palindromic motif identified in the upstream regions of genes that are induced upon radiation/desiccation [8] is present upstream of *Deide_23280* and its two homologs (Figure 2B). For *ddrH*, a homolog of DR_0438 was not found in *D. geothermalis* [8]. However, if *DR_0438* is transcribed from the opposite DNA strand, homologs are found in *D. geothermalis* (Dgeo_0322) and *D. deserti* (Deide_20641). These protein similarities suggest that *Deide_20641* and *Dgeo_0322* are the correctly annotated *ddrH* (Figure S4). The sequences of the *bona fide* DdrC, DdrH and the other newly recognized *Deinococcus*-specific proteins (Table 2) do not contain any described domain or motif. Characterization of their function and structure requires further experimental analysis.

**Table 2:** Differently annotated conserved loci in *D. deserti*, *D. radiodurans*, and *D. geothermalis*.

*D. deserti*	*D. radiodurans*	*D. geothermalis* a	Comments
***Deide_06040*** ** (262)**	reverse *DR_0908* (262)	*Dgeo_0637* (266)	conserved
*Deide_11910* (249)	reverse *DR_1566* (251)	*Dgeo_1299* (236)	8 conserved cysteines; Fe-S cluster
***Deide_13820*** ** (136)**	reverse *DR_0818* (130)	*Dgeo_0854* (133)	*Deinococcus*-specific
*Deide_14940* (331)	reverse *DR_1660* (327–368)	—	conserved
***Deide_15980*** ** (192)**	reverse *DR_0869* (192)	reverse *Dgeo_0511* (185)	*Deinococcus*-specific
*Deide_16650* (73)	reverse *DR_0371* (73)	*Dgeo_1881* (73)	*Deinococcus*-specific
***Deide_18240*** ** (199)**	reverse *DR_0931* (frameshift?)	downstream *Dgeo_0497* (199)	*Deinococcus*-specific membrane protein
*Deide_20641* (82)	reverse *DR_0438* (92)	*Dgeo_0322* (82)	*ddrH*
reverse *Deide_23061* (61)	upstream *DR_2438* (59)	*Dgeo_2289* (89–171?)	hypothetical
*Deide_23280* (232)	reverse *DR_0003* (231)	reverse *Dgeo_0047* (229)	*ddrC*
*Deide_1p00840* (283)	reverse *DR_A0170* (325)	*Dgeo_2841* (304)	intradiol dioxygenase
***Deide_00870*** ** (136)**	*DR_2373* (136)	upstream *Dgeo_2056* (136)	conserved
*Deide_01520* (60)	upstream *DR_2353* (60–130)	upstream *Dgeo_0094* (62)	*Deinococcus*-specific membrane protein
***Deide_02110*** ** (236)**	*DR_2585* (227)	*Dgeo_0028* (695nt); not in Genbank; frameshift?	conserved
***Deide_02380*** ** (106)**	downstream *DR_2376* (>42?)	*Dgeo_0116* (94)	*Deinococcus*-specific
*Deide_03200* (56)	downstream *DR_1936* (56)	*Dgeo_0456* (56)	*Deinococcus*-specific membrane protein
*Deide_03861* (126)	*DR_1855* (126)	upstream *Dgeo_1580* (125)	*comEA*
*Deide_04640* (120)	upstream *DR_2629* (109)	—	membrane protein
*Deide_05260* (62)	—	downstream *Dgeo_0866* (66)	*Deinococcus*-specific
***Deide_07560*** ** (51)**	between *DR_1662*/*DR_1663* (46)	upstream *Dgeo_0448* (50)	*Deinococcus*-specific
*Deide_09630* (147)	downstream *DR_1062* (218?)	*Dgeo_0746* (144)	conserved membrane protein
***Deide_10900*** ** (164)**	*DR_0846* (175)	upstream *Dgeo_1265* (167–178)	peroxiredoxin
***Deide_11240*** ** (103–162?)**	between *DR_2165*/*DR_2166* (103–346?)	*Dgeo_1280* (170?)	rhodanese-like
***Deide_11250*** ** (92)**	*DR_0570* (93)	upstream *Dgeo_1280* (101)	rhodanese-like
*Deide_13440* (57)	upstream *DR_2194* (57)	*Dgeo_1152* (57)	*lysW*
***Deide_14300*** ** (122)**	*DR_1990* (129)	downstream *Dgeo_0594* (123)	*Deinococcus*-specific; signal peptide
*Deide_14920* (163)	—	downstream *Dgeo_0948* (162)	signal peptide; SCP domain
***Deide_17020*** ** (290)**	upstream *DR_2201* (286–303)	*Dgeo_1405* (285)	conserved
***Deide_17971*** ** (53)**	*DR_1463* (97) (too long? 61 aa?)	downstream *Dgeo_1389* (49)	*Deinococcus*-specific
*Deide_19480* (133)	upstream *DR_2342* (99–289)	*Dgeo_2141* (137)	*fur*
***Deide_19965*** ** (63)**	—	upstream *Dgeo_0366* (63)	*Deinococcus*-specific
*Deide_20690* (83)	upstream *DR_0433* (84)	upstream *Dgeo_0316* (82–105)	*Deinococcus*-specific
***Deide_1p01660*** ** (346)**	—	*Dgeo_2813* (920nt); not in Genbank; frameshift?	mannonate dehydratase
***Deide_2p00970*** ** (81)**	—	downstream *Dgeo_1505* (78)	Deinococcus-specific
*Deide_2p00990* (444)	—	downstream *Dgeo_0929* (450)	erythromycin esterase
***Deide_2p01000*** ** (227)**	—	downstream *Dgeo_0931* (220)	phosphoribosyltransferase

a For the first 11 loci in this table, the orientation of one or two of the predicted genes has to be reversed. For the other loci, a gene was not predicted in one or two species, but found near the indicated locus. The products of the 18 *D. deserti* genes indicated in bold face were identified in the proteome analysis after standard cultivation. Lengths of gene products are indicated between parentheses.

### Genome comparisons and *Deinococcus*-specific genes

**Figure 3:**
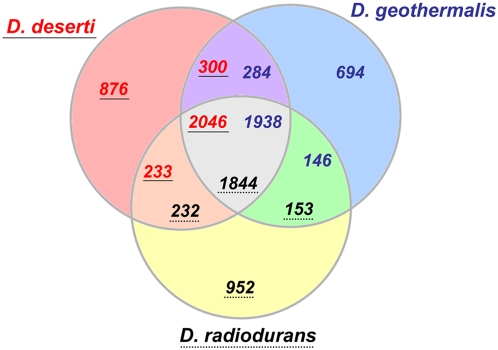
Orthologous gene comparison between the three sequenced *Deinococcus* strains. Orthologous genes are defined by BLAST search when their gene products shared a minimum of 30% identity and 70% coverage. The circle intersections give the number of genes found in two or three of the compared species, including paralogous CDSs. Numbers of genes specific to each species are represented outside these areas. *D. deserti* gene numbers are in red and underlined, those of *D. radiodurans* are in black with dotted underlining, and those of *D. geothermalis* are in blue and not underlined.

Entire genome comparisons showed that 2,046 of the predicted gene products of *D. deserti* are homologous (at least 30% identity and 70% coverage) to proteins from both *D. radiodurans* and *D. geothermalis* (Figure 3). These correspond to 1,844 and 1,938 proteins in *D. radiodurans* and *D. geothermalis*, respectively, showing that *D. deserti* globally has more paralogous CDSs. Multiple paralogs are found for exported S8 peptidases, exported serine proteinases, cold shock proteins, and several DNA repair proteins (see below). For example, each of the three cold shock proteins Deide_09930, Deide_2p00490 and Deide_3p00840 is homologous to only one protein of *D. radiodurans* (DR_0907) and two of *D. geothermalis* (Dgeo_0638 and Dgeo_1006). For 876 *D. deserti* proteins, no homolog was detected in *D. radiodurans* or *D. geothermalis* (Figure 3).

Pairwise comparisons of the different replicons of *D. deserti* with those of the other two *Deinococcus* species revealed strong homologous relationships (Table S4) and significant levels of gene order conservation (Figure S5) between the main chromosomes. As determined by reciprocal best hit analysis, the chromosome of *D. deserti* shares 1,686 and 1,804 gene homologs respectively with those of *D. radiodurans* and *D. geothermalis*. Although each of the three *D. deserti* plasmids contain genes and gene clusters that are conserved in *D. radiodurans* and *D. geothermalis*, *D. deserti* plasmid P2 appeared to have the highest levels of homology with *D. geothermalis* plasmid pDSM11300 (DG574) and with *D. radiodurans* chromosome 2 (Table S4). No or very little homology was observed between *D. deserti*, and *D. radiodurans* plasmid pCP1 or *D. geothermalis* plasmid pDGEO02. Overall, a reciprocal best hit with *D. radiodurans* and/or *D. geothermalis* was found with 80, 35, 65 and 40% of the genes present on the *D. deserti* chromosome and plasmids P1, P2, and P3, respectively. Among those without reciprocal best hits are genes related to functions such as signal transduction and regulation, cell envelope biogenesis, DNA repair, nutrient metabolism and transport.

The distinctive characteristics of *Deinococcus* bacteria are likely in part determined by proteins that are unique in this genus. We have identified 230 proteins, mostly of unknown function and including six identified after reversal of gene orientation, that are specifically conserved in the three sequenced *Deinococcus* genomes (Table S5). A role in radiotolerance has been demonstrated for a few of these, e.g. IrrE (also called PprI) [30],[31] and PprA [15]. Predicted membrane and exported proteins, accounting for 39% of the *Deinococcus*-specific proteins, may contribute to cell envelope integrity, stress tolerance and viability. Indeed, two desiccation resistance-associated proteins (Deide_07540 and Deide_09710) have a predicted signal-peptide and are likely functional outside the cytoplasm. Of the 230 *Deinococcus*-specific proteins, 92 were detected in our proteome analysis (Table S5), many among the highly expressed proteins, strongly suggesting their importance in general metabolism and perhaps cell viability and stress resistance in *D. deserti*.

### Adaptation to extreme environment

*D. radiodurans* has very high intracellular manganese and low iron levels [32], which are correlated with protection of proteins from oxidative modifications [16]. *D. deserti* was also found to accumulate Mn, with Mn/Fe ratios of 0.54 and 1.05 in two different growth media (Table S6). These ratios were even higher than found with *D. radiodurans* in the same experimental conditions: 0.16 and 0.47, respectively. We checked for the presence of Mn- and Fe-transport and regulation related-systems in *D. deserti* (Table S7). While only one ABC-type Mn/Zn transport system, composed of an ATPase, a permease and a periplasmic component, is present in *D. radiodurans* and *D. geothermalis*, several homologs of these components are encoded in *D. deserti*: three ATPases, five permeases and three periplasmic components. A distantly related homolog of the Nramp family of Mn(II) transporters was also found. The Mn-dependent transcriptional regulator TroR/DtxR is triplicated in *D. deserti*. For Fe-homeostasis, some differences were observed between the three *Deinococcus* strains (Table S7). The specific presence in *D. deserti* genome of an operon comprising two homologous genes specifying siderophore synthetase components is remarkable. Several differences were also observed for proteins related to oxidative stress response (Table S8). Four SoxR-related transcriptional regulators occur in *D. deserti* while only one and two are found in *D. geothermalis* and *D. radiodurans*, respectively.

Like *D. radiodurans* and *D. geothermalis*, *D. deserti* contains four close homologs of plant desiccation resistance-associated proteins (Table S8). Interestingly, three of these were detected in *D. deserti* after standard cultivation, suggesting their importance for swift adaptation to rapid environmental changes occurring in the hot desert. The products of many other stress response-related genes were also detected in the proteome analysis (Table S8).

Besides having more Mn ABC transporter proteins, *D. deserti* is also rich in ABC transporters for oligopeptides, amino acids and sugars in comparison to *D. radiodurans* and *D. geothermalis* (Table S9). Thirty-one of 90 amino acid and peptide transporter proteins are specific to *D. deserti*, as are 17 of 54 proteins for sugar transport. Several components of these ABC transporters were detected by the proteome analysis (Table S9), showing that they are used by the bacterium in standard cultivation conditions. A large diversity in ABC transporters is likely important for *D. deserti*'s adaptation to the desert where nutrients are limiting, and for osmoprotection in the case of glycine/betaine transporters.

### *D. deserti* genome contains three *recA* and three TLS polymerase genes

*D. deserti* has many DNA repair genes in common with *D. radiodurans* and *D. geothermalis* (Table S10), but several interesting specific *D. deserti* features were also observed (Table 3). Surprisingly, three different *recA* genes were found. RecA is a key component in DNA repair, and required for extreme radiation tolerance in *D. radiodurans*. Most other known bacterial species possess a single *recA*. The presence of multiple *recA* genes has been reported for only three species: two in *Myxococcus xanthus* [33] and *Bacillus megaterium* [34], and seven in the cyanobacterium *Acaryochloris marina* [35]. The *recA* genes on plasmid P1 and P3 code for identical proteins. They share 81% identity with the chromosome-encoded RecA. As in *D. radiodurans* and *D. geothermalis*, the chromosomal *recA* of *D. deserti* is the last gene in an operon that also contains *cinA* and *ligT*, encoding a DNA damage/competence-inducible CinA-like protein and a 2′-5′ RNA ligase, respectively. For both the chromosome-encoded RecA (90% identical residues) and the plasmid-encoded RecA (80% identical residues), the best hits in BLAST analyses were the RecA proteins from *D. radiodurans* and *D. geothermalis*. Moreover, unlike the other two Deinococci, *D. deserti* possesses three putative translesion synthesis (TLS) DNA polymerases: a Pol II homolog (PolB); a protein distantly related to members of the DinB subfamily of Y-family DNA polymerases; and DnaE2, a potential error-prone DNA polymerase homologous to the alpha subunit DnaE of the major replicative DNA polymerase III (Table 3). In addition, the *D. deserti* genome encodes a protein related to DNA repair photolyases, a 5′-3′ exonuclease distantly related to *Escherichia coli* exonuclease IX, and a large multidomain helicase (Deide_06510) also found in *Thermus thermophilus* HB27. There are also two DnlJ DNA ligases sharing 57% identity while the other Deinococci contain only one *dnlJ* gene. *D. deserti* also possesses two genes encoding proteins highly homologous to DdrO (DR_2574), a proposed regulator of the radiation response regulon in *D. radiodurans* [8], Deide_20570 and Deide_3p02170 sharing 95% and 85% identity respectively with DR_2574. On the contrary, *D. deserti* has only one *ung*/*udg* homolog for uracil-DNA glycosylase, whereas *D. radiodurans* has three and *D. geothermalis* two (Table 3). Four *ssb* gene homologs were detected in *D. geothermalis*, while only one is present in the other two Deinococci.

**Table 3:** Main differences among *Deinococci* regarding DNA repair proteins.

*D. deserti*	*D. radiodurans*	*D. geothermalis*	Protein name and description
Deide_19450 (355), Deide_1p01260 (344), Deide_3p00210 (344)	DR_2340 (363)	Dgeo_2138 (358)	RecA
Deide_1p00180 (765)	**—**	**—**	DNA polymerase II
Deide_1p01880 (423)	**—**	**—**	Y-family DNA polymerase
Deide_1p01900 (1058)	**—**	**—**	error-prone DNA polymerase DnaE2
Deide_3p02150 (354)	**—**	**—**	DNA repair photolyase
Deide_20570 (129), Deide_3p02170 (129)	DR_2574 (131)	Dgeo_0336 (140)	DdrO; transcriptional regulator
Deide_11320^a^ (731)	DR_1289^b^ (824)	Dgeo_1226^b^ (195)	DNA helicase RecQ
Deide_1p01280^b^ (548)	**—**	**—**	DNA helicase, RecQ family
Deide_06510^b^ (1683)	**—**	**—**	Multidomain protein: DnaQ/DinG/RecQ
Deide_06520 (849)	**—**	**—**	UvrD/REP helicase
Deide_17790 (202), Deide_05970 (204)	DR_0856 (197)	Dgeo_1818 (180)	DnaQ
Deide_12290 (686), Deide_1p00290 (686)	DR_2069 (700)	Dgeo_0696 (684)	DNA ligase, NAD-dependent
Deide_1p00132 (244)	**—**	**—**	5′-3′ exonuclease
Deide_00830 (248)	DR_0689 (247), DR_1751 (237), DR_0022 (199)	Dgeo_2059 (245), Dgeo_1556 (230)	Uracil-DNA glycosylase (Ung/Udg)
Deide_00120 (297)	DR_0099 (301)	Dgeo_0165 (301), Dgeo_2964 (283), Dgeo_2969 (352), Dgeo_3087 (200)	Single-stranded DNA-binding protein

Polypeptide lengths are indicated between parentheses.

a Two HRDC domains were found in Deide_11320, three in DR_1289, and one in Dgeo_1226.

b No detectable HRDC domain.

Among the 92 DNA repair and radiation tolerance-associated proteins of *D. deserti* listed in Table S10, 36 were identified in the proteome analysis, indicating detectable basal levels of these proteins in the absence of exposure to exogenous DNA damaging agents. Among these are DNA glycosylases, nucleotide excision and recombinational repair proteins, several “house-cleaning” Nudix hydrolases, both DNA ligases, and the DNA repair regulator and putative sensor protein IrrE (PprI). Such basal cellular levels of DNA repair proteins might be expected if DNA damage occurs frequently due to generation of high levels of metabolism-derived reactive oxygen species or if cells must be constantly ready to quickly respond to external stress.

**Figure 4:**
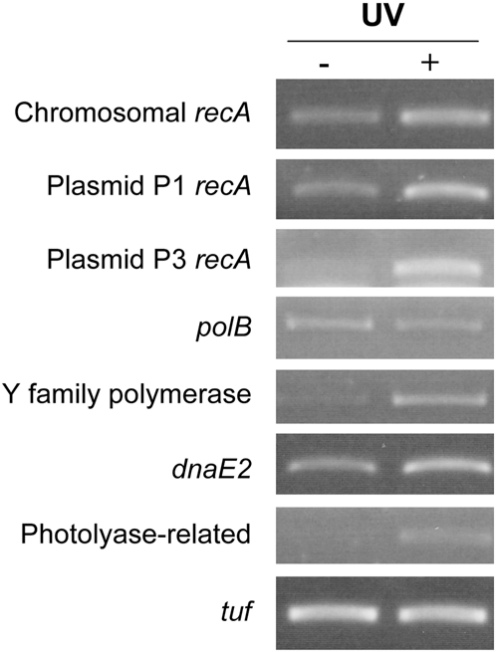
RT-PCR analysis of *recA* and putative translesion DNA polymerase and photolyase genes. RNA was isolated 30 min after exposure to 0 (−) or 250 (+) J.m^−2^ UV. The constitutively expressed *tuf* gene was included as control.

The chromosome-encoded RecA was detected by the proteome analysis, whereas peptides that specifically demonstrate the presence of plasmid-encoded RecA were not identified. Similarly, none of the TLS polymerases was detected after standard cultivation. However, RT-PCR experiments showed that the three *recA*, the three TLS polymerase genes and the photolyase-related gene were transcribed. Moreover, except for *polB*, transcription of each of these genes was induced after exposure to UV (Figure 4), indicating a role in the DNA damage response in *D. deserti*.

### The *Deinococcus* radiation response regulon is conserved

A potential common radiation response regulon in *D. radiodurans* and *D. geothermalis* identified by a palindromic motif has been reported [8]. This radiation/desiccation response motif is found in the upstream regions of radiation-induced genes such as *recA*, *ddrA*, *ddrO*, *pprA*, *uvrA*, and *gyrA*. Induction of transcription of at least *recA* and *pprA* is IrrE (PprI)-dependent in *D. radiodurans* [30],[31]. We searched for such motif in *D. deserti* and detected its signature in the upstream regions of the same genes and, in addition, upstream of *Deide_23280* (the *bona fide ddrC*) and the two plasmid-encoded *recA* genes (Table S11). Therefore, the radiation response regulon is a general trait of *Deinococcus* species. This is further supported by the observation that *D. deserti* IrrE fully restored radiation resistance when expressed in a *D. radiodurans irrE* deletion mutant [36]. The motif was not found near the other additional *D. deserti* DNA repair genes listed in Table 3, including those specifying the photolyase-related protein and TLS DNA polymerases. These may be regulated in another manner. Interestingly, the palindromic motif was also found near several genes that have no obvious relation with DNA repair, notably near *Deide_18730*, the first of five uncharacterized genes in a putative operon that is conserved in various species but absent from *D. radiodurans* and *D. geothermalis*.

## Discussion

*D. deserti* was isolated from the surface sand of the Sahara, an extreme environment, where it is exposed to harsh conditions with concomitant UV irradiation, desiccation and nutrient limitation. To learn more about the adaptation of *D. deserti* to arid desert as well as the remarkable tolerance to radiation and desiccation of *Deinococcaceae* in general, we determined the sequence of its entire genome, composed of a chromosome and three large plasmids. Moreover, accurate gene annotation was guided by extensive proteome analysis after growth of *D. deserti* under standard conditions. Besides validation of about 40% of the predicted genes, peptide data allowed correction of several initiation codons, identification of unpredicted genes and, surprisingly, reversal of incorrectly predicted gene orientation. Comparative genomics and proteomics showed that the orientation of several annotated *D. radiodurans* and *D. geothermalis* genes should also be reversed. This is an important matter and to our knowledge has never been reported previously at the genomic scale. Interestingly, transcription of two of these, *ddrC* and *ddrH*, was induced in *D. radiodurans* after exposure to radiation and desiccation [11]. These transcription data may seem contradictory to our results. However, we believe that Tanaka *et al.* [11], without awareness, measured transcription of the correct *ddrC* and *ddrH* genes. Their transcription analyses could not distinguish between one DNA strand and its complementary strand since the DNA spotted on the microarrays were obtained by PCR, and thus included both DNA strands. Moreover, random hexamers were used for initiation of cDNA probe synthesis (A. Earl, personal communication), and these hexamers annealed with any RNA, including the correct *ddrC* and *ddrH* mRNA. Several other previously unannotated genes were found in *D. geothermalis* and/or *D. radiodurans*, including homologs of *Deide_17971* and *Deide_19965*, genes that were not predicted but whose products were detected by proteomics. These and 5 other genes that were discovered by proteomics, code for very small polypeptides of 4.5–9.6 kDa. Our work shows that a combination of high-throughput proteomic and genomic techniques allows the most accurate genome annotation presently obtainable. By extension, the annotation of the whole *Deinococcus-Thermus* group may be revisited taking into account evolutionary constraints, a concept already applied to the genera *Shewanella* and *Mycobacterium* [20],[21].

Besides proteogenomic annotation, shotgun proteomics allows semi-quantitation of the major proteins by spectral counting. Since the demonstration by Liu *et al.* [37] that spectral counts of MS/MS spectra for a given protein in a mixture correlated linearly with protein abundance within over two orders of magnitude, numerous semi-quantitative studies have been based on this concept [38]. Among the 264,251 MS/MS spectra that were acquired, 50,461 spectra were confidently assigned to a peptide sequence. Averages of 4.5 spectra per unique peptide and 37 spectra per protein were reached taking into consideration the global set of data. The redundancy is even higher for abundant proteins. Although these data are only semi-quantitative and extracted from the entire set of MS/MS spectra recorded in this study, several interesting features should be noted. In our MS/MS corpus, 50% of the spectra were attributed to 106 proteins, 75% to 281 proteins, and 90% to 547 proteins (Table S2). Interestingly, among the highly detected proteins are several orphans (Deide_3p02433, Deide_15630, and Deide_11191), *Deinococcus*-specific proteins (Deide_06180, Deide_1p01253, Deide_21340, Deide_11730, Deide_16050, Deide_16110, Deide_09460, Deide_01434, and Deide_15100) and more widely conserved proteins (Deide_3p01280, Deide_21050, Deide_13500, Deide_04930, and Deide_14540) of unknown function, suggesting an important role in *D. deserti*. The proteome data indicate that plasmids P1 and P3 are underused in our standard growth conditions in comparison to P2 and the chromosome. The products of 17–19% of the genes present on P1 and P3 were detected, compared to 34–45% of those on the chromosome and P2. These data suggest that most of the P1 and P3 genes may be used under more specific conditions (such as in the desert and/or after stress) than the laboratory conditions used here.

Extreme tolerance to radiation and desiccation in Deinococci requires efficient DNA repair. In addition to most of the classical DNA repair genes, the previously identified novel, *Deinococcus*-specific genes involved in DNA repair and radiotolerance, i.e. *ddrA-D*, *pprA* and *irrE* (*pprI*), are all conserved in *D. deserti*. In addition, using epifluorescence microscopy, we have observed that *D. deserti* (results not shown), like other radioresistant bacteria [39], has a highly condensed nucleoid, which may restrict diffusion of radiation/desiccation-generated DNA fragments. Furthermore, in common with *D. radiodurans* and *D. geothermalis*, *D. deserti* has a high Mn/Fe ratio, important for protein protection.

Besides the common set of genes and characteristics involved in protection or repair, each species has probably also evolved specific functions to adapt to its environment. This is supported by the identification of several genes for which a role in stress resistance and DNA repair has been shown or can be expected, but that are not shared by the Deinococci. For example, homologs of *D. radiodurans irrI*, associated with radiation resistance [40], and several radiation-induced *ddr* genes [11], are absent in *D. deserti* and *D. geothermalis* (Table S10). As another example, the conserved radiation response motif was found upstream of two *D. deserti* genes of unknown function with a homolog only in *D. geothermalis* (*Deide_04721*) or without homologs in the other Deinococci (*Deide_18730*). The latter is the first gene of a putative operon encoding five uncharacterized proteins, of which Deide_18710 (related to MoxR-like AAA+ ATPases) could function with Deide_18690 (containing a Von Willebrand Factor Type A domain) as a chaperone system for the folding/activation of specific substrate proteins [41].

Another interesting feature is the presence in *D. deserti* of TLS DNA polymerase genes, as well as a photolyase-related gene and two supplementary *recA*, all present on plasmid P1 or P3. All of these genes could be involved in tolerance to UV-light. *E. coli* RecA plays a major role in recombinational repair of UV-lesions, has a regulatory role in lesion bypass through its coprotease activity, which includes stimulation of self-cleavage of repressor protein LexA, and participates directly in lesion bypass by interacting with TLS Pol V [42],[43]. As the three TLS DNA polymerase genes in *D. deserti* are located on plasmid P1, it is tempting to speculate that the plasmid-encoded RecA is involved in regulation and/or activation of one or more of these TLS polymerases. Moreover, a *lexA* homolog, *Deide_1p01870*, is adjacent and possibly in an operon with the TLS DNA polymerase genes *Deide_1p01880* (Y-family DNA polymerase) and *Deide_1p01900* (DnaE2). Similar so-called adaptive mutagenesis gene cassettes have been recently described and shown to be under RecA/LexA regulation in several bacterial species [44], and a role for the Y-family DNA polymerase and/or DnaE2 in survival and induced-mutagenesis after exposure to DNA-damaging conditions has been demonstrated in *Mycobacterium tuberculosis* [45], *Caulobacter crescentus* [46] and *Pseudomonas putida* [47].

Taken together, the supplementary nutrient import and DNA repair genes in *D. deserti* may contribute to survival in nutrient-poor extreme conditions. The additional DNA repair genes likely provide an advantage in an environment where many DNA lesions can be generated due to intense UV irradiation and desiccation. Moreover, TLS polymerases generate mutations that will increase genetic diversity, which may lead to better adaptation to harsh conditions, such as those encountered in the desert [48].

## Materials and Methods

### Genome sequencing

The complete sequence of *D. deserti* VCD115 was determined after genomic DNA fragmentation by mechanical shearing or BamHI partial digest for the construction of plasmid and large insert libraries, respectively. The 3 kb (A), 10 kb (B) and 25 kb (C) fragments were cloned into pcDNA2.1 (INVITROGEN), pCNS (pSU18 derived) and pBeloBac11, respectively. Vector DNAs were purified and end-sequenced (49536 (A), 16128 (B) and 7680 (C)) using dye-terminator chemistry on ABI3730 sequencers. A pre-assembly was made without repeat sequences as described [49] using Phred/Phrap/Consed software package (www.phrap.org). The finishing step was achieved by primer walking and PCR. A complement of 665 sequences was needed for gap closure and quality assessment.

### Gene prediction and annotation

Protein-coding regions in the assembled genome sequence were identified using FrameD [28] and MED [29]. Predicted proteins larger than 10 amino acids were analysed for sequence similarity against protein databases (SWISSPROT, TREMBL and non-redundant GenBank proteins). Similarity searches were carried out using BLAST programs [50]. Annotation of the complete genome was performed using GenoBrowser, an in-house bioinformatic tool for data management (Ortet et al., submitted). Our tool allows an expert annotation by manual verification and curation of functional protein categories after automatic assignment. Genome regions without match were re-evaluated by using BLASTX as initial search, and ORFs were extrapolated from regions of alignment. rRNA and tRNA genes were identified with BLASTN and tRNA-Scan [51]. miscRNA were identified using INFERNAL software suite with RFAM database [52] version 8.1 containing 607 families. Repeats were identified using RepSeek [53].

### Genbank submission

The annotated genome sequence of *D. deserti* VCD115 was deposited in the GenBank database with accession numbers CP001114, CP001115, CP001116 and CP001117 for the main chromosome, plasmids P1, P2 and P3, respectively.

### Comparative genomics

All predicted *D. deserti* proteins were aligned using BLASTP against the available proteomes of published complete bacterial genomes (625 genomes). The results of these searches, requiring bi-directional best hit (BBH) among all possible pairwise comparisons, were used to determine the presence of homologs in different species.

### Proteome fractionation by phenyl sepharose and SDS-PAGE

*D. deserti* cells grown in tenfold diluted tryptic soy broth (TSB/10) supplemented with trace elements [36] and harvested in exponential growth and in pre-stationary phase (4 g of wet material) were resuspended in 20 mL of cold 100 mM TRIS/HCl buffer (pH 8.0 at 20°C) containing 5 mM EDTA, disrupted and centrifuged. Proteins from 8 mL of both resulting supernatants were subjected to ammonium sulphate precipitation. Proteins still soluble after addition of 50% final (NH_4_)_2_SO_4_ were precipitated with 67% final (NH_4_)_2_SO_4_. The resulting four equivalent pellets were resuspended in 50 mM TRIS/HCl buffer pH 8.0, containing 2.5 mM EDTA and 1.5 M (NH_4_)_2_SO_4_ (Buffer A). Proteins from each of the four samples were applied at a flow rate of 1.0 mL/min onto a 5 mL HiTrap Phenyl HP column (Amersham Biosciences) previously equilibrated with Buffer A. After wash with Buffer A, proteins were eluted over a 60 mL linear gradient comprising 1.5–0 M (NH_4_)_2_SO_4_. Eluted proteins (24 fractions per phenyl chromatography) were precipitated by trichloroacetic acid (10% final) and collected by centrifugation. Proteins were dissolved in LDS sample buffer (Invitrogen) and then analyzed by SDS-PAGE on 4–12% gradient NuPAGE (Invitrogen) gels. The gels were stained with Coomassie Safe Blue stain (Invitrogen) and then each relevant lane was excised into 4–10 mm-thick pieces from the top to the bottom polypeptide bands.

### In-gel proteolysis

Protein bands were washed with MilliQ water, treated with CH_3_CN, and then with 100 mM NH_4_HCO_3_. Gel pieces were dehydrated with 100% CH_3_CN and dried for 20 min under vacuum. For in-gel digestion, dry gel pieces were rehydrated for 45 min at 56°C with 100 mM NH_4_HCO_3_ containing 10 mM DTT. Gel pieces were rinsed with CH_3_CN, treated with 100 mM NH_4_HCO_3_ containing 55 mM iodoacetamide, then dehydrated with 100% CH_3_CN and dried for 20 min under vacuum. For in-gel digestion, dry gel pieces were rehydrated with 12 ng/µL trypsin in 25 mM NH_4_HCO_3_ (pH 8.5) containing 1% CaCl_2_. After overnight proteolysis at 37°C, digests were extracted first with 100% HCOOH, then with 56% CH_3_CN/1% HCOOH, and finally with 100% CH_3_CN. The resulting pools were dried completely under vacuum and stored at −20°C until MS analysis.

### LC-MS/MS analysis

LC-MS/MS experiments were performed on two equipments: (i) a Esquire 3000 plus ion trap (Bruker Daltonics) equipped with a nanoelectrospray online ion source, and coupled to a UltiMate-Switchos-Famos LC system (Dionex-LC Packings), and (ii) a LTQ-Orbitrap XL hybrid mass spectrometer (ThermoFisher) coupled to a UltiMate 3000 LC system (Dionex-LC Packings). Peptide mixtures (0.5–5 pmol) were loaded and desalted online in a reverse phase precolumn (C18 Pepmap column, LC Packings), and resolved on a nanoscale C18 Pepmap TM capillary column (LC Packings) at a flow rate of 0.2–0.3 µL/min with a gradient of CH_3_CN/0.1% formic acid prior injection in the ion trap mass spectrometer. Peptides were separated using a 90 min-gradient from 5 to 95% solvent B (0.1% HCOOH/80% CH_3_CN). Solvent A was 0.1% HCOOH/5% CH_3_CN for the Esquire nanoLC system, and 0.1% HCOOH/0% CH_3_CN for the LTQ-orbitrap nanoLC system. The full-scan mass spectra were measured from m/z 50 to 2000 with the Esquire ion trap mass spectrometer, and m/z 300 to 1700 with the LTQ-orbitrap XL mass spectrometer. The latter was operated in the data-dependent mode using the TOP7 strategy. In brief, a scan cycle was initiated with a full scan of high mass accuracy in the orbitrap, which was followed by MS/MS scans in the linear ion trap on the 7 most abundant precursor ions with dynamic exclusion of previously selected ions. A total of 326 and 40 samples were analyzed on the mass spectrometers, resulting in 163,779 and 100,472 MS/MS spectra recorded, respectively.

### Polypeptide database MS/MS search

Using the MASCOT search engine (Matrix Science), we searched all MS/MS spectra against home made polypeptide sequence database. Searches for trypsic peptides were performed with the following parameters: full-trypsin specificity, a mass tolerance of 5 ppm on the parent ion and 0.5 Da on the MS/MS (LTQ-orbitrap mass spectra) or 0.4 Da for the parent ion and 0.5 Da for the MS/MS (Esquire ion trap mass spectra), static modifications of carboxyamidomethylated Cys (+57.0215), and dynamic modifications of oxidized Met (+15.9949). The maximum number of missed cleavage was set at 1. All peptide matches with a Peptide Score of at least 31 (average threshold for p<0.007 with the final database search using the Esquire ion trap data) and rank 1 were filtered by the IRMa 1.16.0 software (J. Garin&C. Bruley, CEA, DSV, iRTSV, Grenoble). The criteria adopted for protein identification were very conservative with either 1) that at least two peptides with ion score 31 or higher match or 2) that at least one peptide with ion score 50 (average threshold for p<0.0001) or higher matches. A False-Positive rate of 0.2% was estimated using the corresponding decoy database. Further data analysis was performed for semi-trypsin specificity (p<0.001). Spectral count (number of spectra recorded per protein) was performed on the whole set of MS/MS spectra recorded over the entire study. Proteins were then evaluated for their respective abundance after molecular weight normalization.

### Kinetics of DNA repair after gamma-irradiation or desiccation-rehydration

For gamma irradiation, cells were grown at 30°C in TSB/10 to an OD_600_ of 0.5, concentrated to an OD_600_ of 25, and irradiated on ice to 6,800 Gy at 56.6 Gy/min dose rate (^137^Cs source). After irradiation, cultures were diluted to an OD_600_ of 0.2 and incubated at 30°C. At different post-irradiation incubation times, 5 ml aliquots were removed to prepare agarose plugs as described by Harris et al. [14]. The DNA in the plugs was digested with PmeI and SwaI restriction enzymes, and then subjected to pulsed-field gel electrophoresis for 24 h at 12°C using a CHEF MAPPER electrophoresis system (Biorad) with the following conditions: 6.0 V/cm, linear pulse ramp of 60–120 s, and a switching angle of 120° (−60° to +60°). For desiccation, stationary phase cells were concentrated to an OD_600_ of 20 and placed on sterile glass slides (100 µl cells per slide), dried and stored at room temperature in a sealed desiccator over anhydrous CaSO_4_. The CaSO_4_ desiccant is impregnated with CoCl_2_. This latter chemical compound is a visual moisture indicator: anhydrous CoCl_2_ is blue while pink in presence of water. After 27 days of desiccation, cells from 5 glass slides were resuspended in 50 ml TSB/10, incubated at 30°C and 5 ml aliquots were taken at different times and treated as described above.

### RNA isolation and RT-PCR

For analysis of gene expression after UV exposure, samples from the same exponential phase culture (OD_600_ 0.2–0.4) were exposed to either 0 or 250 J.m^−2^ of UV-C (4 mL volumes in Petri dishes), and then further incubated. Samples for RNA isolation were taken after 30 min. Cells were treated with RNAprotect Bacteria Reagent (Qiagen) prior to RNA isolation using the RNeasy Mini Kit (Qiagen) according to the manufacturer's instructions. RNA samples were treated twice with DNase. For RT-PCR, cDNA was synthesized in 20 µL reactions using 1 µg of RNA and the Transcriptor First Strand cDNA Synthesis Kit (Roche). DNA fragments of 150–250 bp were then amplified in 25 µL reactions using 1 µL of cDNA from the first step, Taq polymerase (Sigma) and gene-specific primers (Table S12). These amplifications were carried out by incubating reactions at 94°C for 10 min prior to 30 cycles of 1 min at 94°C, 30 sec at 56°C and 30 sec at 72°C, followed by a final step at 72°C for 2 min, with modifications for *tuf* (25 cycles), *recA-P3* and *dnaE2* (both 35 cycles). Controls for DNA contamination were performed with reactions lacking reverse transcriptase.

## Supporting Information

Figure S1 Evidence for correct annotation of *Deide_15980*. *Deide_15980* proteogenomic annotation with four peptides detected by mass spectrometry (A). Multiple sequence alignments of Deide_15980 and homologs found encoded in the reverse orientation of *DR_0869* and *Dgeo_0511* (B). Multiple sequence alignments of incorrectly predicted DR_0869 and Dgeo_0511 with a protein found when the orientation of *Deide_15980* is reversed (C). Identified peptides for Deide_15980 are indicated with black and blue bars in (A). The peptide sequences can be found in Table S3.
(0.07 MB PDF)

Figure S2 Evidence for correct annotation of *ddrC* in *Deinococcus* species. Schematic representation of the *ddrC* loci and flanking genes in *D. deserti*, *D. radiodurans* and *D. geothermalis* (A). Multi-alignment of polypeptide sequences for correct DdrC proteins (B) and wrongly annotated DdrC proteins (Panel C). In Panel A, the predicted genes are labeled with the locus tag number. *DR_0003* and *Dgeo_0047* are incorrectly predicted, and their orientation has to be reversed. Correct *ddrC* genes are shown in black arrows, wrong *ddrC* ORFs in open arrows. The black boxes upstream the correct *ddrC* genes indicate the radiation response motif. Flanking genes *Deide_23270* and *Dgeo_0048* are homologs.
(0.05 MB PDF)

Figure S3 Specific RT-PCR showing transcription of correct *ddrC* (*Deide_23280*). Genome region of *Deide_23280* (A). *Deide_23280* is present on the reverse DNA strand. The opposite DNA strand could encode a protein related to DR_0003 and Dgeo_0047 (indicated by the blue ellipse). Green and red bar indicate start and stop codons, respectively. Black bar indicates the radiation/desiccation response motif upstream *Deide_23280*. RV and FW indicate schematically the orientation of the primers used in the RT-PCR. Specific two-step RT-PCR (B). Either purified primer FW (Dd23280FW) or RV (Dd23280RV) was used to synthesize cDNA in the RT reaction (first step). The PCR in the second step was performed with both primers. When *Deide_23280* is the correctly predicted gene, cDNA synthesis and thus an RT-PCR product is expected with primer RV in the RT reaction. RNA was isolated 30 min after the cells were irradiated (+) or not (−) with UV (250 J/m^2^). N and P indicated negative (no template) and positive (genomic DNA) control, respectively.
(0.11 MB PDF)

Figure S4 Evidence for correct annotation of *ddrH*. Multi-alignment of correct DdrH protein sequences (A) and wrongly annotated DdrH protein sequences (B). The *D. radiodurans* protein highly homologous to Deide_20641 and Dgeo_0322 is found when the orientation of *DR_0438* (*ddrH*) is reversed.
(0.03 MB PDF)

Figure S5 Genome dot plots for the chromosomes of sequenced *Deinococcus* species. Genome dot plots for the chromosome of *D. deserti* (horizontal axis) *vs. D. geothermalis* (vertical axis) (A) and *vs. D. radiodurans* (vertical axis) (B). Each dot represents the location of a pair of Bidirectional Best Hits (with a minimum of 30% identity and 70% coverage) between the two chromosomes.
(0.22 MB PDF)

Table S1 Insertion sequences identified in the genome of *D. deserti* and comparison with other *Deinococcus*/*Thermus* species.
(0.09 MB PDF)

Table S2 Proteins identified by LC-MS/MS shotgun.
(0.26 MB XLS)

Table S3 Peptides identified by LC-MS/MS shotgun.
(2.67 MB XLS)

Table S4 Homology between the DNA molecules of *D. deserti*, *D. radiodurans* and *D. geothermalis*.
(0.07 MB PDF)

Table S5 *Deinococcus*-specific proteins.
(0.16 MB PDF)

Table S6 Mn/Fe ratio in *D. deserti* and *D. radiodurans*.
(0.08 MB PDF)

Table S7 Mn- and Fe- transport and regulation related systems in *D. deserti*.
(0.08 MB PDF)

Table S8 Stress response-related proteins in *D. deserti*, *D. radiodurans* and *D. geothermalis*.
(0.16 MB PDF)

Table S9 ABC-transporters identified in *D. deserti*.
(0.15 MB PDF)

Table S10 DNA repair genes in *D. deserti*, *D. radiodurans* and *D. geothermalis*.
(0.20 MB PDF)

Table S11 Radiation/desiccation response motif identified in *D. deserti*.
(0.11 MB PDF)

Table S12 Primers for RT-PCR.
(0.06 MB PDF)
