# Supplementary material for: Alliance of Proteomics and Genomics to Unravel the Specificities of Sahara Bacterium Deinococcus deserti
Source: PLoS Genet. 2009 Mar 27;5(3):e1000434. doi: 10.1371/journal.pgen.1000434 (PMC2669436; doi:10.1371/journal.pgen.1000434)
Supplement: Figure S1 — Evidence for correct annotation of Deide_15980. Deide_15980 proteogenomic annotation with four peptides detected by mass spectrometry (A). Multiple sequence alignments of Deide_15980 and homologs found encoded in the reverse orientation of DR_0869 and Dgeo_0511 (B). Multiple sequence alignments of incorrectly predicted DR_0869 and Dgeo_0511 with a protein found when the orientation of Deide_15980 is reversed (C). Identified peptides for Deide_15980 are indicated with black and blue bars in (A). The peptide sequences can be found in Table S3. (0.07 MB PDF) [file pgen.1000434.s001.pdf]

Figure S1

A

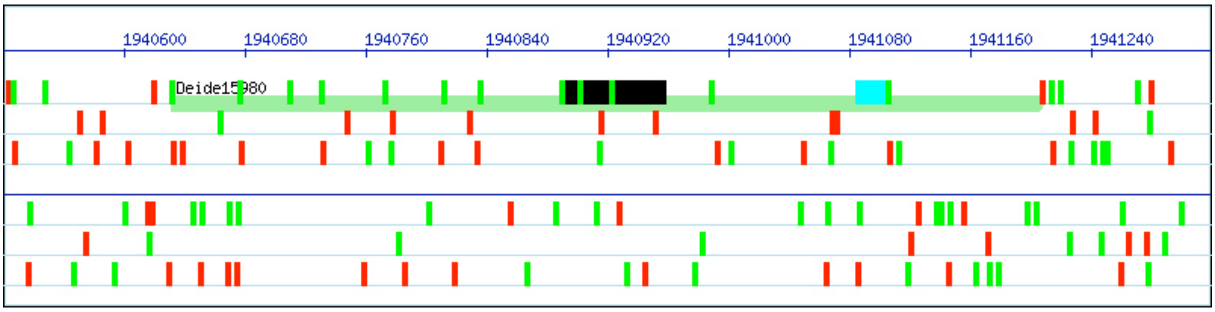

B

```
Deide_15980         MSVKDNFTADEWFKIMTGPGRAGAAVVAASPSGLTGLLAEAQAIAASVREHVSRAGRTPL 60
revDR_0869          MSVKDNFTADEWFKVMTGPGRAGAAVVAASPSGLTGLLAEAQAIAASVRESVSGTPRTPL 60
revDgeo_0511        MSVKDNFTAEEWFTVMNGPGRAGAAVVAASPSGLTGLVAEAQAIAQAVREQVSAQGRTPL 60
                    *********:***.:*.********************:******* :*** **   ****

Deide_15980         MEAIAADLMGSPADPQSLPQQEKARNMEEAVAQSIEGVRQAIWLVSSKTSQEDAAAYQQM 120
revDR_0869          MEAMAAELLGTPPDPKDLPQQ-NTRDMEEVKAQSIEGVRQAAWLVSSKASPEDAAAYRQM 119
revDgeo_0511        LEAMAADLLGTPPDPKMLAGQERPRNLEEARAQSLEGVRQAVWLVDSKASPEDAAAYRRL 120
                    :**:**:*:*:*.**: *. * ..*::**. ***:****** ***.**:* ******:::

Deide_15980         LIEVVERTAQAAKEGGFLGIGGEQVNDKERQVIEELRRVIGGASVQGDSNTVTPTQETLP 180
revDR_0869          LLKVVENTANAAKEGGFLGIGGEQVNDKERAVMDELRQVIGGD----QTNQPTVVQETLP 175
revDgeo_0511        LWTVAERTAGAAKEGGFLGLGGEQVSEKERAVLDELRGVLGLEG-----GTAVLTHETPP 175
                    *  *.*.** *********:*****.:*** *::*** *:*        .  . .:** *

Deide_15980         ST-----PSPDGSGNSN 192
revDR_0869          ESTGTTMPAPDGSGNSN 192
revDgeo_0511        AG-------PGDSENGG 185
                             *..* *..
```

C

```
revDeide_15980      -----------------------------------------------------CATDHPP 7
DR_0869             MAQRWRSKRQSGPLAPCLLSLPSVAVAAAVGRWHRSPGGLRQGFLHHGRLVRLVAANHLT 60
Dgeo_0511           ---------------------------------------MRE------DRCSPLQTKHAA 15
                                                                       :.* .

revDeide_15980      QFLDDLALLIIDLLAAYAQEAPFLRCLRCTFHNLDQHLLVCGGVFLAGFTADQPDCLTHS 67
DR_0869             QFVHHGAFLVVDLFAADAQEAAFLRGVRGVFHHLEQHLAVSGGVFGARLAAHQPRGLPHA 120
Dgeo_0511           QFIQHGTFLLTHLLAAKAQKATLLGGPGSALGHGPKQAAVGGGILWAGLAVHQPDGLPHA 75
                    **:.. ::*: .*:** **:*.:*     .: :  ::  * **:: * ::..**  *.*:

revDeide_15980      LNALGHGFFHIAGLFLLWQRLRICRRAHQVSCDSFHQGGAACPADVLTHAGGNSLRFSQQ 127
DR_0869             LNALRFDLFHVAG-VLLGQVLGVGRGAEQFGGHRLHQRRARRAADTFAHGSGNRLRFGEQ 179
Dgeo_0511           LQALRAGFFQVAGPFLPCQHLGVGRRTQQIGGHRLQQRRSTLSAHLLANRLRDGLRLRHQ 135
                    *:**  .:*::** .*  * * : * :.*.. . ::*  :  .*. :::   : **: .*

revDeide_15980      AGQAAGAGCHDGGSRPARTGHDFEPLVCREVVFYAHKASLVT------------------ 169
DR_0869             PGQSAGAGGDHSGPRAAWPGHDLEPLVCREVVFYAHAASFNG------------------ 221
Dgeo_0511           ASQATGAGRHHRRACPAGTVHHREPLLRRKIVLDAHAITLNGRENPGLVGAFTGSESTSS 195
                    ..*::*** ..  . .* . *. ***: *::*: **  ::

revDeide_15980      ----GWPYAYWAFIYP---- 181
DR_0869             ----SCSFRRL--------- 228
Dgeo_0511           SISLSLPFPPLSRLTSTLRA 215
                        . .:
```
