# Supplementary material for: Alliance of Proteomics and Genomics to Unravel the Specificities of Sahara Bacterium Deinococcus deserti
Source: PLoS Genet. 2009 Mar 27;5(3):e1000434. doi: 10.1371/journal.pgen.1000434 (PMC2669436; doi:10.1371/journal.pgen.1000434)
Supplement: Figure S2 — Evidence for correct annotation of ddrC in Deinococcus species. Schematic representation of the ddrC loci and flanking genes in D. deserti, D. radiodurans and D. geothermalis (A). Multi-alignment of polypeptide sequences for correct DdrC proteins (B) and wrongly annotated DdrC proteins (Panel C). In Panel A, the predicted genes are labeled with the locus tag number. DR_0003 and Dgeo_0047 are incorrectly predicted, and their orientation has to be reversed. Correct ddrC genes are shown in black arrows, wrong ddrC ORFs in open arrows. The black boxes upstream the correct ddrC genes indicate the radiation response motif. Flanking genes Deide_23270 and Dgeo_0048 are homologs. (0.05 MB PDF) [file pgen.1000434.s002.pdf]

Figure S2

A

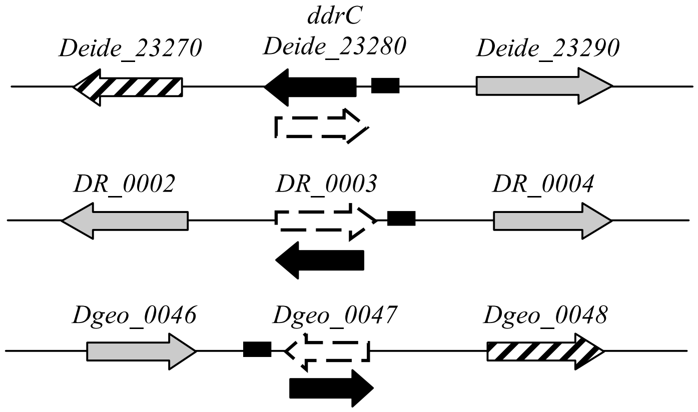

B

```
Deide_23280       MKSLPNTLDFGNVRLPVSADGLLHATTALAHLGLETPEQWDEFAHTHQLTSPLRDFGAGP 60
revDgeo_0047      MKTAPITLEFGTQRLPASADGLLHAATALKTLGVPLPEDWAAFAAAYDLRSPERDFGVGP 60
revDR_0003        MKNAPLTLNFGSVRLPVSADGLLHAPTAQQQLGLT--QSWEAALVEHGLPETYRDFGAGP 58
                  **. * **:**. ***.********.**   **:   :.*      : * .. ****.**

Deide_23280       EPTLHPDEFTRLAFVLDTIEARRWRKRAQTLLSRAMQGDVRLSAQIAERNPDPEARRWLA 120
revDgeo_0047      EATLSPDEFARLAFVVDTPEARRWRRRAQTLLARALTGDVRLAAQIAERNADPEARRWLA 120
revDR_0003        EAAVSVPDFVALAFALDTPEARRWQKRARELLARAMQGDVRVAAQIAERNPEPDARRWLA 118
                  *.::   :*. ***.:** *****::**: **:**: ****::*******.:*:******

Deide_23280       ARLESTHARRELLSTVSRHGGSGQVYGQLGSISNRSVLGTDSATIRRERGVKQTRDGLNS 180
revDgeo_0047      ARLESTGARRELISTVARHGGEGPVYGQLGSISNRSVLGTDSATIRRERAVRQTRDGLRT 180
revDR_0003        ARLESTGARRELLATVARHGGEGRVYGQLGSISNRTVLGKDSASVRQERGVKATRDGLTS 178
                  ****** *****::**:****.* ***********:***.***::*:**.*: ***** :

Deide_23280       TELLRMAYLDTATARAIQEHGAHGNAAILRVHEQVARRERLGWEMP-LTTQTG 232
revDgeo_0047      DELLRLAYLDTATARAISEQGVHGNAAILRLHEEVARRERSLWGGS-QAS--- 229
revDR_0003        AELLRLAYIDTVTARAIQESEARGNAAILTLHEQVARSERQSWERAGQVQRVG 231
                   ****:**:**.*****.*  .:****** :**:*** **  *  .  .
```

C

```
revDeide_23280     ----------------------------------------------------LGGQWHLP 8
DR_0003            MGTGDPSPLASQGPLPLVEGQKMKKPPPVRRGMNEAMEDRGSFFMALVSAYALHLARPFP 60
Dgeo_0047          ------------------------------------------------------------

revDeide_23280     AEAFAAGHLLVHAQNRSVAMRAVFLNGAGGGSVQVGHAQQFGAVQAVAGLLDPTLAADGR 68
DR_0003            ALALAAGYLFVEGEDGCVAPRFAFLNGAGGHGVDVGQPQQFGARQAVAGGLDAAFLTYRR 120
Dgeo_0047          ----------MQPQDRGIAVHPLLADGAGGGGIQIGQAQQFIGPQAVAGLPHSALAPNGG 50
                             :. ::  :* :  : :**** .:::*:.*** . *****  ..:: .

revDeide_23280     AVRAQDAAVADAAQLTVNLSGTAVTRDGAEQFTPGMGAFQAGGQPAPGFGVGVALGYLGA 128
DR_0003            AVLAKDGAVADAAELTVDPALATVPGDGGEQFAPGASAFQSGSEPAPGVRLGVALGNLGS 180
Dgeo_0047          GVGAQHAAVADAAQLSVHRPLAAVPRNGTYQLAAGAGAFQTGGQPAPRFRIRVAFCDLCG 110
                   .* *:..******:*:*. . ::*. :*  *::.* .***:*.:*** . : **:  * .

revDeide_23280     QAHVALHGARQQGLSALAPASGLNGVEDKGQTGELVGVQGGLGTGPEVAQGAGELMGVGK 188
DR_0003            HAHVALHGAGEQLPRPLLPAPGFGGVEGEGKRHEIGHAHGGLGSGAEVAVRLGQAVFDQR 240
Dgeo_0047          QAHITGQRTRQQRLRSPAPAPGFGRVDHKGEPRELIRRERRFWPHAKVALGAAQVVRCGK 170
                   :**:: : : :*   .  **.*:. *: :*:  *:   .  : . .:**   .: :   :

revDeide_23280     LVPLFGSFEAQMGQGGRGVQQAVRTDGQPDVAKVKGVRKALHT---------- 231
DR_0003            RFPALG--EPELLLGGGGVKQTVRADRQPHGAEIEGQRSVLHTDILAQIMFLT 291
Dgeo_0047          GGPVLRQGHAQSLQGSRGMQQTIRAGGQPLRAEFKRDGRGLHAYILAYFTLKT 223
                     * :   ..:   *. *::*::*:. **  *:.:     **:
```
