# Supplementary figures and images for: Alliance of Proteomics and Genomics to Unravel the Specificities of Sahara Bacterium Deinococcus deserti
Source: PLoS Genet. 2009 Mar 27;5(3):e1000434. doi: 10.1371/journal.pgen.1000434 (PMC2669436; doi:10.1371/journal.pgen.1000434)

**Figure S3**

**A**

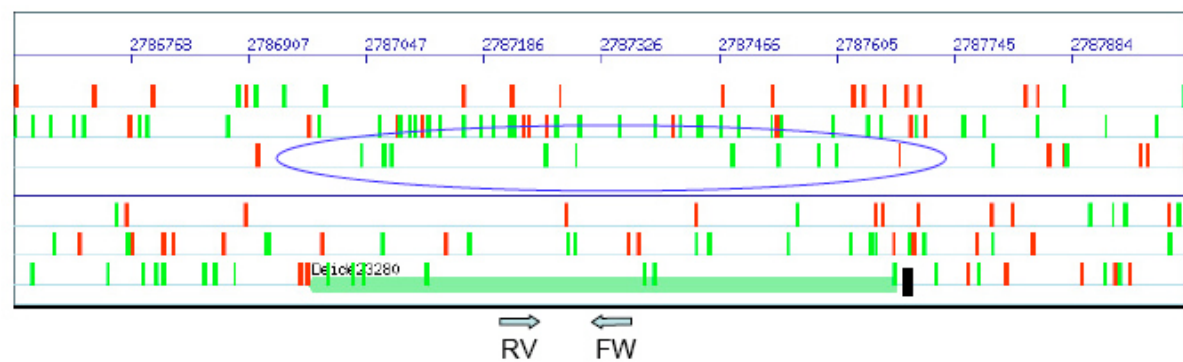

**B**

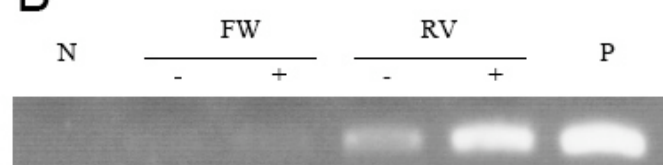

Supplement: Figure S3 — Specific RT-PCR showing transcription of correct ddrC (Deide_23280). Genome region of Deide_23280 (A). Deide_23280 is present on the reverse DNA strand. The opposite DNA strand could encode a protein related to DR_0003 and Dgeo_0047 (indicated by the blue ellipse). Green and red bar indicate start and stop codons, respectively. Black bar indicates the radiation/desiccation response motif upstream Deide_23280. RV and FW indicate schematically the orientation of the primers used in the RT-PCR. Specific two-step RT-PCR (B). Either purified primer FW (Dd23280FW) or RV (Dd23280RV) was used to synthesize cDNA in the RT reaction (first step). The PCR in the second step was performed with both primers. When Deide_23280 is the correctly predicted gene, cDNA synthesis and thus an RT-PCR product is expected with primer RV in the RT reaction. RNA was isolated 30 min after the cells were irradiated (+) or not (−) with UV (250 J/m2). N and P indicated negative (no template) and positive (genomic DNA) control, respectively. (0.11 MB PDF) [file pgen.1000434.s003.pdf]

Figure S5

A

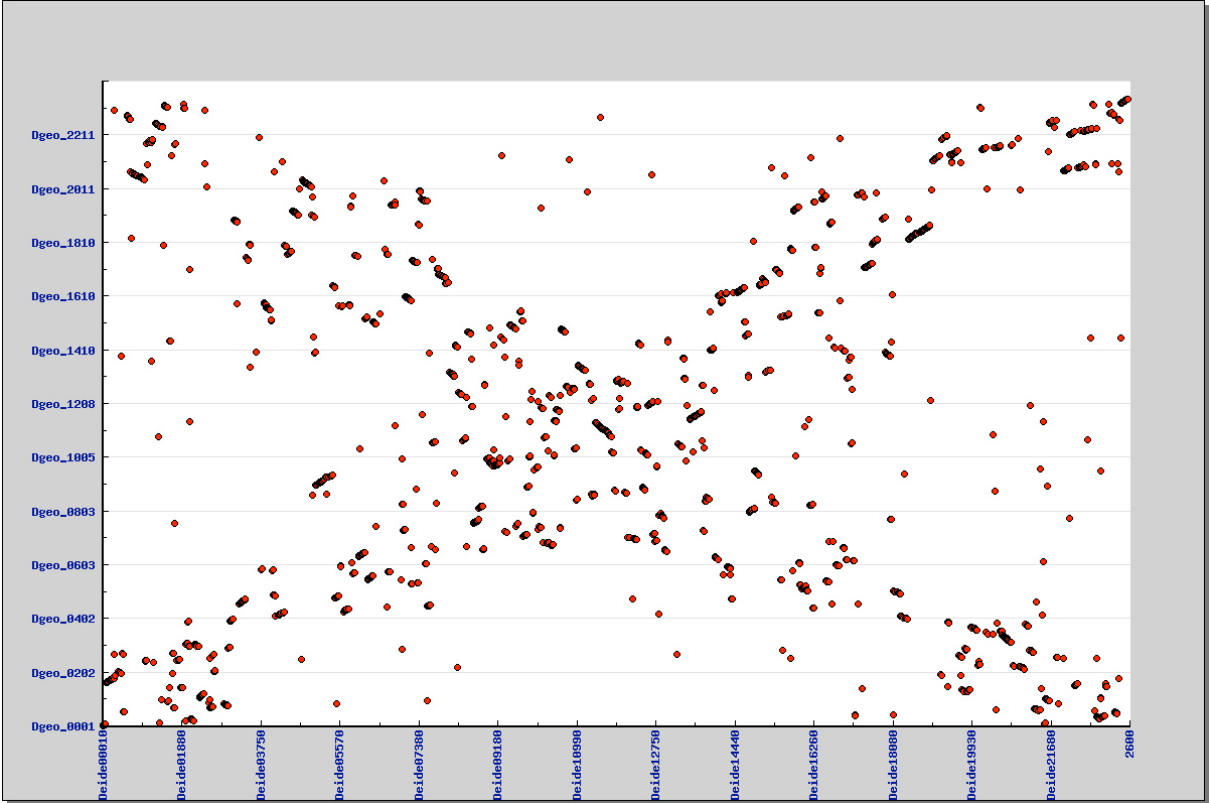

B

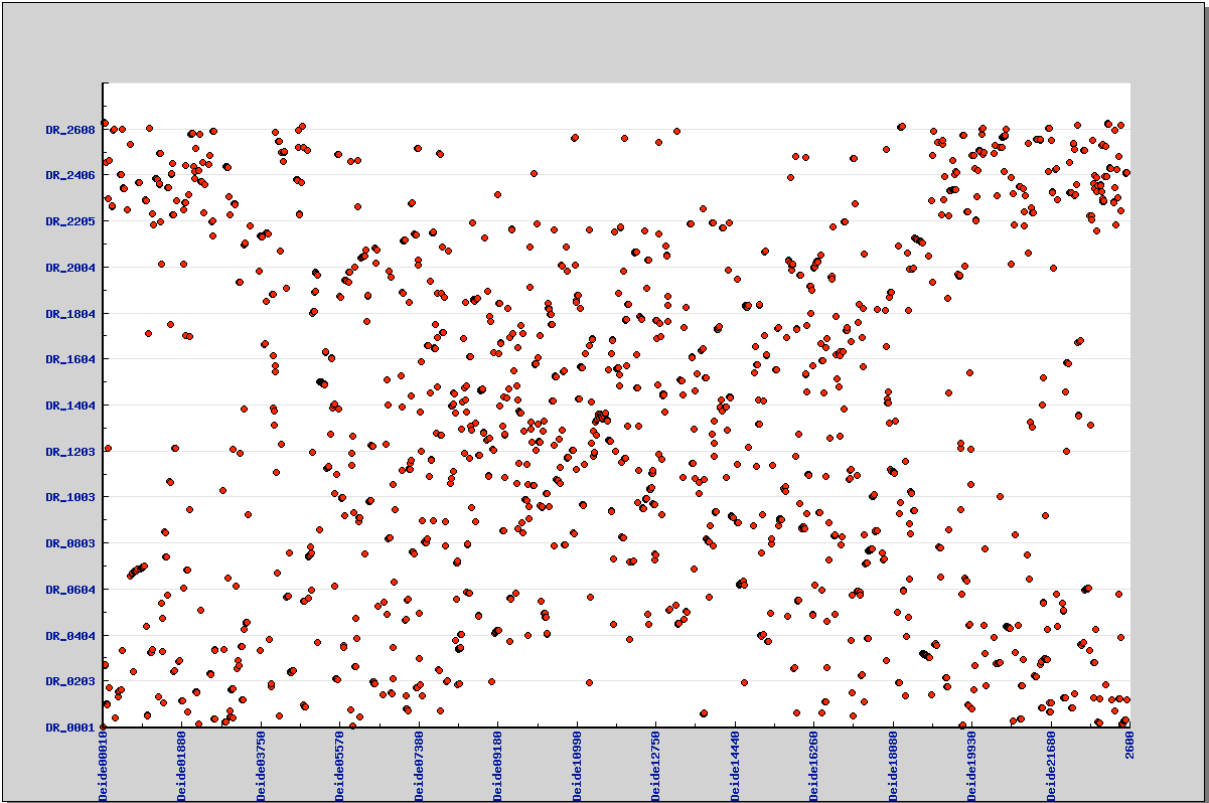

Supplement: Figure S5 — Genome dot plots for the chromosomes of sequenced Deinococcus species. Genome dot plots for the chromosome of D. deserti (horizontal axis) vs. D. geothermalis (vertical axis) (A) and vs. D. radiodurans (vertical axis) (B). Each dot represents the location of a pair of Bidirectional Best Hits (with a minimum of 30% identity and 70% coverage) between the two chromosomes. (0.22 MB PDF) [file pgen.1000434.s005.pdf]
