# Supplementary material for: Alliance of Proteomics and Genomics to Unravel the Specificities of Sahara Bacterium Deinococcus deserti
Source: PLoS Genet. 2009 Mar 27;5(3):e1000434. doi: 10.1371/journal.pgen.1000434 (PMC2669436; doi:10.1371/journal.pgen.1000434)
Supplement: Figure S4 — Evidence for correct annotation of ddrH. Multi-alignment of correct DdrH protein sequences (A) and wrongly annotated DdrH protein sequences (B). The D. radiodurans protein highly homologous to Deide_20641 and Dgeo_0322 is found when the orientation of DR_0438 (ddrH) is reversed. (0.03 MB PDF) [file pgen.1000434.s004.pdf]

Figure S4

A

```
Deide_20641     ----------MTNPYAEWFEQLRKEYGEQLGSMPLPEGLPEHLRALIDQHDEEAIQFMIK 50
Dgeo_0322       ----------MTNPYAEWFEQLRAEYGEQLKAMPLPDGLPEYLRDLIEARDEEAILFMIK 50
revDR_0438      MPKPSVSSQPAPNPYAEWFEQLRSEYGDQLSAMPLPDGLPEHLRNLIEQGDEDAIQFMVR 60
                           .*********** ***:** :****:****:** **:  **:** **::

Deide_20641     LAWQFGAQVGYAAGSRQNAAPAAYPRPGRVQA 82
Dgeo_0322       LAWQFGAQVGYAAGARQGQAARPTTRPGHVQA 82
revDR_0438      LAWQLGAQVGYAAGSRRQDVHAPPKRSGNVQA 92
                ****:*********:*:  .  .  *.*.***
```

B

```
DR_0438            ---------MHVLA----------AAAGGVPDLGAELPGEPDHKLNGVLVALLDQVSQVL 41
revDgeo_0322       VGAVRLRSSLDVTRPGRGSGRLSLSGSGGVTHLGPELPGKFDHKQDGFFVPRLDQVAQIL 60
revDeide_20641     -----VGSGLNAARPWVSRRCRILSASGGIAHLRAKLPGQFDHELNSFFVMLINQGPQMF 55
                            :..            :.:**:..* .:***: **: :..:*  ::* .*::

DR_0438            GQTVGQGHGAELVAVFAAELLEPLGVRVRRGLARDGRLRHGLNLTQAHSRNVSQKAV 98
revDgeo_0322       REAVGKRHGLELLAVLRAELLEPLGVGIR----------HTSILTPTHSRNVSGAKG 107
revDeide_20641     WQPFGQWHGPELLTILLAKLLEPLGIGVR----------HVSYLNITALKECNSGS- 101
                    :..*: ** **:::: *:******: :*          *   *. :  :: .
```
