# Supplementary material for: Alliance of Proteomics and Genomics to Unravel the Specificities of Sahara Bacterium Deinococcus deserti
Source: PLoS Genet. 2009 Mar 27;5(3):e1000434. doi: 10.1371/journal.pgen.1000434 (PMC2669436; doi:10.1371/journal.pgen.1000434)
Supplement: Table S1 — Insertion sequences identified in the genome of D. deserti and comparison with other Deinococcus/Thermus species. (0.09 MB PDF) [file pgen.1000434.s006.pdf]

Table S1. Insertion sequences identified in the genome of *D. deserti* and comparison with other *Deinococcus*/*Thermus* species

A. IS elements identified in the genome of *D. deserti*

**name**	**family (subgroup)**	**chromosome**		**plasmid 1**		**plasmid 2**		**plasmid 3**		**total genome**	
		**complete**	**partial**	**complete**	**partial**	**complete**	**partial**	**complete**	**partial**	**complete**	**partial**
IS*Dds1*	IS*3* (IS*3*)	2		1		2				5	
IS*Dds2*	IS*4* (IS*10*)	1			1					1	1
IS*Dds3*	IS*4* (IS*10*)			1	2				1	1	3
IS*Dds4*	IS*982*				1			1		1	1
IS*Dds5*	IS*982*							1		1	
IS*Dds6*	IS*701*							1		1	
IS*Dds7*	IS*4* (IS*10*)	1	1							1	1
IS*Dds8*	IS*630*	1								1	
IS*Dds9*	IS*5* (IS*427*)			1						1	
-	IS*5* (IS*1031*)		1		4		1		1		7
-	IS*5* (IS*427*)						1				1
-	IS*5* (IS*L2*)		1								1
-	IS*6*								1		1
-	IS*30*		1								1
-	IS*200*/IS*605* (ORFB)								1		1
-	IS*630*								3		3
-	IS*701*				1						1
-	IS*982*		1				1		1		3
**total**		**5**	**5**	**3**	**9**	**2**	**3**	**3**	**8**	**13**	**25**

**B. Comparison of IS family copy number and composition in three deinococci and *T. thermophilus***										
	D. deserti		***D. radiodurans***		***D. geothermalis***		***T. thermophilus* HB27**		***T. thermophilus* HB8**	
**IS family**	**complete**	**partial**	**complete**	**partial**	**complete**	**partial**	**complete**	**partial**	**complete**	**partial**
IS*1*					19	1				
IS*1595*										
IS*3*	5					4	1	1		
IS*481*				1						
IS*4*	3	5	21	17	6	7	1	2		
IS*H3*										
IS*701*	1	1			17	6	2	5		3
IS*1634*							3	1		
IS*5*	1	9	2		10	3	6	4	7	3
IS*1182*										
IS*6*		1			8	6				
IS*21*										
IS*30*		1								
IS*256*							1	1	8	
IS*630*	1	3	11		1		4	3	2	2
IS*982*	2	4			1	1				
IS*As1*										
IS*66*										
IS*110*							2	48	3	59
IS*91*										
IS*200*/IS*605*		1	10	1	4	4				1
IS*607*										
IS*1380*										
IS*L3*										
IS*NCY* subgroup IS*Bst12*			1		6	4				
Tn*3*			1			3				
**Total**	**13**	**25**	**46**	**19**	**72**	**39**	**20**	**65**	**20**	**68**
%	0.41	0.30	1.89	0.44	2.23	0.75	0.92	0.56	1.04	0.32
