# Supplementary material for: Alliance of Proteomics and Genomics to Unravel the Specificities of Sahara Bacterium Deinococcus deserti
Source: PLoS Genet. 2009 Mar 27;5(3):e1000434. doi: 10.1371/journal.pgen.1000434 (PMC2669436; doi:10.1371/journal.pgen.1000434)
Supplement: Table S4 — Homology between the DNA molecules of D. deserti, D. radiodurans and D. geothermalis. (0.07 MB PDF) [file pgen.1000434.s009.pdf]

Table S4. Homology between the DNA molecules of *D. deserti*, *D. radiodurans* and *D. geothermalis*

A. Homology between *D. deserti* and *D. geothermalis* [a,b]

D. deserti	*D. geothermalis* Chromosome	*D. geothermalis* Plasmid 1	*D. geothermalis* Plasmid 2
Chromosome	1804 (1648.3)	104 (244.2)	15 (30.5)
Plasmid P1	49 (85.7)	41 (12.7)	10 (1.6)
Plasmid P2	67 (146.6)	98 (21.7)	6 (2.7)
Plasmid P3	78 (117.4)	53 (17.4)	6 (2.2)

B. Homology between *D. deserti* and *D. radiodurans* [a,b]

D. deserti	*D. radiodurans* Chromosome 1	*D. radiodurans* Chromosome 2	*D. radiodurans* Megaplasmid	*D. radiodurans* Plasmid
Chromosome	1686 (1587.5)	105 (177.2)	29 (54.5)	1 (1.7)
Plasmid P1	49 (66.3)	18 (7.4)	8 (2.3)	1 (0.07)
Plasmid P2	70 (128.2)	60 (14.3)	17 (4.4)	0 (0.13)
Plasmid P3	58 (81.1)	25 (9.1)	10 (2.8)	0 (0.09)

[a] The observed number of orthologous proteins encoded on the different DNA molecules of *D. deserti vs. D. geothermalis* (A) and *vs. D. radiodurans* (B).

[b] In brackets, expected values in case orthologous relationships are independent of genome location.
