# Supplementary material for: Alliance of Proteomics and Genomics to Unravel the Specificities of Sahara Bacterium Deinococcus deserti
Source: PLoS Genet. 2009 Mar 27;5(3):e1000434. doi: 10.1371/journal.pgen.1000434 (PMC2669436; doi:10.1371/journal.pgen.1000434)
Supplement: Table S5 — Deinococcus-specific proteins. (0.16 MB PDF) [file pgen.1000434.s010.pdf]

**Table S5.** ***Deinococcus*****-specific proteins**[a]				
	D. deserti	***D. radiodurans***	***D. geothermalis***	**Comments**[b]
1	Deide_00230 (701 aa)	DR_2271 (695 aa)	Dgeo_0178 (702 aa)	membrane protein
2	Deide_00290 (198 aa)	DR_0041 (192 aa)	Dgeo_0188 (201 aa)	
3	Deide_00510 (549 aa)	DR_2348 (559 aa)	Dgeo_0055 (529 aa)	membrane protein
4	Deide_00530 (244 aa)	DR_2347 (332 aa)	Dgeo_0056 (236 aa)	roadblock/LC7 domain
5	Deide_00710 (156 aa)	DR_0673 (195 aa)<br>DR_0672 (169 aa)	Dgeo_2068 (154 aa)	signal peptide
6	Deide_01040 (100 aa)	DR_2297 (98 aa)	Dgeo_0243 (95 aa)	
7	Deide_01060 (129 aa)	DR_2295 (137 aa)	Dgeo_0245 (131 aa)	
8	Deide_01100 (166 aa)	DR_0050 (173 aa)	Dgeo_2100 (161 aa)	
9	Deide_01160 (158 aa)	DR_0326 (198 aa)	Dgeo_2186 (160 aa)	DdrD, radiation/desiccation-induced[c]
10	Deide_01210 (126 aa)	DR_2237 (112 aa)	Dgeo_2192 (123 aa)	
11	Deide_01441 (108 aa)	DR_0850 (105 aa)	Dgeo_2321 (98 aa)	
12	Deide_01450 (148 aa)	DR_0849 (177 aa)	Dgeo_2320 (145 aa)	
13	Deide_01520 (60 aa)	Upstream DR_2353 (60-130 aa)	Upstream Dgeo_0094 (62 aa)	membrane protein
14	Deide_01620 (535 aa)	DR_2412 (579 aa)<br>DR_A0207 (546 aa)	Dgeo_2134 (536 aa)	signal peptide
15	Deide_01640 (103 aa)	DR_2458 (89 aa)	Dgeo_0197 (89 aa)	membrane protein
16	Deide_01660 (265 aa)	DR_2232 (276 aa)	Dgeo_0273 (263 aa)	membrane protein
17	Deide_01720 (147 aa)	DR_1216 (158 aa)	Dgeo_2178 (150 aa)	signal peptide
18	Deide_01920 (88 aa)	DR_2016 (79 aa)	Dgeo_2311 (62 aa)	signal peptide
19	Deide_02000 (756 aa)	DR_0685 (713 aa)<br>DR_B0037 (691 aa)	Dgeo_0309 (762 aa)	signal peptide
20	Deide_02350 (107 aa)	DR_2378 (93 aa)	Dgeo_0113 (101 aa)	
21	Deide_02370 (164 aa)	DR_2527 (188 aa)	Dgeo_0115 (158 aa)	signal peptide
22	Deide_02380 (106 aa)	Downstream DR_2376 (> 42 aa ?)	Dgeo_0116 (94 aa)	
23	Deide_02660 (458 aa)	DR_2599 (481 aa)	Dgeo_0265 (457 aa)	signal peptide
24	Deide_02720 (71 aa)	DR_0338 (128 aa)	Dgeo_0209 (129 aa)	radiation/desiccation-induced[c]
25	Deide_02990 (178 aa)	DR_0070 (199 aa)	Dgeo_0295 (178 aa)	DdrB, radiation/desiccation-induced[c]
26	Deide_03030 (281 aa)	DR_0167 (328 aa)	Dgeo_0395 (289 aa)	DNA repair regulator protein IrrE/PprI
27	Deide_03060 (169 aa)	DR_1212 (168 aa)	Dgeo_0399 (155 aa)	signal peptide
28	Deide_03070 (103 aa)	DR_0040 (158 aa)	Dgeo_0400 (104 aa)	
29	Deide_03100 (135 aa)	DR_2281 (164 aa)	Dgeo_1892 (176 aa)	dsRNA binding motif
30	Deide_03200 (56 aa)	Downstream DR_1936 (56 aa)	Dgeo_0456 (56 aa)	membrane protein
31	Deide_03260 (102 aa)	DR_0355 (102 aa)	Dgeo_0462 (102 aa)	
32	Deide_03380 (700 aa)	DR_0458 (839 aa)	Dgeo_1752 (636 aa)	Pro+Gly-rich protein
33	Deide_03710 (194 aa)	DR_1987 (210 aa)	Dgeo_2200 (181 aa)	membrane protein

34	Deide_03750 (206 aa)	DR_2136 (206 aa)	Dgeo_0585 (217 aa)	
35	Deide_04010 (287 aa)	DR_1887 (252 aa)	Dgeo_0583 (275 aa)	membrane protein
36	Deide_04070 (250 aa)	DR_1575 (215 aa)	Dgeo_0487 (245 aa)	
37	Deide_04150 (219 aa)	DR_2554 (222 aa)	Dgeo_0415 (213aa)	membrane protein
38	Deide_04171 (66 aa)	DR_0047 (65 aa)	Dgeo_0418 (63 aa)	
39	Deide_04200 (262 aa)	DR_2504 (274 aa)	Dgeo_0421 (260 aa)	Zn-finger?
40	Deide_04350 (390 aa)	DR_0571 (397 aa)	Dgeo_1766 (386aa)	
41	Deide_04380 (358 aa)	DR_0574 (370 aa)	Dgeo_1769 (370 aa)	signal peptide
43	Deide_04431 (136 aa)	DR_0239 (134 aa)	Dgeo_1778 (116 aa)	
44	Deide_04602 (802 aa)	DR_2528 (503 aa)	Dgeo_1912 (709 aa)	
45	Deide_04830 (71 aa)	DR_0746 (79 aa)	Dgeo_2024 (72 aa)	
46	Deide_04970 (88 aa)	DR_1896 (94 aa)	Dgeo_1399 (95 aa)	
47	Deide_05301 (281 aa)	DR_1138 (254 aa)	Dgeo_0932 (189 aa)	signal peptide
48	Deide_05360 (150 aa)	DR_1607 (144 aa)	Dgeo_0935 (134 aa)	
49	Deide_05400 (181 aa)	DR_1405 + DR_1404<br>(107 aa + 73 aa, Frameshift)	Dgeo_1646 (178 aa)	signal peptide
51	Deide_05410 (153 aa)	DR_1406 (157 aa)	Dgeo_1645 (280 aa)	signal peptide ?
52	Deide_05420 (325 aa)	DR_1407 (365 aa)	Dgeo_1644 (311 aa)	signal peptide
53	Deide_05471 (99aa)	DR_0212 (101aa)	Dgeo_0482 (102aa)	
54	Deide_06160 (163 aa)	DR_2077 (169 aa)	Dgeo_1527 (165 aa)	
55	Deide_06180 (182 aa)	DR_1768 (134 aa)	Dgeo_1530 (188 aa)	signal peptide
56	Deide_06300 (344 aa)	DR_1226 (363 aa)	Dgeo_0558 (341 aa)	
57	Deide_06690 (173 aa)	DR_0150 (173 aa)	Dgeo_1950 (190 aa)	
58	Deide_06760 (98 aa)	DR_0634 (98 aa)	Dgeo_1954 (98 aa)	membrane protein
59	Deide_07131 (118 aa)	DR_1125 (115 aa)	Dgeo_1598 (110 aa)	membrane protein
60	Deide_07430 (183 aa)	DR_1590 (183 aa)	Dgeo_1972 (183aa)	
61	Deide_07560 (51 aa)	Between DR_1662/DR_1663<br>(46 aa)	Upstream Dgeo_0448 (50 aa)	
62	Deide_07850 (170 aa)	DR_0072 (179 aa)	Dgeo_1688 (146 aa)	
63	Deide_07860 (105 aa)	DR_1272 (102 aa)	Dgeo_1687 (108aa)	membrane protein
64	Deide_08155 (67 aa)	DR_1455 (98 aa)	Dgeo_1311 (67 aa)	possible regulator, HTH_MerR-trunc
65	Deide_08161 (248 aa)	DR_1454 (238 aa)	Dgeo_1310 (239 aa)	
66	Deide_08270 (347 aa)	DR_0343 (340 aa)	Dgeo_1250 (345 aa)	membrane protein
67	Deide_08510 (200 aa)	DR_1172 (298 aa)	Dgeo_1473 (236 aa)	desiccation-associated
68	Deide_08520 (122 aa)	DR_1615 (124 aa)	Dgeo_1472 (125 aa)	
69	Deide_08640 (123 aa)	DR_0895 (128 aa)	Dgeo_0762 (141 aa)	membrane protein
70	Deide_08680 (129 aa)	DR_1867 (120 aa)	Dgeo_0767 (119 aa)	membrane protein
71	Deide_08750 (156 aa)	DR_1465 (188 aa)	Dgeo_0817 (158 aa)	membrane protein, radiation/desiccation-induced[c]
72	Deide_08780 (456 aa)	DR_1469 (477 aa)	Dgeo_0820 (461 aa)	signal peptide, cell wall hydrolase/autolysin

73	Deide_08790 (180 aa)	DR_1470 (179 aa)	Dgeo_0821 (179 aa)	signal peptide
74	Deide_08920 (116 aa)	DR_1254 (114 aa)	Dgeo_1000 (109 aa)	membrane protein
75	Deide_09220 (87 aa)	DR_1847 (90 aa)	Dgeo_0982 (91 aa)	membrane protein
76	Deide_09250 (334 aa)	DR_1673 (348 aa)	Dgeo_1457 (324 aa)	
77	Deide_09300 (329 aa)	DR_0857 (285 aa)	Dgeo_1450 (336 aa)	membrane protein
78	Deide_09320 (232 aa)	DR_0858 (231 aa)	Dgeo_1448(226 aa)	membrane protein
79	Deide_09460 (279 aa)	DR_1699 (262 aa)	Dgeo_0997 (266 aa)	signal peptide
80	Deide_09710 (166 aa)	DR_1372 (164 aa)	Dgeo_1551 (166 aa)	signal peptide, desiccation-associated
81	Deide_09711 (382 aa)	DR_1371 (313 aa)	Dgeo_1552 (325 aa)	signal peptide
82	Deide_09720 (225 aa)	DR_1370 (216 aa)	Dgeo_1553 (239 aa)	signal peptide, radiation/desiccation-induced[c]
83	Deide_09750 (105aa)	DR_0889 (116 aa)	Dgeo_1518 (116 aa)	
84	Deide_09950 (284 aa)	DR_2090 (553 aa)	Dgeo_1009 (276 aa)	
85	Deide_10100 (175 aa)	DR_1584 (152 aa)	Dgeo_2428 (259 aa)	
86	Deide_10190 (174 aa)	DR_1242 (180 aa)	Dgeo_0743 (173 aa)	signal peptide
87	Deide_10201 (254 aa)	DR_A0140 (272 aa)	Dgeo_0741 (272 aa)	
88	Deide_10332 (140 aa)	DR_0409 (139 aa)	Dgeo_0683 (154 aa)	
89	Deide_10362 (114 aa)	DR_1821 (126 aa)	Dgeo_1238 (120 aa)	signal peptide
90	Deide_10421 (100 aa)	DR_1421 (93 aa)	Dgeo_0677 (92 aa)	
91	Deide_10480 (291 aa)	DR_1527 + DR_1528<br>(133 aa + 141 aa, Frameshift)	Dgeo_1144 (282 aa)	tetratricopeptide repeat (TPR) domain
92	Deide_10710 (205 aa)	DR_0795 (211 aa)	Dgeo_1478 (206 aa)	
93	Deide_10940 (287 aa)	DR_2569 (270 aa)	Dgeo_1038 (288 aa)	signal peptide
94	Deide_11000 (507 aa)	DR_1179 (548 aa)	Dgeo_1011 (496 aa)	
95	Deide_11010 (156aa)	DR_1882 (142 aa)	Dgeo_1351 (160 aa)	
96	Deide_11030 (65 aa)	DR_1429 (67 aa)	Dgeo_1349 (57 aa)	
97	Deide_11101 (433 aa)	DR_0969 (428 aa)<br>DR_1923+DR_1924<br>(276 aa + 168 aa, Frameshift)	Dgeo_1339 (432 aa)	signal peptide
98	Deide_11270 (171 aa)	DR_1416 (210aa)	Dgeo_1220 (168 aa)	
99	Deide_11331 (317 aa)	DR_1195 (296 aa)	Dgeo_0865 (316 aa)	membrane protein
100	Deide_11471 (122 aa)	DR_1364 (155 aa)	Dgeo_1119 (122 aa)	signal peptide
101	Deide_11690 (189 aa)	DR_1249 (192 aa)	Dgeo_1098 (189 aa)	signal peptide
102	Deide_11730 (152 aa)	DR_1245 (165 aa)	Dgeo_1085 (153 aa)	
103	Deide_11790 (75 aa)	DR_0734 (95 aa)	Dgeo_1022 (74 aa)	
104	Deide_11810 (65 aa)	DR_2157 (67 aa)	Dgeo_0880 (67 aa)	
105	Deide_12030 (390 aa)	DR_1985 (424 aa)	Dgeo_1291 (387 aa)	signal peptide, distantly related to beta-lactamase
106	Deide_12320 (376 aa)	DR_1480 (526 aa)	Dgeo_1193 (375 aa)	signal peptide ?
107	Deide_12361 (127 aa)	DR_1788 (127 aa)	Dgeo_1431 (121 aa)	signal peptide
108	Deide_12380 (103 aa)	DR_1786 (102 aa)	Dgeo_1429 (103 aa)	

109	Deide_12410 (419 aa)	DR_1781 (437 aa)	Dgeo_1031 (430 aa)	
110	Deide_12461 (168 aa)	DR_2161 (177aa)	Dgeo_0883 (179 aa)	
111	Deide_12490 (106 aa)	DR_0993 (116 aa)	Dgeo_1020 (99 aa)	
112	Deide_12670 (93 aa)	DR_1104 (95 aa)	Dgeo_1213 (104 aa)	
113	Deide_12750 (200 aa)	DR_1770 (247 aa)	Dgeo_0693 (206 aa)	membrane protein
114	Deide_13140 (132 aa)	DR_0452 (132 aa)	Dgeo_1056 (132 aa)	
115	Deide_13260 (646 aa)	DR_1448 + DR_1447 (426 aa + 449 aa, Frameshift)	Dgeo_1379 (649 aa)	signal peptide
116	Deide_13261 (150 aa)	DR_1446 (142 aa)	Dgeo_1378 (149 aa)	signal peptide
117	Deide_13540 (133 aa)	DR_1464 (159 aa)	Dgeo_1161 (75 aa)	signal peptide
118	Deide_13590 (93 aa)	DR_1539 (110 aa)	Dgeo_1167 (89 aa)	
119	Deide_13760 (598 aa)	DR_0060 + DR_0061 (142 aa + 478 aa, Frameshift)	Dgeo_0727 (591 aa)	
120	Deide_13820 (136 aa)	Reverse DR_0818 (130 aa)	Dgeo_0854 (133 aa)	
121	Deide_13990 (273 aa)	DR_1178 (278 aa)	Dgeo_1258 (271 aa)	distantly related to Phenazine biosynthesis PhzC/PhzF protein
122	Deide_14021 (404 aa)	DR_0938 (395 aa)	Dgeo_0631 (395 aa)	sporulation related domain
123	Deide_14071 (167 aa)	DR_1744 (170 aa)	Dgeo_1611 (166 aa)	
124	Deide_14160 (142 aa)	DR_2172 (139 aa)	Dgeo_1591 (140 aa)	signal peptide
125	Deide_14240 (262 aa)	DR_1397 (262 aa)	Dgeo_1619 (262 aa)	related to N-formylglutamate amidohydrolase
126	Deide_14280 (281 aa)	DR_1293 (276 aa)	Dgeo_0595 (260 aa)	membrane protein
127	Deide_14300 (122 aa)	DR_1990 (129 aa)	Downstream Dgeo_0594 (123 aa)	signal peptide
128	Deide_14350 (95 aa)	DR_0920 (87 aa)	Dgeo_0477 (92 aa)	
129	Deide_14630 (76 aa)	DR_0637 (77aa)	Dgeo_1639 (77 aa)	
130	Deide_14640 (142 aa)	DR_0638 (142aa)	Dgeo_1640 (142 aa)	membrane protein
131	Deide_14700 (217 aa)	DR_1832 (211aa)	Dgeo_1464 (219 aa)	
132	Deide_14710 (281 aa)	DR_1831 (291aa)	Dgeo_1465 (279 aa)	
133	Deide_14720 (134 aa)	DR_1830 (139 aa)	Dgeo_1466 (140 aa)	
134	Deide_14730 (552 aa)	DR_1829 (533 aa)	Dgeo_1467 (537 aa)	membrane protein
135	Deide_14870 (190 aa)	DR_0877 (114 aa)	Dgeo_1814 (186 aa)	
136	Deide_14981 (230 aa)	DR_1425 (206 aa)	Dgeo_0940 (231 aa)	signal peptide
137	Deide_14990 (168 aa)	DR_1320 (176 aa)	Dgeo_0939 (173 aa)	signal peptide
138	Deide_15020 (68 aa)	DR_1840 (79 aa)	Dgeo_1651 (83 aa)	
139	Deide_15100 (168 aa)	DR_1422 (190 aa)	Dgeo_1671 (167 aa)	radiation/desiccation-induced[c]
140	Deide_15270 (56 aa)	DR_0800 (60 aa)	Dgeo_2088 (68 aa)	radiation/desiccation-induced[c]
141	Deide_15290 (126 aa)	DR_1140 (124 aa)	Dgeo_0837 (127 aa)	signal peptide
142	Deide_15291 (138 aa)	DR_1139 (131 aa)	Dgeo_0836 (132 aa)	distantly related to inorganic pyrophosphatase
143	Deide_15380 (342 aa)	DR_1557 (343 aa)	Dgeo_1707 (351 aa)	
144	Deide_15470 (160 aa)	DR_0909 (160 aa)	Dgeo_1532 (171 aa)	membrane protein

145	Deide_15480 (708 aa)	DR_0904 + DR_0903<br>(369 aa + 518 aa, Frameshift)	Dgeo_0547 (656 aa)	signal peptide, vWA domain
146	Deide_15730 (104 aa)	DR_2020 (131 aa)	Dgeo_1783 (99 aa)	
147	Deide_15900 (244 aa)	DR_0554 (244 aa)	Dgeo_1941 (219 aa)	
148	Deide_15980 (192 aa)	Reverse DR_0869 (228 aa)	Reverse Dgeo_0511 (215 aa)	
149	Deide_16050 (238 aa)	DR_0864 (293 aa)	Dgeo_0520 (285 aa)	
150	Deide_16060 (254 aa)	DR_0863 (295 aa)	Dgeo_0521 (270 aa)	membrane protein
151	Deide_16110 (168 aa)	DR_1748 (162 aa)	Dgeo_0507 (152 aa)	
152	Deide_16251 (99 aa)	DR_2001 (103 aa)	Dgeo_0440 (100 aa)	
153	Deide_16260 (128 aa)	DR_2002 (129 aa)	Dgeo_1961 (127 aa)	
154	Deide_16270 (178 aa)	DR_2003 (210 aa)	Dgeo_1962 (166 aa)	signal peptide
155	Deide_16430 (93 aa)	DR_0600 (79 aa)	Dgeo_1997 (101 aa)	
156	Deide_16470 (316 aa)	DR_1597 (312 aa)	Dgeo_1975 (323 aa)	
157	Deide_16551 (287 aa)	DR_1693 (250 aa)	Dgeo_0542 (184 aa)	
158	Deide_16650 (73 aa)	Reverse DR_0371 (73 aa)	Dgeo_1881 (73 aa)	
159	Deide_16690 (165 aa)	DR_1962 (170 aa)	Dgeo_1885 (184 aa)	
160	Deide_16890 (121 aa)	DR_1269 (124 aa)	Dgeo_1592 (152 aa)	membrane protein
161	Deide_17000 (223 aa)	DR_1388 (217 aa)	Dgeo_0664 (211 aa)	signal peptide
162	Deide_17180 (123 aa)	DR_1121 (126 aa)	Dgeo_1382 (124 aa)	
163	Deide_17470 (93 aa)	DR_1697 (86 aa)	Dgeo_0142 (91 aa)	
164	Deide_17520 (244 aa)	DR_0714 (266 aa)	Dgeo_1717 (243 aa)	membrane protein
165	Deide_17591 (309 aa)	DR_0769 (267 aa)	Dgeo_1724 (258 aa)	signal peptide
166	Deide_17770 (625 aa)	DR_0855 (656 aa)	Dgeo_1817 (637 aa)	signal peptide
167	Deide_17940 (97 aa)	DR_1432 (89 aa)	Dgeo_1393 (96aa)	
168	Deide_17950 (382 aa)	DR_1414 (527 aa)	Dgeo_1392 (302 aa)	membrane protein, Ala+Pro-rich protein
169	Deide_17971 (53 aa)	DR_1463 (97 aa)	Downstream Dgeo_1389 (49 aa)	signal peptide ?
170	Deide_18050 (313 aa)	DR_1116 (341 aa)	Dgeo_1437 (300 aa)	
171	Deide_18130 (61 aa)	DR_1331 (81 aa)	Dgeo_0501 (57 aa)	
172	Deide_18240 (199 aa)	Reverse DR_0931 (Frameshift?)	Downstream Dgeo_0497 (199 aa)	membrane protein
173	Deide_18480 (108 aa)	DR_0887 (128 aa)	Dgeo_1823 (106 aa)	
174	Deide_18980 (144aa)	DR_0308 (147 aa)	Dgeo_1870 (138 aa)	membrane protein
175	Deide_19055 (72 aa)	DR_1936 (68 aa)	Dgeo_2113 (70 aa)	
176	Deide_19131 (131 aa)	DR_0359 (159 aa)	Dgeo_2126 (163 aa)	membrane protein
177	Deide_19231 (54 aa)	DR_2230 (75 aa)	Dgeo_0191 (90 aa)	
178	Deide_19320 (108aa)	DR_0218 (146 aa)	Dgeo_2204 (131 aa)	
179	Deide_19410 (184 aa)	DR_2229 (175 aa)	Dgeo_0384 (146 aa)	
180	Deide_19481 (237 aa)	DR_2342 (220 aa)	Dgeo_2142 (217 aa)	
181	Deide_19530 (149 aa)	DR_2344 (177 aa)	Dgeo_2146 (178 aa)	signal peptide
182	Deide_19650 (129 aa)	DR_0947 (116 aa)	Dgeo_0259 (143 aa)	candidate isochorismatase family protein

183	Deide_19741 (157 aa)	DR_2006 (157 aa)	Dgeo_0289 (163 aa)	
184	Deide_19870 (248 aa)	DR_0449 (243 aa)	Dgeo_0137 (246 aa)	
185	Deide_19890 (230 aa)	DR_1210 (225 aa)	Dgeo_0371 (241 aa)	
186	Deide_19972 (311aa)	DR_0269 (322 aa)	Dgeo_0364 (248 aa)	Gly-rich protein
187	Deide_20360 (195 aa)	DR_2500 (157 aa)	Dgeo_2163 (198 aa)	
188	Deide_20411 (83 aa)	DR_2314 (117 aa)	Dgeo_0383 (119 aa)	
189	Deide_20560 (551 aa)	DR_2572 + DR_2573<br>(496 aa + 103 aa, Frameshift)	Dgeo_0337 (501 aa)	membrane protein, radiation/desiccation-induced[c]
190	Deide_20570 (129 aa)<br>Deide_3p02170 (129 aa)	DR_2574 (131 aa)	Dgeo_0336 (140 aa)	DdrO, radiation/desiccation-induced[c]
191	Deide_20641 (82 aa)	Reverse DR_0438 (92 aa)	Dgeo_0322 (82 aa)	correct DdrH, radiation/desiccation-induced[c]
192	Deide_20645 (64 aa)	DR_0437 (72 aa)	Dgeo_0320 (65 aa)	
193	Deide_20690 (83 aa)	Upstream DR_0433 (84 aa)	Upstream Dgeo_0316 (82-105 aa)	
194	Deide_20710 (160 aa)	DR_2018 (193 aa)	Dgeo_0314 (149 aa)	membrane protein
195	Deide_20990 (97 aa)	DR_0296 (98 aa)	Dgeo_0218 (104 aa)	
196	Deide_21020 (213 aa)	DR_2183 (210 aa)	Dgeo_2478 (198 aa)	
197	Deide_21230 (445 aa)	DR_1308 (447 aa)	Dgeo_0277 (447 aa)	signal peptide, related to peptidases
198	Deide_21250 (142 aa)	DR_2240 (142 aa)	Dgeo_0275 (141 aa)	
199	Deide_21340 (73 aa)<br>Deide_2p01315 (63 aa)	DR_2563 (70 aa)	Dgeo_0465 (72 aa)	radiation/desiccation-induced[c]
200	Deide_21400 (79 aa)	DR_2559 (75 aa)	Dgeo_0061 (80 aa)	
201	Deide_21470 (162 aa)<br>Deide_3p01970 (158 aa)	DR_0082 (154 aa)<br>DR_2593 (185 aa)	Dgeo_0413 (115-166 aa)	Gly-rich protein
202	Deide_21480 (88 aa)	DR_0545 (95 aa)	Dgeo_0616 (90 aa)	membrane protein
203	Deide_21660 (301 aa)	DR_0067 (309 aa)	Dgeo_2257 (301 aa)	
204	Deide_21670 (121aa)	DR_0068 (124 aa)	Dgeo_2258 (124 aa)	membrane protein
205	Deide_22050 (238 aa)	DR_1202 (249 aa)	Dgeo_2082 (270aa)	signal peptide
206	Deide_22420 (99 aa)	DR_0360 (143 aa)	Dgeo_2093 (122 aa)	
207	Deide_22690 (171 aa)	DR_2558 (156 aa)	Dgeo_2325 (192 aa)	
208	Deide_22781 (295 aa)	DR_2159 (255 aa)	Dgeo_2233 (251 aa)	signal peptide
209	Deide_22790 (394aa)	DR_2334 (386 aa)	Dgeo_0035 (391 aa)	
210	Deide_22810 (95 aa)	DR_0021 (88 aa)	Dgeo_0033 (96 aa)	
211	Deide_22830 (289 aa)	DR_0019 (293 aa)	Dgeo_0031 (289 aa)	
212	Deide_22840 (219 aa)	DR_0018 (205 aa)	Dgeo_0030 (206 aa)	
213	Deide_22945 (89 aa)	DR_2292 (85 aa)	Dgeo_0039 (75 aa)	
214	Deide_23110 (385 aa)	DR_0121 (449 aa)	Dgeo_2293 (384 aa)	signal peptide, tetratricopeptide repeat (TPR) domain
215	Deide_23280 (232 aa)	Reverse DR_0003 (291 aa)	Reverse Dgeo_0047 (223 aa)	correct DdrC, radiation/desiccation-induced[c]
216	Deide_23300 (545 aa)	DR_2489 (477 aa)	Dgeo_0179 (579 aa)	
217	Deide_23320 (80 aa)	DR_0124 (94 aa)	Dgeo_2267 (78 aa)	

218	Deide_23410 (106 aa)	DR_0015 (118 aa)	Dgeo_2336 (108 aa)	
219	Deide_23460 (77 aa)	DR_0031 (80 aa)	Dgeo_2340 (77 aa)	membrane protein
220	Deide_23480 (137 aa)	DR_2414 (157 aa)	Dgeo_2342 (147 aa)	signal peptide, radiation/desiccation-induced[c]
221	Deide_1p00071 (440 aa)<br>Deide_3p02680 (429 aa)	DR_B0145 (397 aa)	Dgeo_2497 (467 aa)	related to plasmid replication initiator protein
222	Deide_1p01253 (160 aa)	DR_A0141 (144 aa)	Dgeo_2285 (151 aa)<br>Dgeo_2915 (163 aa)	
223	Deide_1p01530 (90 aa)	DR_0545 (90 aa)	Dgeo_0616 (90 aa)	membrane protein
224	Deide_1p01584 (590 aa)	DR_2489 (477 aa)	Dgeo_0179 (579 aa)	signal peptide
225	Deide_2p00020 (231 aa)	DR_2215 (260 aa)	Dgeo_2492 (237 aa)	
226	Deide_2p00100 (395 aa)	DR_A0217 (394 aa)	Dgeo_2412 (386 aa)	
227	Deide_2p00260 (521 aa)	DR_A0207 (546 aa)<br>DR_2412 (579 aa)	Dgeo_2134 (536 aa)	signal peptide
227	Deide_2p01100 (287 aa)	DR_A0182 (268 aa)	Dgeo_2942 (319 aa)	
228	Deide_2p01380 (294 aa)	DR_A0346 (300 aa)	Dgeo_2628 (302 aa)	PprA, radiation/desiccation-induced[c]
229	Deide_2p02110 (168 aa)	DR_A0252 (154 aa)	Dgeo_2407 (156 aa)	candidate transcriptional regulator, MER family
230	Deide_2p02131 (247 aa)	DR_0367 (266 aa)	Dgeo_2187 (243 aa)	

[a] *Deinococcus*-specific proteins have at least 30% identity and 70% coverage using Blastp analysis, whereas hits with proteins from other organisms, if present, do not fullfill these criteria. After manual inspection, several other *Deinococcus*-specific proteins were added, including those for which frameshifts were observed, those requiring reversion of gene orientation and non-predicted proteins. Proteins in grey were identified in the proteome analysis after standard cultivation.

[b] TMHMM was used to predict transmembrane α-helices, and SignalP, TatP and LipoP to predict signal peptides (1). A protein was considered a membrane protein when at least two transmembrane segments were predicted.

[c] Radiation and/or desiccation-induced gene in *D. radiodurans* (2)

1. Emanuelsson O, Brunak S, von Heijne G, Nielsen H (2007) Locating proteins in the cell using TargetP, SignalP and related tools. Nat Protoc 2: 953-971.
2. Tanaka M, Earl AM, Howell HA, Park MJ, Eisen JA, et al. (2004) Analysis of *Deinococcus radiodurans*'s transcriptional response to ionizing radiation and desiccation reveals novel proteins that contribute to extreme radioresistance. Genetics 168: 21-33.
