# Supplementary material for: Alliance of Proteomics and Genomics to Unravel the Specificities of Sahara Bacterium Deinococcus deserti
Source: PLoS Genet. 2009 Mar 27;5(3):e1000434. doi: 10.1371/journal.pgen.1000434 (PMC2669436; doi:10.1371/journal.pgen.1000434)
Supplement: Table S6 — Mn/Fe ratio in D. deserti and D. radiodurans. (0.08 MB PDF) [file pgen.1000434.s011.pdf]

Table S6. Mn/Fe ratio in *D. deserti* and *D. radiodurans*

	TSB/10		TSB/10 + trace elements[c]	
	D. deserti	*D. radiodurans*	*D. deserti*	*D. radiodurans*
Mn[a]	0.38	0.15	2.04	0.66
Fe[a]	0.7	0.89	1.9	1.43
Mn/Fe[b]	0.54	0.16	1.05	0.47

[a] In nmol/mg dry weight. Strains were grown in tenfold diluted tryptic soy broth (TSB/10) or TSB/10 supplemented with trace elements, washed in PBS containing 1 mM EDTA, and cells were dried at 60°C for 48 h, and subjected to ICP-AES.

[b] The Mn/Fe ratio in TSB/10 + trace elements is 0.19; Mn and Fe in TSB/10 were below the detection limit.

[c] Vujičić-Žagar A, Dulermo R, Le Gorrec M, Vannier F, Servant P, et al. (2009) Crystal structure of the IrrE protein, a central regulator of DNA damage repair in Deinococcaceae. J Mol Biol. In Press.
