# Supplementary material for: Alliance of Proteomics and Genomics to Unravel the Specificities of Sahara Bacterium Deinococcus deserti
Source: PLoS Genet. 2009 Mar 27;5(3):e1000434. doi: 10.1371/journal.pgen.1000434 (PMC2669436; doi:10.1371/journal.pgen.1000434)
Supplement: Table S7 — Mn- and Fe- transport and regulation related systems in D. deserti. (0.08 MB PDF) [file pgen.1000434.s012.pdf]

**Table S7. Mn- and Fe- transport and regulation related systems in *D. deserti***						
**A. Mn-transport and regulation related systems**						
**COG number**	**Protein annotation**	**Name**	***D. radiodurans* ortholog**	***D. geothermalis* ortholog**	***D. deserti* ortholog**	**Spectral count**
**COG1108**	**ABC-type Mn2+/Zn2+ transport system (permease component)**	**ZnuB**	**-**	**-**	**Deide_3p02750**	**0**
**COG0803**	**ABC-type Mn/Zn transport system (periplasmic component)**	**FimA/TroA**	**DR_2523**	**Dgeo_0534**	**Deide_07330**	**3**
**COG0803**	**ABC-type Mn/Zn transport system (periplasmic component)**	**FimA/TroA**	**-**	**-**	**Deide_3p02720**	**2**
**COG0803**	**ABC-type Mn/Zn transport system (periplasmic component)**	**FimA/TroA**	**-**	**-**	**Deide_2p01321**	**0**
**COG0803**	**ABC-type Mn/Zn transport system (periplasmic component)**	**FimA/TroA**	**-**	**-**	**Deide_1p00820**	**0**
**COG1108**	**ABC-type Mn2+/Zn2+ transport system (permease component)**	**ZnuB**	**DR_2283**	**Dgeo_0532**	**Deide_07190**	**0**
**COG1108**	**ABC-type Mn2+/Zn2+ transport system (permease component)**	**ZnuB**	**-**	**-**	**Deide_3p02740**	**0**
**COG1108**	**ABC-type Mn2+/Zn2+ transport system (permease component)**	**ZnuB**	**-**	**-**	**Deide_2p01340**	**0**
**COG1108**	**ABC-type Mn2+/Zn2+ transport system (permease component)**	**ZnuB**	**-**	**-**	**Deide_2p01350**	**0**
**COG1108**	**ABC-type Mn2+/Zn2+ transport system (permease component)**	**ZnuB**	**-**	**-**	**Deide_3p02750**	**0**
**COG1121**	**ABC-type Mn/Zn transport system (ATPase component)**	**ZnuC**	**DR_2284**	**Dgeo_0533**	**Deide_07200**	**0**
**COG1121**	**ABC-type Mn/Zn transport system (ATPase component)**	**ZnuC**	**-**	**-**	**Deide_3p02730**	**0**

**COG1121**	**ABC-type Mn/Zn transport system (ATPase component)**	**ZnuC**	**-**	**-**	**Deide_2p01330**	**2**
**COG1321**	**Mn-dependent transcriptional regulator**	**TroR/DtxR**	**DR_2539**	**Dgeo_2268**	**Deide_00680**	**0**
**COG1321**	**Mn-dependent transcriptional regulator**	**TroR/DtxR**	**-**	**-**	**Deide_3p02021**	**2**
**COG1321**	**Mn-dependent transcriptional regulator**	**TroR/DtxR**	**-**	**-**	**Deide_3p02751**	**0**
**COG1914**	**NRAMP family transporter**	**MntH**	**DR_1709**	**Dgeo_0709**	**Deide_3p02300**	**0**
**B. Fe-transport and regulation related systems**						
**COG number**	**Protein annotation**	**Name**	***D. radiodurans* ortholog**	***D. geothermalis* ortholog**	***D. deserti* ortholog**	**Spectral count**
**COG0370**	**Fe(II) transport system protein B**	**FeoB**	**DR_1219**	**Dgeo_0894**	**-**	**-**
**COG0609**	**ABC-type Fe(III)-siderophore transport system (permease component)**	**FepD**	**DR_2589**	**Dgeo_0024**	**Deide_02150**	**0**
**COG0609**	**ABC-type Fe(III)-siderophore transport system (permease component)**	**FepD**	**DR_B0015**	**Dgeo_0125**	**-**	**0**
**COG0609**	**ABC-type Fe(III)-siderophore transport system (permease component)**	**FepD**	**DR_B0122**	**Dgeo_2758**	**Deide_2p00390**	**0**
**COG0609**	**ABC-type Fe(III)-siderophore transport system (permease component)**	**FepD**	**DR_B0123**	**Dgeo_2756**	**Deide_2p00370**	**0**
**COG0609**	**ABC-type Fe(III)-siderophore transport system (permease component)**	**FepD**	**DR_C0007**	**-**	**-**	**-**
**COG0614**	**ABC-type Fe(III)-hydroxamate transport system (periplasmic component)**	**BtuF**	**DR_0462**	**-**	**-**	**-**

COG0614	ABC-type Fe(III)-hydroxamate transport system (periplasmic component)	BtuF	DR_1373	Dgeo_0657	Deide_12970	2
COG0614/COG4594	ABC-type Fe(III)-hydroxamate transport system (periplasmic component)	HutB (FhuD-like)/FecB	-	Dgeo_2757	Deide_2p00380	1
COG0614/COG4594	ABC-type Fe(III)-hydroxamate transport system (periplasmic component)	HutB (FhuD-like)	-	-	Deide_21930	0
COG0614/COG4594	ABC-type Fe(III)-hydroxamate transport system (periplasmic component)	HutB (FhuD-like)	-	Dgeo_2754	-	-
COG0614	ABC-type Fe(III)-hydroxamate transport system (periplasmic component)	FhuD	DR_B0125	Dgeo_2753	-	-
COG0614/COG4594	ABC-type Fe(III)-hydroxamate transport system (periplasmic component)	FhuD	-	Dgeo_1370	Deide_17140	5
COG0614	ABC-type Fe(III)-hydroxamate transport system (periplasmic component)	YvrC	DR_2588	Dgeo_0025	Deide_02140	122
COG0614	ABC-type Fe(III)-hydroxamate transport system (periplasmic component)	YvrC-like	DR_B0007	Dgeo_2363	Deide_2p01550	66
COG0735	Fe(II)/Zn(II) uptake regulation proteins	Fur (Zur)	DR_0865	Dgeo_0519	Deide_16041	0
COG0735	Fe(II)/Zn(II) uptake regulation proteins	Fur	Upstr DR_2342	Dgeo_2141	Deide_19480	0
COG0735	Fe(II)/Zn(II) uptake regulation proteins	Fur	-	Dgeo_2727	Deide_2p00340	0
COG0783	DNA-binding ferritin-like protein (oxidative damage protectant)	Dps	DR_2263	Dgeo_0281	Deide_21200	501
COG0783	DNA-binding ferritin-like protein (oxidative damage protectant)	Dps-like (Dps92)	DR_B0092	-	-	-

**COG1120**	**ABC-type cobalamin/Fe(III)-siderophore transport system (ATPase component)**	**FepC**	**DR_2590**	**Dgeo_0023**	**Deide_02160**	**0**
**COG1120**	**ABC-type cobalamin/Fe(III)-siderophore transport system (ATPase component)**	**FepC-like**	**DR_B0121**	**Dgeo_2759**	**Deide_2p00400**	**0**
**COG1178**	**ABC-type Fe3+ transport system (permease component)**	**ThiP**	**-**	**-**	**Deide_19080**	**0**
**COG1178**	**ABC-type Fe3+ transport system (permease component)**	**ThiP**	**DR_2488**	**Dgeo_1934**	**Deide_15850**	**0**
**COG1840**	**ABC-type Fe3+ transport system (periplasmic component)**	**AfuA**	**-**	**-**	**Deide_19090**	**129**
**COG1918**	**Fe(II) transport system protein A**	**FeoA**	**DR_1220**	**Dgeo_0895**	**-**	**-**
**COG2375**	**siderophore -interacting protein**	**ViuB**	**DR_B0017**	**Dgeo_0127**	**-**	**-**
**COG2375**	**siderophore -interacting protein**	**ViuB-like**	**DR_B0124**	**-**	**-**	**-**
**COG4264**	**Siderophore synthetase component**	**RhbC**	**-**	**-**	**Deide_21890**	**1**
**COG4264**	**Siderophore synthetase component**	**RhbC**	**-**	**-**	**Deide_21910**	**0**
**COG4559**	**ABC-type hemin transport system (ATPase component)**	**HmuV**	**DR_B0016**	**Dgeo_0126**	**-**	**-**
