# Supplementary material for: Alliance of Proteomics and Genomics to Unravel the Specificities of Sahara Bacterium Deinococcus deserti
Source: PLoS Genet. 2009 Mar 27;5(3):e1000434. doi: 10.1371/journal.pgen.1000434 (PMC2669436; doi:10.1371/journal.pgen.1000434)
Supplement: Table S8 — Stress response-related proteins in D. deserti, D. radiodurans and D. geothermalis. (0.16 MB PDF) [file pgen.1000434.s013.pdf]

Table S8. Stress response-related proteins in *D. deserti*, *D. radiodurans* and *D. geothermalis*				
Gene name[a]	*D. deserti*	*D. radiodurans*	*D. geothermalis*	Protein description
**General/heat**				
groL	Deide_22590 (549 aa)	DR_0607 (548 aa)	Dgeo_2231 (545 aa)	Hsp10, molecular chaperone
grpE	Deide_21980 (216 aa)	DR_0128 (221aa)	Dgeo_2077 (218 aa)	Hsp20, molecular chaperone
groS	Deide_22580 (95 aa)	DR_0606 (120 aa)	Dgeo_2230 (95 aa)	Hsp60, molecular chaperone
dnaK	Deide_21970 (628 aa)	DR_0129 (628 aa)	Dgeo_2076 (629 aa)	Hsp70, molecular chaperone
dnaJ	Deide_22000 (306 aa)<br>Deide_07590 (373 aa)	DR_0126 (313 aa)<br>DR_1424 (420 aa)	Dgeo_2078 (302 aa)<br>Dgeo_0451 (371 aa)	Hsp70, chaperone cofactor
ibpA/*ibpB*	Deide_18090 (164 aa)<br>Deide_11290 (136 aa)<br>Deide_1p01285 (129 aa)	DR_1114 (182 aa)<br>DR_1691 (166 aa)	Dgeo_0505 (158 aa)<br>Dgeo_0859 (134 aa)	Small heat shock protein
hslJ	Deide_07610 (221 aa)	DR_2056 (147 aa)<br>DR_1940 (403 aa)	-<br>-	Related to heat shock protein
htpG	-	-	-	Hsp90, molecular chaperone
dksA	-	-	-	DnaK suppressor protein
clpA/*clpB*/*clpC*	Deide_08490 (739 aa)<br>Deide_12640 (852 aa)<br>Deide_12680 (747 aa)	DR_0588 (741 aa)<br>DR_1046 (875 aa)<br>DR_1117 (747 aa)	Dgeo_1475 (737 aa)<br>Dgeo_1210 (861 aa)<br>Dgeo_1215 (745 aa)	ATPase subunits of Clp protease
clpX	Deide_19580 (401 aa)	DR_1973 (403 aa)	Dgeo_2152 (406 aa)	ATPase subunit of Clp protease
clpP	Deide_19570 (184 aa)	DR_1972 (204 aa)	Dgeo_2151 (200 aa)	ATP-dependent protease with chaperone activity
lon	Deide_19590 (808 aa)<br>Deide_05670 (820aa)	DR_1974 (813 aa)<br>DR_0349 (821 aa)	Dgeo_2153 (813 aa)<br>Dgeo_0427 (820 aa)	ATP-dependent Lon Serine protease
BS_*hslU* (*clpY)*	-	-	-	ATPase subunit of Clp photolytic system
BS_*hslV* (*clpQ)*	-	-	-	ATP-dependent protease HslV
sms (radA)	Deide_12660 (449 aa)	DR_1105 (503 aa)	Dgeo_121 (449 aa)	ATP-dependent serine protease COG1066 / DNA repair protein
htrA	Deide_21350 (391 aa)<br>Deide_01170 (389 aa)<br>Deide_04820 (387 aa)<br>Deide_1p01286 (378 aa)<br>Deide_06250 (417aa)<br>Deide_10420 (434 aa)<br>Deide_21550 (366 aa)	DR_0327 (364 aa)<br>DR_0745 (366 aa)<br>DR_1599 (470 aa)<br>DR_1756 (441 aa)<br>DR_0984 frameshift<br>DR_0300 frameshift	Dgeo_2185 (384 aa)<br>Dgeo_2025 (375 aa)<br>Dgeo_1495 (373 aa)<br>Dgeo_0676 (426 aa)<br>Dgeo_0552 (423 aa)<br>Dgeo_0103 (367 aa)	Do serine protease, with PDZ domain
prc	Deide_21230 (445 aa)<br>Deide_12780 (418 aa)<br>Deide_10700 (438 aa)	DR_1308 (447 aa)<br>DR_1491 (445 aa)<br>DR_1551 (447 aa)	Dgeo_0277 (447 aa)<br>Dgeo_1216 (438 aa)<br>Dgeo_1479 (449 aa)	Tail-specific periplasmic serine protease
yaeL	Deide_13240 (376 aa)	DR_1507 (377 aa)	Dgeo_1043 (372 aa)	Membrane-associated Zn-dependent protease I

ftsH	Deide_23310 (626 aa)<br>Deide_18550 (618 aa)<br>Deide_01120 (622 aa)	DR_0583 (618 aa)<br>DR_1020 (655 aa)<br>DR_A0290 (655 aa)	Dgeo_2075 (621 aa)<br>Dgeo_1832 (623 aa)<br>Dgeo_2182 (621 aa)	ATP-dependent Zn protease
htpX	Deide_21060 (354 aa)<br>Deide_11220 (228 aa)	 DR_0194 (227 aa)	 Dgeo_1282 (226 aa)	Predicted Zn-dependent proteases (possible chaperone)
sugE	Deide_20500 (106 aa)	DR_1004 (103 aa)<br>DR_1005 (113 aa)	Dgeo_2170 (106 aa)<br>Dgeo_1957 (112 aa)<br>Dgeo_1956 (111 aa)	Small multidrug resistance membrane protein
hit	Deide_15190 (115 aa)<br>Deide_12650 (137 aa)	DR_1621 (118 aa)	Dgeo_1329 (114 aa)<br>Dgeo_2064 (164 aa)	Diadenosine tetraphosphate (Ap4A) hydrolase, HIT family, cell cycle regulation
yebL	Deide_07330 (299 aa)<br>Deide_3p02720 (322 aa)	DR_2523 frameshift	Dgeo_0534 (293 aa)	Zn-binding (lipo)protein of the ABC type Zn transport system (surface adhesin A)
hflX	Deide_19040 (569 aa)<br>Deide_18500 (307 aa)	DR_0139 (525 aa)<br>DR_0646 (311 aa)	Dgeo_2006 (569 aa)<br>Dgeo_1825 (309 aa)	GTPase, protease modulator
BS_*ytxJ*	Deide_14700 (217 aa)	DR_1832 (211 aa)	Dgeo_1464 (219 aa)	Thioredoxin-like
thiJ	Deide_04910 (189 aa)	DR_1199 (190 aa)<br>DR_0491 (261 aa)	Dgeo_0863 (188 aa)	Protease I, related to general stress protein 18, ThiJ superfamily protein
uspA	Deide_22300 (177 aa)<br>Deide_08840 (164 aa)<br><br>Deide_3p00273 (301 aa)<br>Deide_1p01770 (230 aa)	DR_2363 (160 aa)<br>DR_2132 (150 aa)	Dgeo_0155 (177 aa)<br>Dgeo_1279 (158 aa)<br>Dgeo_2742 (157 aa)	Universal stress protein, nucleotide-binding
**Starvation**				
spoT	Deide_14760 (760 aa)	DR_1838 (787 aa)	Dgeo_1308 (766 aa)	Guanosine polyphosphate (ppGpp) pyrophosphohydrolase/synthetase
mazF	-<br>-	DR_0417 (117 aa)<br>DR_0662 (115 aa)	Dgeo_1937 (110 aa)	ppGpp regulated growth inhibitor
mazE	-	DR_0416 (80 aa)	Dgeo_1936 (88 aa)	Regulatory protein, MazF antagonist
ppx	Deide_01820 (500 aa)	DR_A0185 (515 aa)	-	Phosphatase of ppGpp
dps	Deide_21200 (203 aa)	DR_2263 (207 aa)<br>DR_B0092 (241 aa)	Dgeo_0281 (222 aa)	Starvation inducible DNA-binding protein
cstA	-	-	-	Carbon starvation-induced protein, membrane
**Osmotic**				
mscL	Deide_02220 (131 aa)	DR_2422 (128 aa)	Dgeo_0305 (131 aa)	Large conductance mechano-sensitive channel
yggB	Deide_18560 (417 aa)<br>-	DR_1995 (426 aa)<br>DR_0211 (368 aa)	Dgeo_1833 (394 aa)	Membrane protein
kdpD	-	DR_B0088 (365aa)	Dgeo_0389 (557aa)<br>Dgeo_2852 (365 aa)	Osmosensitive K+ channel histidine kinase sensor domain
trkA	Deide_03820 (219 aa)	DR_1666 (226 aa)	Dgeo_1584 (219 aa)	Potassium uptake system, NAD-binding component

trkH/*trkG*	Deide_03830 (425 aa)<br>Deide_3p00460 (451 aa)	DR_1667 (458 aa)<br>DR_1668 (512 aa)	Dgeo_1583 (458 aa)	Potassium uptake component
proW	Deide_00160 (239 aa)<br>Deide_00180 (393 aa)	DR_A0138 (247 aa)<br>DR_A0136 (484 aa)	Dgeo_0171 (249 aa)<br>Dgeo_0173 (397 aa)	Proline/glycine betaine ABC-type transport, permease subunit
proV	Deide_00170 (308 aa)	DR_A0137 (309 aa)	Dgeo_0172 (310 aa)	Proline/glycine betaine ABC-type transport, ATPase subunit
yehZ	Deide_00190 (301 aa)	DR_A0135 (369 aa)	Dgeo_0174 (300 aa)	Proline/glycine betaine ABC-type transport, periplasmic subunit
otsA	Deide_21370 (457 aa)	-	Dgeo_0059 (457 aa)	Trehalose-6-phosphate synthase
otsB	Deide_21360 (239 aa)	-	Dgeo_0060 (238 aa)	Trehalose-6-phosphatase
	Deide_16570 (954 aa)	DR_0463 (978 aa)	Dgeo_0539 (944 aa)	Maltooligosyltrehalose synthase TreY
	Deide_2p01290 (597 aa)	DR_0464 (600 aa)	Dgeo_0540 (603 aa)	Maltooligosyltrehalose trehalohydrolase TreZ
	Deide_07350 (552 aa)	DR_2036 (552 aa)	Dgeo_0537 (556 aa)	Trehalose synthase-like TreS
aqpZ<br><br>*glpF*	Deide_1p01582 (245 aa)<br>Deide_3p02450 (232 aa)<br>Deide_2p00230 (276 aa)	-<br>-<br>DR_1929 (274 aa)	Dgeo_0516 (259 aa)<br>Dgeo_1799 (221 aa)<br>Dgeo_2523 (274 aa)	Major intrinsic protein (aquaporin Z and glycerol uptake facilitator)
**Oxidative / detoxication**				
oxyR	Deide_03130 (301 aa)<br>Deide_16400 (318 aa)<br>Deide_3p01240 (298 aa)	DR_0615 (317 aa)<br>-<br>-	Dgeo_1888 (297 aa)<br>Dgeo_1692 (329 aa)<br>Dgeo_2711 (292 aa)<br>Dgeo_2840 (299 aa)	Transcriptional regulator, LysR family
soxR	Deide_2p01800 (285 aa)<br>-<br>Deide_3p02530 (131 aa)<br>Deide_3p02771 (139 aa)<br>Deide_04091 (157 aa)	DR_2448 (280 aa)<br>DR_2519 (127 aa)<br>-<br>-<br>-	Dgeo_1424 (279 aa)<br>-<br>-<br>-<br>-	Transcriptional regulator, MerR family
katE	-<br>Deide_2p00330 (540 aa)	DR_A0259 (772 aa)<br>DR_1998 (536 aa)	-<br>Dgeo_2728 (543 aa)	Catalase
katA S. pombe	-	DR_A0146 (361 aa)	-	Catalase; Eukaryotic type
-	-	DR_A0145 (460 aa)	-	Fe dependent peroxidase
katG	-	-	-	Catalase (peroxidase)
-	-	-	-	Mn-containing catalase
sodA	Deide_07760 (207 aa)	DR_1279 (211 aa)	Dgeo_0830 (212 aa)	Superoxide dismutase Mn or Fe dependent
sodC	-<br>Deide_1p00740 (468 aa)<br>Deide_19880 (182 aa)	DR_1546 (182 aa)<br>DR_A0202 (462 aa)<br>-	-<br>-<br>-	Superoxide dismutase Cu/Zn dependent
fur	Deide_16041 (116 aa)<br>Deide_19480 (133 aa)<br>Deide_2p00340 (142 aa)	DR_0865 (132 aa)<br>Upstr DR_2342	Dgeo_0519 (135 aa)<br>Dgeo_2141 (137 aa)<br>Dgeo_2727 (135 aa)	Ferric uptake regulation protein
bcp	Deide_10900 (164 aa)	DR_0846 (175 aa)	Upstr Dgeo_1265	Peroxiredoxin, bacterioferritin comigratory protein, antioxidant

	Deide_09051 (170 aa)<br>-<br>-	DR_1209 (159 aa)<br>DR_1208 (163 aa)<br>-	(167-178 aa)<br>Dgeo_0990 (152 aa)<br>-<br>Dgeo_2729 (159 aa)	protein
tlp-like	Deide_08290 (192 aa)<br>-<br>Deide_2p00420 (174 aa)	DR_0189 (185 aa)<br>DR_0345 (188 aa)<br>-	Dgeo_1248 (192 aa)<br>-<br>-	Peroxiredoxin, disulfide reductase
osmC	Deide_16090 (147 aa)	DR_1538 (172 aa)<br>DR_1857 (139 aa)	Dgeo_0526 (145 aa)<br>Dgeo_0446 (146 aa)	Protein involved in alkylperoxide and oxidative stress response, osmotically induced protein
yhfA	Deide_10790 (132 aa)	DR_1177 (140 aa)	Dgeo_1268 (132 aa)	Protein involved in alkylperoxide and oxidative stress response, osmotically induced protein
msrA	Deide_10980 (195 aa)	DR_1849 (206 aa)	Dgeo_0843 (209 aa)	Peptide methionine sulfoxide reductase A
msrB	Deide_04050 (150 aa)	DR_1378 (157 aa)	Dgeo_2072 (152 aa)	Peptide methionine sulfoxide reductase B
ahpC/ahpE-like	Deide_02430 (151 aa)	DR_2242 (151 aa)	Dgeo_0122 (151 aa)	Thioredoxin reductase/alkyl hydroperoxide reductase
ahpf/trxB	Deide_05800 (325 aa)<br>Deide_23360 (336 aa)<br>Deide_12541 (356 aa)<br>Deide_09090 (321 aa)	DR_1982 (325 aa)<br>DR_2623 (332 aa)<br>DR_0412 (324 aa)<br>DR_B0033 (833 aa)	Dgeo_1576 (327 aa)<br>Dgeo_2772 (321 aa)<br>Dgeo_1013 (331 aa)<br>Dgeo_2331 (338 aa)<br>Dgeo_0975 (320 aa)	Thiol-alkyl hydroperoxide reductase
grxA<br><br>*nrdH*	Deide_06390 (81 aa)<br>-<br>Deide_13741 (92 aa)	DR_2085 (81 aa)<br>DR_A0072 (91 aa)<br>DR_0057 (84 aa)	Dgeo_1508 (81 aa)<br>Dgeo_2583 (84 aa)<br>Dgeo_0729 (87 aa)	Glutaredoxin
trx	Deide_01140 (138 aa)<br>Deide_18600 (121 aa)	DR_A0164 (142 aa)<br>DR_0944 (141 aa)	Dgeo_2518 (141 aa)<br>Dgeo_1837 (110 aa)	Thioredoxin
btuE /BS_*bsaA*	-	-	-	Glutathione peroxidase
BS_*cyp*	Deide_22920 (408 aa)<br>Deide_01550 (421 aa)<br>Deide_08170 (405 aa)	DR_2538 (409 aa)<br><br><br>DR_2473 (381 aa)<br>DR_1723 (445 aa)<br>DR_C0001 (103 aa)<br>DR_A0186 (fr.shift)	 Dgeo_0143 (407 aa)<br>Dgeo_0944 (405 aa)	Cytochrome P450
BS_*yceH*	Deide_05270 (405 aa)	DR_1127 (416 aa)	Dgeo_0931 (406 aa)	Toxic anion resistance protein, possibly tellurite resistance
**Desiccation**				
-	Deide_09710 (166 aa)	DR_1372 (164 aa)	Dgeo_1551 (166 aa)	Desiccation-related protein, LEA 14 family
-	Deide_07540 (320 aa)<br>Deide_08080 (312 aa)	DR_B0118 (337 aa)	Dgeo_0097 (311 aa)<br>Dgeo_1323 (307 aa)	Desiccation-related protein
-	Deide_08510 (200 aa)	DR_1172 (298 aa)<br>DR_0105 (163 aa)	Dgeo_1473 (236 aa)	LEA76 family desiccation resistance protein

**Other**				
hupA	Deide_2p01940 (125 aa)<br>Deide_00200 (115 aa)<br>Deide_3p00060 (121 aa)<br>Deide_3p00832 (166 aa)	DR_A0065 (122 aa)	Dgeo_2501 (149 aa)<br>Dgeo_0175 (116 aa)	Histone-like nucleoid DNA-binding protein
pspA	Deide_09450 (226 aa)	DR_1473 (223 aa)	Dgeo_0996 (227 aa)	Phage shock protein A, controls membrane integrity
BS_*yloU*/<br>BS_*yqhY*	Deide_12280 (116 aa)<br>Deide_17570 (110 aa)	DR_2068 (135 aa)<br>DR_0389 (110 aa)	Dgeo_0697 (152 aa)<br>Dgeo_1721 (110 aa)	Alkaline shock protein, function unknown
cps	Deide_3p00840 (87 aa)<br>Deide_2p00490 (87 aa)<br>Deide_09930 (94 aa)	DR_0907 (86 aa)	Dgeo_0638 (87 aa)<br>Dgeo_1006 (89 aa)	Cold shock protein, OB fold nucleic acid binding protein
arsC	Deide_3p02440 (153 aa)<br>Deide_18400 (118 aa)	DR_A0123 (180 aa)<br>DR_0136 (127 aa)	Dgeo_0756 (153 aa)<br>Dgeo_0403 (118 aa)<br>Dgeo_2548 (153 aa)<br>Dgeo_2768 (153 aa)<br>Dgeo_2770 (143 aa)	Arsenate oxidoreductase (ArsC-like Rodanese protein)

[a]The gene names are from *E. coli*, whenever an *E. coli* ortholog exists, or from *B. subtilis* (with the prefix BS_). Proteins in grey were identified in the proteome analysis after standard cultivation.
