# Supplementary material for: Alliance of Proteomics and Genomics to Unravel the Specificities of Sahara Bacterium Deinococcus deserti
Source: PLoS Genet. 2009 Mar 27;5(3):e1000434. doi: 10.1371/journal.pgen.1000434 (PMC2669436; doi:10.1371/journal.pgen.1000434)
Supplement: Table S9 — ABC-transporters identified in D. deserti. (0.15 MB PDF) [file pgen.1000434.s014.pdf]

Table S9. ABC transporters in *D. deserti*						
COG number	Protein annotation	Name	*D. radiodurans*	*D. geothermalis*	*D. deserti*	Spectral count
COG0747	ABC-type dipeptide transport system (periplasmic component)	DdpA	DR_A0210	Dgeo_2510	Deide_2p00290	112
COG0747	ABC-type dipeptide transport system (periplasmic component)	DdpA	DR_1571	Dgeo_1344	Deide_3p01890,<br>Deide_11060	73<br>596
COG0747	ABC-type dipeptide transport system (periplasmic component)	DdpA	-	-	Deide_1p01230,<br>Deide_3p01130	0<br>6
COG0747	ABC-type dipeptide transport system (periplasmic component)	DdpA	-	Dgeo_0324	-	
COG0747	ABC-type dipeptide transport system (periplasmic component)	DdpA	-	-	Deide_3p00570,<br>Deide_1p01480,<br>Deide_3p00780,<br>Deide_3p02230	0<br>167<br>10<br>4
COG0747	ABC-type dipeptide transport system (periplasmic component)	DdpA	-	-	Deide_3p00722	0
COG0747	ABC-type dipeptide transport system (periplasmic component)	DdpA	DR_0363,<br>DR_0986	Dgeo_2122,<br>Dgeo_0554	Deide_19110,<br>Deide_06270	683<br>0
COG0747	ABC-type dipeptide transport system (periplasmic component)	DdpA	DR_1290, DR_A0246,<br>DR_1712, DR_1955		Deide_10250,<br>Deide_3p02270	89<br>14
COG0601	ABC-type dipeptide transport system (permease component)	DppB	-	-	Deide_1p01490	2
COG0601	ABC-type dipeptide transport system (permease component)	DppB	-	-	Deide_1p01220	0
COG0601	ABC-type dipeptide transport system (permease component)	DppB	DR_A0209	Dgeo_2511	Deide_2p00280	0
COG0601	ABC-type dipeptide transport system (permease component)	DppB	-	Dgeo_0325	-	
COG0601	ABC-type dipeptide transport system (permease component)	DppB	DR_0959	Dgeo_1190	Deide_10240	4

COG0601	ABC-type dipeptide/oligopeptide/nickel transport system (permease component)	DppB	-	-	Deide_3p01120	0
COG0601	ABC-type dipeptide/oligopeptide/nickel transport system (permease component)	DppB	-	-	Deide_3p00790	0
COG0601	ABC-type dipeptide/oligopeptide/nickel transport system (permease component)	DppB	-	-	Deide_3p00580	0
COG0601	ABC-type dipeptide/oligopeptide/nickel transport system (permease component)	DppB	-	-	Deide_3p02240	0
COG0601	ABC-type dipeptide/oligopeptide/nickel transport system (permease component)	DppB	DR_0364	Dgeo_2123	Deide_19120	7
COG0601	ABC-type dipeptide/oligopeptide/nickel transport system (permease component)	DppB	DR_1570	Dgeo_1343	Deide_11070	0
COG1173	ABC-type oligopeptide transport system (permease component)	DppC	DR_A0208	Dgeo_2512	Deide_2p00270	0
COG1173	ABC-type dipeptide transport system (permease component)	DppC	-	Dgeo_0326	-	0
COG1173	ABC-type dipeptide transport system (permease component)	DppC	-	-	Deide_1p01210	0
COG1173	ABC-type dipeptide transport system (permease component)	DppC	-	-	Deide_3p02250	0
COG1173	ABC-type dipeptide transport system (permease component)	DppC	-	-	Deide_1p01500	0
COG1173	ABC-type dipeptide transport system (permease component)	DppC	DR_0958	Dgeo_1191	Deide_10230	2

COG1173	ABC-type dipeptide/oligopeptide/nickel transport system (permease component)	DppC	-	-	Deide_3p01110	0
COG1173	ABC-type dipeptide/oligopeptide/nickel transport system (permease component)	DppC	-	-	Deide_3p00800	0
COG1173	ABC-type dipeptide/oligopeptide/nickel transport system (permease component)	DppC	-	-	Deide_3p00590	0
COG1173	ABC-type dipeptide/oligopeptide/nickel transport system (permease component)	DppC	DR_0365	Dgeo_2124	Deide_19130	0
COG1173	ABC-type dipeptide/oligopeptide/nickel transport system (permease component)	DppC	DR_1569	Dgeo_1342	Deide_11080	2
COG0444	ABC-type dipeptide/oligopeptide/nickel transport system (ATPase component)	DppD	DR_1568	Dgeo_1341	Deide_11090	29
COG0444	ABC-type dipeptide/oligopeptide/Nickel transport system (ATPase component)	DppD	-	-	Deide_3p00600	0
COG4608	ABC-type oligopeptide transport system (ATPase component)	AppF	DR_1567	Dgeo_1340	Deide_11100	41
COG4608	ABC-type oligopeptide transport system (ATPase component)	AppF	-	-	Deide_3p00610	0
COG4177	ABC-type branch-chain amino acid transport system (permease component)	LivM	-	Dgeo_2032	Deide_04750	0
COG4177	ABC-type branch-chain amino acid transport system (permease component)	LivM	-	-	Deide_14430	0

**COG4177**	**ABC-type branch-chain amino acid transport system (permease component)**	**LivM**	**-**	**Dgeo_0801**	**Deide_14790**	**0**
**COG4177**	**ABC-type branch-chain amino acid transport system (permease component)**	**LivM**	**-**	**-**	**Deide_12200**	**0**
**COG4177**	**ABC-type branch-chain amino acid transport system (permease component)**	**LivM**	**DR_0282**	**Dgeo_2167**	**Deide_20470**	**0**
**COG4177**	**ABC-type branch-chain amino acid transport system (permease component)**	**LivM**	**DR_1036**	**Dgeo_1207**	**Deide_12610**	**3**
**COG4177**	**ABC-type branch-chain amino acid transport system (permease component)**	**LivM**	**DR_2120**	**Dgeo_0733**	**Deide_07000**	**0**
**COG0559**	**ABC-type short chain or branch-chain amino acid transport system (permease component)**	**LivH**	**-**	**Dgeo_0802**	**Deide_14800**	**0**
**COG0559**	**ABC-type short chain or branch-chain amino acid transport system (permease component)**	**LivH**	**-**	**Dgeo_2031**	**Deide_04760**	**0**
**COG0559**	**ABC-type short chain or branch-chain amino acid transport system (permease component)**	**LivH**	**-**	**-**	**Deide_12210**	**0**
**COG0559**	**ABC-type short chain or branch-chain amino acid transport system (permease component)**	**LivH**	**-**	**-**	**Deide_14440**	**0**
**COG0559**	**ABC-type short chain or branch-chain amino acid transport system (permease component)**	**LivH**	**DR_A0262**	**Dgeo_2424**	**Deide_2p00160**	**0**
**COG0559**	**ABC-type short chain or branch-chain amino acid transport system (permease component)**	**LivH**	**DR_0281**	**Dgeo_2166**	**Deide_20460**	**0**
**COG0559**	**ABC-type short chain or branch-chain amino acid transport system (permease component)**	**LivH**	**DR_2121**	**Dgeo_0734**	**Deide_07010**	**0**

**COG0559**	**ABC-type short chain or branch-chain amino acid transport system (permease component)**	**LivH**	**DR_1037**	**Dgeo_1206**	**Deide_12600**	**1**
**COG0683**	**ABC-type branch-chain amino acid transport system (periplasmic component)**	**LivK**	**-**	**-**	**Deide_12220**	**60**
**COG0683**	**ABC-type branch-chain amino acid transport system (periplasmic component)**	**LivK**	**-**	**-**	**Deide_14420**	**32**
**COG0683**	**ABC-type branch-chain amino acid transport system (periplasmic component)**	**LivK**	**DR_1038**<br>**DR_0788**	**Dgeo_1205**<br>**Dgeo_1636**<br>**Dgeo_0620**	**Deide_12590**<br>**Deide_14600**<br>**Deide_17040**	**389**<br>**0**<br>**0**
**COG0683**	**ABC-type branch-chain amino acid transport system (periplasmic component)**	**LivK**	**-**	**Dgeo_0806**	**Deide_14810**	**19**
**COG0683**	**ABC-type branch-chain amino acid transport system (periplasmic component)**	**LivK**	**-**	**Dgeo_2030**	**Deide_04770**	**49**
**COG0683**	**ABC-type branch-chain amino acid transport system (periplasmic component)**	**LivK**	**DR_A0263**	**Dgeo_2411**	**Deide_2p00130**	**25**
**COG0683**	**ABC-type branch-chain amino acid transport system (periplasmic component)**	**LivK**	**DR_A0260**	**Dgeo_2426**	**Deide_2p00140**	**4**
**COG0683**	**ABC-type branch-chain amino acid transport system (periplasmic component)**	**LivK**	**DR_0280**	**Dgeo_2165**	**Deide_20450**	**132**
**COG0683**	**ABC-type branch-chain amino acid transport system (periplasmic component)**	**LivK**	**DR_2122**	**Dgeo_0735**	**Deide_07020**	**18**
**COG0410**	**ABC-type branch-chain amino acid transport system (ATPase component)**	**LivF**	**-**	**-**	**Deide_14410**	**0**
**COG0410**	**ABC-type branch-chain amino acid transport system (ATPase component)**	**LivF**	**DR_1034**	**Dgeo_1209**	**Deide_12630**	**16**

**COG0410**	**ABC-type branch-chain amino acid transport system (ATPase component)**	**LivF**	**-**	**Dgeo_0799**	**Deide_14770**	**0**
**COG0410**	**ABC-type branch-chain amino acid transport system (ATPase component)**	**LivF**	**-**	**Dgeo_2029**	**Deide_04780**	**0**
**COG0410**	**ABC-type branch-chain amino acid transport system (ATPase component)**	**LivF**	**-**	**-**	**Deide_12180**	**0**
**COG0410**	**ABC-type branch-chain amino acid transport system (ATPase component)**	**LivF**	**DR_0284**	**Dgeo_2169**	**Deide_20490**	**0**
**COG0410**	**ABC-type branch-chain amino acid transport system (ATPase component)**	**LivF**	**DR_2118**	**Dgeo_0731**	**Deide_06980**	**4**
**COG0411**	**ABC-type branch-chain amino acid transport system (ATPase component)**	**LivG**	**-**	**-**	**Deide_12190**	**0**
**COG0411**	**ABC-type branch-chain amino acid transport system (ATPase component)**	**LivG**	**-**	**-**	**Deide_14460**	**1**
**COG0411**	**ABC-type branch-chain amino acid transport system (ATPase component)**	**LivG**	**DR_0283**	**Dgeo_2168**	**Deide_20480**	**1**
**COG0411**	**ABC-type branch-chain amino acid transport system (ATPase component)**	**LivG**	**DR_1035**	**Dgeo_1208**	**Deide_12620**	**27**
**COG0411**	**ABC-type branch-chain amino acid transport system (ATPase component)**	**LivG**	**-**	**Dgeo_0800**	**Deide_14780**	**0**
**COG0411**	**ABC-type branch-chain amino acid transport system (ATPase component)**	**LivG**	**-**	**Dgeo_2028**	**Deide_04790**	**0**
**COG0411**	**ABC-type branch-chain amino acid transport system (ATPase component)**	**LivG**	**DR_2119**	**Dgeo_0732**	**Deide_06990**	**0**

**COG4177-COG0411**	**ABC-type branch-chain amino acid transport system (permease - ATPase component)**	**LivM-LivG**	**DR_A0261**	**Dgeo_2425**	**Deide_2p00150**	**0**
**COG0834**	**ABC-type amino acid transport system (periplasmic component)**	**HisJ**	**DR_2154<br>DR_2610<br>DR_B0078<br>DR_0564**	**Dgeo_2189<br>Dgeo_1060<br>Dgeo_1986**	**Deide_05450<br>Deide_07680<br>Deide_2p01060<br>Deide_3p00160<br>Deide_3p00200**	**3<br>100<br>1<br>10<br>165**
**COG0765**	**ABC-type amino acid transport system permease component)**	**HisM**	**DR_2155<br>DR_1028**	**Dgeo_1061**	**Deide_07690**	**0**
**COG1126**	**ABC-type polar amino acid transport system (ATPase component)**	**GlnQ**	**DR_1648**	**Dgeo_1064**	**Deide_07730**	**3**
**COG1174**	**ABC-type proline/glycine betaine transport system (permease component)**	**OpuBB**	**DR_A0138**	**Dgeo_0171**	**Deide_00160**	**0**
**COG1174**	**ABC-type proline/glycine betaine transport system (permease component)**	**OpuBB**	**DR_A0136**	**Dgeo_0173**	**Deide_00180**	**0**
**COG1125**	**ABC-type proline/glycine betaine transport system (ATPase component)**	**OpuBA**	**DR_A0137**	**Dgeo_0172**	**Deide_00170**	**0**
**COG1732**	**ABC-type glycine betaine/choline transport system (periplasmic component)**	**OpuBC**	**DR_A0135**	**Dgeo_0174**	**Deide_00190**	**22**
**COG1131**	**ABC-type multidrug transport system (ATPase component)**	**CcmA**	**DR_2316**	**Dgeo_0381**	**Deide_21040**	**0**
**COG1131**	**ABC-type multidrug transport system (ATPase component)**	**CcmA**	**DR_1012**	**Dgeo_1811**	**Deide_17730**	**5**
**COG1131**	**ABC-type multidrug transport system (ATPase component)**	**CcmA**	**-**	**-**	**Deide_08880**	**0**
**COG1131**	**ABC-type multidrug transport system (ATPase component)**	**CcmA**	**DR_0406**	**Dgeo_1242**	**Deide_08340**	**0**
**COG1132**	**ABC-type multidrug transport system (ATPase & permease components)**	**MdlB**	**DR_2051<br>DR_A0349**	**Dgeo_0647<br>Dgeo_1385**	**Deide_06130**	**0**

COG1132	ABC-type multidrug transport system (ATPase & permease components)	MdlB	DR_2052	Dgeo_0648	Deide_06140	1
COG1132	ABC-type multidrug transport system (ATPase & permease components)	MdlB	DR_0163	-	Deide_00460	0
COG1132	ABC-type multidrug transport system (ATPase & permease components)	MdlB	DR_0096	-	Deide_19830	0
COG1132	ABC-type multidrug transport system (ATPase & permease components)	MdlB	DR_0095	-	Deide_19840	0
COG1175	ABC-type sugar transport system (permease component)	UgpA	DR_1437	Dgeo_0590	Deide_14330<br>Deide_3p01820	0<br>0
COG1175	ABC-type sugar transport system (permease component)	UgpA	-	Dgeo_1168<br>Dgeo_2918	Deide_13600<br>Deide_3p01660	0<br>0
COG1175	ABC-type sugar transport system (permease component)	UgpA	DR_0562	Dgeo_1501	Deide_09490	0
COG1175	ABC-type sugar transport system (permease component)	UgpA	-	Dgeo_0906	Deide_05060	0
COG1175	ABC-type sugar transport system (permease component)	UgpA	-	Dgeo_2820	Deide_1p01610<br>Deide_3p00100	0<br>0
COG1175	ABC-type sugar transport system (permease component)	UgpA	-	Dgeo_2725	Deide_02540	0
COG1175	ABC-type sugar transport system (permease component)	UgpA	-	Dgeo_2695<br>Dgeo_2918	Deide_3p00980<br>Deide_3p01950	0<br>0
COG1175	ABC-type sugar transport system (permease component)	UgpA	-	Dgeo_2869	Deide_3p02100	0
COG1175	ABC-type sugar transport system (permease component)	UgpA	-	Dgeo_2860	Deide_00570	0
COG1175	ABC-type sugar transport system (permease component)	UgpA	-	-	Deide_1p00650	0
COG1653	ABC-type sugar transport system (periplasmic component)	UgpB	DR_1438	Dgeo_0591	Deide_14320<br>Deide_2p01740	564<br>3

COG1653	ABC-type sugar transport system (periplasmic component)	UgpB	-	Dgeo_2821	Deide_1p01600<br>Deide_3p00090<br>Deide_3p00990	0<br>15<br>16
COG1653	ABC-type sugar transport system (periplasmic component)	UgpB	-	-	Deide_3p01810	16
COG1653	ABC-type sugar transport system (periplasmic component)	UgpB	-	Dgeo_2870	Deide_3p02090	55
COG1653	ABC-type sugar transport system (periplasmic component)	UgpB	-	Dgeo_2696	Deide_3p01960	16
COG1653	ABC-type sugar transport system (periplasmic component)	UgpB	-	Dgeo_0905	Deide_05050	1128
COG1653	ABC-type sugar transport system (periplasmic component)	UgpB	-	-	Deide_3p01640	0
COG1653	ABC-type sugar transport system (periplasmic component)	UgpB	-	-	Deide_03790	0
COG1653	ABC-type sugar transport system (periplasmic component)	UgpB	-	-	Deide_1p00660	44
COG1653	ABC-type sugar transport system (periplasmic component)	UgpB	-	Dgeo_2726<br>Dgeo_2919	Deide_02550	183
COG1653	ABC-type sugar transport system (periplasmic component)	UgpB	-	Dgeo_2859	Deide_00580	3
COG1653	ABC-type sugar transport system (periplasmic component)	UgpB	-	Dgeo_0687	Deide_10270	35
COG0395	ABC-type sugar transport system (permease component)	UgpE	DR_1436	Dgeo_0589	Deide_14340	1
COG0395	ABC-type sugar transport system (permease component)	UgpE	-	-	Deide_1p00640	0
COG0395	ABC-type sugar transport system (permease component)	UgpE	-	Dgeo_2694	Deide_3p01940	0
COG0395	ABC-type sugar transport system (permease component)	UgpE	-	Dgeo_2917	Deide_3p01650	0
COG0395	ABC-type sugar transport system (permease component)	UgpE	-	Dgeo_1169<br>Dgeo_2724	Deide_13610	0

**COG0395**	**ABC-type sugar transport system (permease component)**	**UgpE**	**-**	**Dgeo_2818**	**Deide_1p01620**<br>**Deide_3p00110**<br>**Deide_3p00970**<br>**Deide_3p01830**	**0**<br>**0**<br>**0**<br>**0**
**COG0395**	**ABC-type sugar transport system (permease component)**	**UgpE**	**-**	**Dgeo_0907**	**Deide_05070**	**0**
**COG0395**	**ABC-type sugar transport system (permease component)**	**UgpE**	**-**	**Dgeo_2724**	**Deide_02530**	**0**
**COG0395**	**ABC-type sugar transport system (permease component)**	**UgpE**	**-**	**Dgeo_2868**	**Deide_3p02110**	**0**
**COG0395**	**ABC-type sugar transport system (permease component)**	**UgpE**	**-**	**Dgeo_2861**	**Deide_00560**	**0**
**COG1137**	**ABC-type unclassified transport system (ATPase component)**	**HhbG**	**DR_2134**	**Dgeo_0587**	**Deide_03770**	**0**
**COG3833**	**ABC-type sugar transport system (permease component)**	**MalG**	**DR_0563**	**Dgeo_1502**	**Deide_09480**	**0**
**COG2182**	**ABC-type sugar transport system (periplasmic component)**	**MalE**	**DR_0561**	**-**	**Deide_09500**	**249**
**COG3839**	**ABC-type sugar transport system (ATPase component)**	**MalK**	**DR_2153**	**Dgeo_1059**	**Deide_07670**	**68**
**COG1129**	**ABC-type sugar transport system (ATPase component)**	**MglA**	**-**	**Dgeo_2458**	**Deide_3p01510 / Deide_3p01501**	**0**
**COG1129**	**ABC-type sugar transport system (ATPase component)**	**MglA**	**-**	**-**	**Deide_3p02590**	**0**
**COG1879**	**ABC-type sugar transport system (periplasmic component)**	**RbsB**	**-**	**Dgeo_2461**	**Deide_3p01480**	**37**
**COG1879**	**ABC-type sugar transport system (periplasmic component)**	**RbsB**	**-**	**-**	**Deide_3p02570**	**55**
**COG1172**	**ABC-type sugar transport system (permease component)**	**AraH**	**-**	**Dgeo_2460**	**Deide_3p01490**	**0**
**COG1172**	**ABC-type sugar transport system (permease component)**	**AraH**	**-**	**Dgeo_2459**	**Deide_3p01500**	**0**
**COG1172**	**ABC-type sugar transport system (permease component)**	**AraH**	**-**	**-**	**Deide_3p02580**	**0**
**COG1172**	**ABC-type sugar transport system (permease component)**	**AraH**	**-**	**-**	**Deide_3p02600**	**0**

COG1119	ABC-type molybdenum transport system (ATPase component) / Photorepair protein PhrA	ModF/PhrA ?	DR_2145	Dgeo_1736	Deide_07260	0
COG4149	ABC-type molybdate transport system (permease component)	ModC	-	-	Deide_2p02120	0
COG0725	ABC-type molybdate transport system (periplasmic component)	ModA	-	-	Deide_2p02121	15
COG1277	ABC-type transport system involved in multi-copper enzyme maturation (permease component)	NosY	DR_2315	Dgeo_0382	Deide_21030	0
COG1464	ABC-type metal ion transport system (periplasmic component)	NlpA	DR_1359<br>DR_1358	-	Deide_22340	34
COG2011	ABC-type metal ion transport system (permease component)	AbcD	DR_1357	-	Deide_22350	0
COG1135	ABC-type metal ion transport system (ATPase component)	AbcC	DR_1356	-	Deide_22360	0
COG0715-COG4521	ABC-type nitrate/sulfonate/bicarbonate transport system (periplasmic component)	TauA	DR_1277	Dgeo_1411	Deide_13940	23
COG0715-COG4521	ABC-type nitrate/sulfonate/bicarbonate transport system (periplasmic component)	TauA	DR_1655	Dgeo_0543	Deide_16540	9
COG1116	ABC-type nitrate/sulfonate/bicarbonate transport system (ATPase component)	TauB	DR_2198	Dgeo_1413	Deide_13960	1
COG0600	ABC-type nitrate/sulfonate/bicarbonate transport system (permease component)	TauC	DR_2197	Dgeo_1412	Deide_13950	0
COG3842	ABC-type spermidine/putrescine transport system (ATPase component)	PotA	-	-	Deide_19070	0

**COG3842**	**ABC-type spermidine/putrescine transport system (ATPase component)**	**PotA**	**DR_0062**	**Dgeo_1938**	**Deide_15860**	**0**
**COG4143**	**ABC-type thiamine transport system (periplasmic component)**	**TbpA**	**DR_0262**	**Dgeo_1933**	**Deide_15830**	**2**
**COG???**	**ABC-type Na2+ efflux pump transport system (ATPase component)**	**NatA**	**DR_0927**	**Dgeo_1675**	**Deide_15090**	**4**
**COG???**	**ABC-type Na2+ efflux pump transport system (permease component)**	**NatB**	**DR_0926**	**Dgeo_1676**	**Deide_15080**	**0**
**COG0226**	**ABC-type phosphate transport system (periplasmic component)**	**PstS**	**DR_A0157**	**Dgeo_0649**	**Deide_2p01880**	**5**
**COG0573**	**ABC-type phosphate transport system (permease component)**	**PstC**	**DR_A0158**	**Dgeo_0650**	**Deide_2p01890**	**1**
**COG0581**	**ABC-type phosphate transport system (permease component)**	**PstA**	**DR_A0159**	**Dgeo_0651**	**Deide_2p01900**	**0**
**COG1117**	**ABC-type phosphate transport system (ATPase component)**	**PstB**	**DR_A0160**	**Dgeo_0652**	**Deide_2p01910**	**3**
**COG1136**	**ABC-type antimicrobial peptide transport system (ATPase component)**	**SalX**	**DR_0473**	**Dgeo_1377**	**Deide_13270**	**0**
**COG1136**	**ABC-type antimicrobial peptide transport system (ATPase component)**	**SalX**	**DR_2192**	**Dgeo_0967**	**Deide_10130**	**0**
**COG4591**	**ABC-type lipoprotein transport system (permease component)**	**LolE**	**DR_0474**	**Dgeo_1376**	**Deide_13280**	**0**
**COG1123**	**ABC-type transport system (duplicated ATPase component)**	**?**	**DR_0957**	**Dgeo_1374**	**Deide_08550**	**15**
**COG0488**	**ABC-type transport system (duplicated ATPase component)**	**Uup**	**DR_1103**	**Dgeo_0506**	**Deide_16130**	**0**
**COG0488**	**ABC-type transport system (duplicated ATPase component)**	**Uup**	**DR_1635**	**Dgeo_0929**	**Deide_05240**	**4**
**COG1079**	**uncharacterized ABC-type transport system (permease component)**	**?**	**-**	**Dgeo_1691**	**Deide_07810**	**1**

**COG4603**	**uncharacterized ABC-type transport system (permease component)**	**?**	**DR_0250**	**Dgeo_1690**	**Deide_07820**	**0**
**COG4787**	**uncharacterized ABC-type transport system (permease component)**	**?**	**DR_0203**	**-**	**Deide_08010**	**0**
**COG3694**	**uncharacterized ABC-type transport system (permease component)**	**?**	**DR_0204**	**-**	**Deide_08020**	**0**
**COG3694**	**uncharacterized ABC-type transport system (ATPase component)**	**?**	**DR_1583**	**-**	**Deide_10090**	**0**
**COG3694**	**uncharacterized ABC-type transport system (ATPase component)**	**?**	**DR_0205**	**-**	**Deide_08030**	**0**
**COG4586**	**uncharacterized ABC-type transport system (ATPase component)**	**?**	**DR_1581**	**-**	**Deide_10070**	**0**
**COG4587**	**uncharacterized ABC-type transport system (permease component)**	**?**	**DR_1582**	**-**	**Deide_10080**	**0**
**COG1744**	**ABC-type uncharacterized transport system (periplasmic component)**	**Med/BMP**	**DR_2070**	**Dgeo_0695**<br>**Dgeo_0703**	**Deide_20440**	**132**
