# Supplementary material for: Alliance of Proteomics and Genomics to Unravel the Specificities of Sahara Bacterium Deinococcus deserti
Source: PLoS Genet. 2009 Mar 27;5(3):e1000434. doi: 10.1371/journal.pgen.1000434 (PMC2669436; doi:10.1371/journal.pgen.1000434)
Supplement: Table S10 — DNA repair genes in D. deserti, D. radiodurans and D. geothermalis. (0.20 MB PDF) [file pgen.1000434.s015.pdf]

## Table S10. DNA repair genes in *D. deserti*, *D. radiodurans* and *D. geothermalis*

### A. Main DNA repair genes for Base Excision Repair (BER) [a]

| Gene name | *D. deserti* | *D. radiodurans* | *D. geothermalis* | Putative function of the predicted gene product |
|---|---|---|---|---|
| **Monofunctional DNA glycosylases** | | | | |
| *alkA* (282 aa) | *Deide_08050* (204 aa) | *DR_2074* (190 aa) | *Dgeo_1660* (209 aa) | 3-methyladenine-DNA glycosylase II |
| | *Deide_02320* (216 aa) | *DR_2584* (225 aa) | *Dgeo_0107* (216 aa) | |
| *tag* (187 aa) | - | - | - | 3-methyladenine-DNA glycosylase I |
| *mutY* (350 aa) | *Deide_01970* (353aa) | *DR_2285* (363 aa) | *Dgeo_0019* (343 aa) | A/8oxoG adenine glycosylase |
| *mug* (168 aa) | *Deide_17530* (185 aa) | *DR_0715* (199 aa) | *Dgeo_1718* (195 aa) | Removes uracil, thymine or ethenocytosine opposite guanine |
| | - | - | *Dgeo_2568* (177 aa) (plasmid) | |
| *ung* or *udg* (229 aa) | *Deide_00830* (248 aa) | *DR_0689* (247 aa) | *Dgeo_2059* (245 aa) | Uracil-DNA glycosylase |
| | - | *DR_1751* (237 aa) | *Dgeo_1556* (230 aa) | |
| | - | *DR_0022* (199 aa)[b] | - | |
| **Bifunctional DNA glycosylases (displaying also a AP lyase activity)** | | | | |
| *fpg* (*mutM*) (269 aa) | *Deide_16240* (276 aa) | *DR_0493* (280 aa) | *Dgeo_0442* (289 aa) | Formamidopyrimidine-DNA glycosylase |
| *nth* (211 aa) | *Deide_01790* (222 aa) | *DR_0289* (*nth1*)(225 aa) | *Dgeo_0248* (233 aa) | Endonuclease III; removes ring-saturated or fragmented pyrimidines |
| | *Deide_23070* (247 aa) | *DR_2438* (*nth2*)(259 aa) | *Dgeo_2290* (242 aa) | |
| | *Deide_12880* (237 aa) | *DR_0928* (338 aa) | *Dgeo_0785* (269 aa) | |
| *nei* (263 aa) | - | - | - | Endonuclease VIII, DNA N-glycosylase/AP lyase |
| **AP endonucleases** | | | | |
| *nfi* (223 aa) | - | *DR_2162* (181 aa) | - | Endonuclease V |
| *nfo* (285 aa) | - | - | - | Endonuclease IV |
| *xthA* (268 aa) | *Deide_03250* (260 aa) | *DR_0354* (283 aa) | *Dgeo_0461* (267 aa) | Exodeoxyribonulease III |
| | *Deide_2p01470* (270 aa) | - | *Dgeo_2484* (254aa) (plasmid) | |

### B. Main DNA repair genes for Nucleotide Excision Repair (NER) [a]

| Gene name | *D. deserti* | *D. radiodurans* | *D. geothermalis* | Protein description |
|---|---|---|---|---|
| *mfd* (1148 aa) | *Deide_06920* (1041 aa) | *DR_1532* (1054 aa) | *Dgeo_0545* (1041 aa) | transcription-repair coupling factor; helicase |
| *uvrA* (940 aa) | *Deide_12760* (1004 aa) | *DR_1771* (1016 aa) | *Dgeo_0694* (1004 aa) | DNA damage recognition protein UvrA; DNA independent ATPase and DNA binding protein |
| | *Deide_2p02060* (831 aa) | *DR_A0188* (922 aa) | - | |
| *uvrB* (673 aa) | *Deide_03120* (671 aa) | *DR_2275* (730 aa) | *Dgeo_1890* (676 aa) | DNA damage binding protein UvrB ; helicase |
| *uvrC* (610 aa) | *Deide_11450* (617 aa) | *DR_1354* (617 aa) | *Dgeo_1124* (616 aa) | Excision nuclease |
| *uvrD* (720 aa) | *Deide_12100* (744 aa) | *DR_1775* (745 aa) | *Dgeo_0868* (741 aa) | DNA helicase II |

| | | | | |
|---|---|---|---|---|
| BS_*ywjD* (320 aa) | *Deide_17800* (301 aa) | *DR_1819* (305 aa) | *Dgeo_1819* (311 aa) | UV DNA damage endonuclease UvsE |
| *yejH* (586 aa) | *Deide_17320* (467 aa) | *DR_A0131* (895 aa) | - | DNA or RNA helicase of superfamily II (COG1061); also predicted nuclease |
| *cho* (295 aa) | - | - | - | UvrC homolog |

## C. Main DNA repair genes for Mismatch Repair [a]

| Gene name | *D. deserti* | *D. radiodurans* | *D. geothermalis* | Protein description |
|---|---|---|---|---|
| *mutL* (615 aa) | *Deide_15600* (550 aa) | *DR_1696* (547 aa) | *Dgeo_1538* (549 aa) | DNA mismatch repair protein; ATPase |
| *mutS* (853 aa) | *Deide_15540* (*mutS1*) (849 aa) | *DR_1039* (*mutS1*) (850 aa) | *Dgeo_1537* (*mutS1*) (880 aa) | DNA mismatch repair protein; ATPase |
| | *Deide_05000* (*mutS2*) (767 aa) | *DR_1976* (*mutS2*) (766 aa) | *Dgeo_0899* (*mutS2*) (789 aa) | MutS2 family protein; ATPase |
| *xseA* (456aa) | *Deide_22980* (402aa) | *DR_0186* (416aa) | *Dgeo_0148* (413aa) | Exonuclease VII, large subunit |
| *xseB* (80 aa) | *Deide_02120* (87 aa)[c] | *DR_2586* (85 aa)[c] | *Dgeo_0027* (83 aa)[c] | Exonuclease VII, small subunit |
| *dam* (278 aa) | - | - | - | Methylase GATC |
| *mutH* (229 aa) | - | - | - | Endonuclease GATC |
| *dcm* (472 aa) | - | - | - | C-5 cytosine methyltransferase |
| *vsr* (156 aa) | - | - | - | Very short patch repair protein; endonuclease |

## D. Main DNA repair genes for Direct Reversal of DNA damage (DR) [a]

| Gene name | *D. deserti* | *D. radiodurans* | *D. geothermalis* | Protein description |
|---|---|---|---|---|
| *ada* (354 aa) | - | - | - | *O*-6-methylguanine-DNA alkyltransferase; Transcription activator |
| *ogt/ybaZ* (171 aa/129 aa) | *Deide_22770* (118 aa) | *DR_0428* (139 aa) | *Dgeo_2101* (124 aa) | *O*-6-alkylguanine transferase |
| *alkB* (216 aa) | - | - | - | N1-methyladenine and N3-methylcytocine repair protein |
| *phrB* (472 aa) | - | - | - | DNA photolyase |
| *BS_splB* (342 aa) | ***Deide_3p02150*** (354 aa) | - | - | DNA repair photolyase |
| *dut* (151 aa) | - | - | - | dUTPase |
| *dcd* (193 aa) | *Deide_16640* (186 aa) | - | *Dgeo_1880* (186 aa) | Deoxycytidine triphosphate deaminase |
| *yggV* (197 aa) | *Deide_19360* (202 aa) | *DR_0179* (200 aa) | *Dgeo_2209* (199 aa) | Xanthosine triphosphate pyrophosphatase |

## E. Main DNA repair genes for recombinational repair (RER) [a]

| Gene name | *D. deserti* | *D. radiodurans* | *D. geothermalis* | Protein description |
|---|---|---|---|---|
| *recA* (353 aa) | ***Deide_19450*** (355 aa)<br>***Deide_1p01260*** (344 aa)<br>***Deide_3p00210*** (344 aa) | *DR_2340* (363 aa) | *Dgeo_2138* (358 aa) | DNA strand exchange and renaturation, DNA-dependent ATPase, DNA- and ATP-dependent coprotease |
| *recB* (1180 aa) | -[d] | - | - | DNA helicase, ATP-dependent dsDNA/ssDNA exonuclease V subunit, ss DNA endonuclease |
| *recC* (1122 aa) | - | - | - | DNA helicase, ATP-dependent dsDNA/ssDNA exonuclease V subunit, ss DNA endonuclease |

| | | | | |
|---|---|---|---|---|
| *recD* (608 aa) | *Deide_16210* (710 aa) | *DR_1902* (715 aa) | *Dgeo_0826* (726 aa) | DNA helicase, ATP-dependent dsDNA/ssDNA exonuclease V subunit, ss DNA endonuclease |
| *recE* (866 aa) | - | - | - | Exonuclease VIII, dsDNA exonuclease, 5'=>3' specific |
| *recF* (357 aa) | *Deide_14250* (363 aa) | *DR_1089* (359 aa) | *Dgeo_1620* (358 aa) | Recombinational repair protein |
| *recG* (693 aa) | *Deide_09960* (780 aa) | *DR_1916* (784 aa) | *Dgeo_1139* (776 aa) | Holliday junction-specific DNA helicase; branch migration inducer |
| *recJ* (577 aa) | *Deide_07130* (714 aa) | *DR_1126* (684 aa) | *Dgeo_1599* (702 aa) | ssDNA exonuclease, 5'=>3' specific |
| *recN* (553 aa) | *Deide_12310* (534 aa) | *DR_1477* (564 aa) | *Dgeo_1194* (555 aa) | Recombination and repair protein |
| *recO* (242 aa) | *Deide_13810* (247 aa) | *DR_0819* (244 aa) | *Dgeo_0855* (243 aa) | Bacterial recombinational repair protein |
| *recQ* (609 aa) *(one HRDC domain)* | *Deide_11320* (731 aa) ***(two HRDC domains)*** | *DR_1289* (824 aa)[e] *(three HRDC domains)* | *Dgeo_1226* (195 aa !) *frameshift? (one HRDC domain) (no RQC domain)* | ATP-dependent DNA helicase RecQ |
| | *Deide_02180* (591 aa) *(one HRDC domain)* | *DR_2444* (603 aa)[e] *(one HRDC domain)* | *Dgeo_0021* (653 aa) *(one HRDC domain)* | Nucleic acid-binding protein, HRDC family |
| | ***Deide_1p01280*** (548 aa) *(no RQC and HRDC domains)* | - | - | ATP-dependent DNA helicase, RecQ family; partial COG0514 |
| | ***Deide_06510_3*** (1683 aa) *(no RQC and HRDC domains)* | - | - | Multidomain protein: 1 is DnaQ, 2 is DinG, 3 is RecQ[f] |
| *recR* (201 aa) | *Deide_06340* (198 aa) | *DR_0198* (220 aa) | *Dgeo_1513* (222 aa) | Recombination and repair protein |
| *recT* (269 aa) | - | - | - | Recombinase, DNA renaturation |
| *recX* (166 aa) | *Deide_12350* (195 aa) | *DR_1310* (204 aa) | *Dgeo_1433* (191 aa) | Regulatory protein |
| *radA* (*sms*) (460 aa) | *Deide_12660* (449 aa) | *DR_1105* (503 aa) | *Dgeo_1212* (449 aa) | DNA repair protein RadA |
| *rusA* (*ybcP*) (120 aa) | - | - | - | Endonuclease/Holliday junction resolvase |
| *ruvA* (203 aa) | *Deide_09360* (202 aa) | *DR_1274* (201 aa) | *Dgeo_0726* (198 aa) | Holliday junction helicase subunit A; branch migration |
| *ruvB* (336 aa) | *Deide_18350* (334 aa) | *DR_0596* (333 aa) | *Dgeo_0404* (331 aa) | Holliday junction helicase subunit B; branch migration |
| *ruvC* (173 aa) | *Deide_20630* (169 aa) | *DR_0440* (179 aa) | *Dgeo_0327* (168 aa) | Holliday junction endonuclease |
| *yqgF* (138 aa) | *Deide_04280* (139 aa) | *DR_2509* (136 aa) | *Dgeo_0425* (232 aa) | Putative Holliday junction resolvase |
| *sbcB* (475 aa) | - | - | - | exodeoxyribonuclease I |
| *sbcC* (1048 aa) | *Deide_16170* (911 aa) | *DR_1922* (909 aa) | *Dgeo_0823* (910 aa) | ATP dependent dsDNA exonuclease |
| *sbcD* (400 aa) | *Deide_16180* (393 aa) | *DR_1921* (416 aa) | *Dgeo_0824* (395 aa) | ATP dependent dsDNA exonuclease |
| *ssb* (178 aa) | *Deide_00120* (297 aa) | *DR_0099* (301 aa) | *Dgeo_0165* (301 aa) (+ *Dgeo_2964* (283 aa), *Dgeo_2969* (352 aa), *Dgeo_3087* (200 aa) on plasmid) | Single-stranded DNA-binding protein |

**F. Other DNA repair related genes** [a]

| Gene name | *D. deserti* | *D. radiodurans* | *D. geothermalis* | Protein description |
|---|---|---|---|---|

| | | | | |
|---|---|---|---|---|
| *mutT* and other Nudix hydrolases | 18 proteins containing Nudix box | 23 proteins containing Nudix box[g] | 14 proteins containing Nudix box | Nudix hydrolases |
| *dnlJ* (671 aa) | ***Deide_12290*** (686 aa)[h]<br>***Deide_1p00290*** (686 aa)[h] | *DR_2069* (700 aa) | *Dgeo_0696* (684 aa) | DNA ligase, NAD-dependent |
| *gyrA* (875 aa) | *Deide_12520* (811 aa) | *DR_1913* (812 aa) | *Dgeo_1016* (809 aa) | DNA gyrase subunit A |
| *gyrB* (804 aa) | *Deide_15490* (674 aa) | *DR_0906* (663 aa) | *Dgeo_0546* (711 aa) | DNA gyrase subunit B |
| *topA* (865 aa) | *Deide_07410* (966 aa) | *DR_1374* (1021 aa) | *Dgeo_2001* (964 aa) | DNA topoisomerase I (Topo IA) |
| - | *Deide_00840* (344 aa) | *DR_0690* (346 aa) | *Dgeo_2058* (349 aa) | DNA topoisomerase IB |
| *radC* (222 aa) | - | - | - | DNA repair protein |
| *xni* (281 aa) | ***Deide_1p00132*** (244 aa) | - | - | 5'-3' exonuclease; exonuclease IX |
| - | - | *DR_1757* (328 aa) | - | nuclease-related domain (NERD) protein |
| *lexA* (202 aa) | *Deide_1p01870* (211 aa)<br>*Deide_01180* (240 aa) | *DR_A0344* (*lexA1*)(210 aa)<br>*DR_A0074* (*lexA2*)(248 aa) | *Dgeo_1366* (236 aa) | Transcriptional regulator, repressor of the SOS regulon, autoprotease |
| *polA* (928 aa) | *Deide_15130* (926 aa) | *DR_1707* (956 aa) | *Dgeo_1666* (907 aa) | DNA polymerase I |
| *polB* (783 aa) | ***Deide_1p00180*** (765 aa) | - | - | DNA polymerase II |
| *dnaE* (1160 aa) | *Deide_21950* (1341 aa) | *DR_0507* (1335 aa) | *Dgeo_0255* (1322 aa) | DNA polymerase III alpha subunit |
| | ***Deide_1p01900*** (1058 aa) | - | - | Putative error-prone DNA polymerase DnaE2 |
| *dnaQ* (243 aa) | *Deide_17790* (202 aa) | *DR_0856* (197 aa) | *Dgeo_1818* (180 aa) | DNA polymerase III epsilon subunit (3'-5' exonuclease subunit) |
| | ***Deide_05970*** (204 aa) | - | - | |
| | ***Deide_06510_1*** (1683 aa) multidomain protein[f]: 1 is DnaQ, 2 is DinG, 3 is RecQ | - | - | |
| *holE* (76 aa) | - | - | - | DNA polymerase III theta subunit |
| *dnaN* (366 aa) | *Deide_00020* (360 aa) | *DR_0001* (393 aa) | *Dgeo_0003* (360 aa)<br>*Dgeo_3063* (367 aa) (plasmid) | DNA polymerase III beta subunit |
| *dnaX* (643 aa) | *Deide_01610* (762 aa) | *DR_2410* (615 aa) | *Dgeo_2135* (737 aa) | DNA polymerase III tau/gamma subunit |
| *holA* (343 aa) | *Deide_10170* (300 aa) | *DR_1244* (300 aa) | *Dgeo_0745* (312 aa) | DNA polymerase III delta subunit |
| *holB* (334 aa) | *Deide_21710* (318 aa) | *DR_2332* (321 aa) | *Dgeo_2262* (316 aa) | DNA polymerase III delta prime subunit |
| *holC* (147 aa) | - | - | - | DNA polymerase III chi subunit |
| *holD* (137 aa) | - | - | - | DNA polymerase III psi subunit |
| BS_*yshC* (570 aa) | *Deide_07030* (568 aa) | *DR_0467* (572 aa) | *Dgeo_1609* (572 aa) | DNA polymerase, family X |
| *umuC* (422 aa) | - | - | - | DNA polymerase type-Y family; error-prone DNA polymerase; in conjunction with UmuD and RecA catalyses translesion DNA synthesis |
| *umuD* (139 aa) | - | - | - | In conjunction with UmuC and RecA facilitates translesion DNA synthesis; autoprotease |
| *dinB* (*dinP*) (351 aa) | - | - | - | Pol IV, DNA polymerase type-Y family, contains 1 UmuC domain |

| | | | | |
|---|---|---|---|---|
| - | ***Deide_1p01880*** (423 aa) | - | - | Related to putative Y-family DNA polymerase |
| *dinG* (716 aa) | ***Deide_06510_2*** (1683 aa) multidomain protein[f]: 1 is DnaQ, 2 is DinG, 3 is RecQ | - | - | Predicted helicase; SOS inducer in *E. coli* |
| - | ***Deide_06520*** (849 aa) | - | - | UvrD/REP helicase; COG0210, UvrD, Superfamily I DNA and RNA helicases |
| BS_*ywqA* (922 aa) | *Deide_08980* (1132 aa) | *DR_1259* (600 aa) + *DR_1258* (916 aa) (2 genes or frameshift?) | *Dgeo_1491* (1126 aa) | DNA helicase SNF2/Rad54 family |
| - | *Deide_17480* (689 aa) | *DR_1572* (frameshift) | - | Superfamily I DNA and RNA helicases (COG3973) |
| *dnaA* (467 aa) | *Deide_00010* (459 aa) | *DR_0002* (466 aa) | *Dgeo_0001* (470 aa) | Chromosomal replication initiator protein |
| *dnaB* (471 aa) | *Deide_04710* (448 aa) | *DR_0549* (448 aa) | *Dgeo_2037* (849 aa; intein-mediated protein splicing) | Replicative DNA helicase |
| *dnaC* (245 aa) | - | - | - | DNA replication protein |
| *dnaG* (581 aa) | *Deide_04900* (572 aa) | *DR_0601* (571 aa) | *Dgeo_1910* (587 aa) | DNA primase |
| *dnaT* (179 aa) | - | - | - | Primosomal protein 1 |
| *priA* (732 aa) | *Deide_00480* (857 aa) | *DR_2606* (925 aa) | *Dgeo_0271* (841 aa) | Primosomal protein N’ |
| *priB* (104 aa) | - | - | - | Primosomal replication protein n |
| *priC* (175 aa) | - | - | - | Primosomal replication protein N’’ |
| *rarA (mgsA, ycaJ)* (447 aa) | *Deide_04980* (451 aa) | *DR_1898* (434 aa) | *Dgeo_1401* (451 aa) | Replication-associated recombination protein |

**G. Other radiation tolerance-associated genes in *D. deserti*, *D. radiodurans* and *D. geothermalis*** [a,i]

| *D. deserti* | *D. radiodurans* | *D. geothermalis* | Protein description |
|---|---|---|---|
| *Deide_03030* (281 aa) | *DR_0167* (328 aa) | *Dgeo_0395* (289 aa) | IrrE, regulator of *recA* expression in *D. radiodurans* |
| *Deide_2p01380* (294 aa) | *DR_A0346* (300 aa) | *Dgeo_2628* (302 aa) (plasmid) | DNA damage repair protein PprA |
| *Deide_09150* (201 aa) | *DR_0423* (208 aa) | *Dgeo_0977* (201 aa) | DNA damage response protein DdrA; Rad52/22 double-strand break repair protein |
| *Deide_02990* (178 aa) | *DR_0070* (199 aa) | *Dgeo_0295* (178 aa) | DdrB |
| *Deide_23280* (232 aa) | *DR_0003* reversed (231 aa) | *Dgeo_0047* reversed (229 aa) | DdrC (DR_0003 and Dgeo_0047 probably incorrectly annotated, see main text) |
| *Deide_01160* (158 aa) | *DR_0326* (198 aa) | *Dgeo_2186* (160 aa) | DdrD |
| *Deide_11220* (228 aa) | *DR_0194* (227 aa) | *Dgeo_1282* (226 aa) | DdrE; Related to zinc metallopeptidase |
| - | *DR_0219* (444 aa) | - | DdrF |
| - | *DR_0227* (242 aa) | - | DdrG |
| *Deide_20641* (82 aa) | *DR_0438* reversed (92 aa) | *Dgeo_0322* (82 aa) | DdrH (DR_0438 probably incorrectly annotated, see main text) |
| *Deide_12530* (205 aa) | *DR_0997* (260 aa) | *Dgeo_1015* (204 aa) | DdrI; Transcriptional regulator, Crp/Fnr family |

| | | | |
|---|---|---|---|
| - | *DR_1263* (222 aa) | - | DdrJ |
| - | *DR_1264* (140 aa) | - | DdrK |
| - | *DR_1439* (175 aa) | - | DdrL |
| *Deide_02930* (180 aa) | *DR_2441* (170aa) | *Dgeo_0078* (168aa) | DdrN |
| ***Deide_20570*** (129 aa)[j]<br>***Deide_3p02170*** (129 aa)[j] | *DR_2574* (131aa) | *Dgeo_0336* (140aa) | DdrO; transcriptional regulator, XRE family |
| - | *DR_B0100* (210 aa) | - | DdrP |
| - | *DR_B0141* (168 aa) | - | HicB-related protein |
| - | *DR_1262* (531 aa) | - | ribonucleoprotein Ro/SS-A-related protein; involved in UV resistance in *D. radiodurans* |
| - | *DR_0171* (230 aa) | - | IrrI |

[a] The gene names are from *E. coli* whenever an *E. coli* ortholog exists, or from *B. subtilis* (with the prefix BS_). The sizes of the predicted gene products (in number of amino acid residues) are indicated. *D. deserti* genes in **blue and bold face** indicate supplementary genes when compared to *D. radiodurans* and *D. geothermalis*. Grey indicates gene products identified in proteome analysis after standard cultivation of *D. deserti*.

[b] DR_0022 did not demonstrate any appreciable uracil-DNA glycosylase activity in a study by Sandigursky et al (1)

[c] DR_2586 contains domain COG1722; this domain is not found in the DR_2586 homologs Deide_02120 and Dgeo_0027

[d] *D. deserti* plasmid P2 contains a "pseudogene" (*Deide_2p00840-Deide_2p00850-Deide_2p00860*, interrupted by 2 stop codons), whose putative product has 27% identity with BS_AddA, and which is probably in operon with *Deide_2p00830* encoding a protein with 25% identity with BS_AddB

[e] In *D. radiodurans*, DR_1289 is required for resistance to DNA damaging agents, whereas DR_2444, which does not contain helicase and RQC domains, is not (2). All three HRDC domains are involved in DR_1289 RecQ function (3, 4)

[f] A homolog of this multidomain protein Deide_06510 is found in *Thermus thermophilus* HB27: TT_P0128 (1649 aa)

[g] Most, if not all, of the *D. radiodurans* Nudix hydrolases are probably not functional homologs of MutT (5)

[h] Deide_12290 and Deide_1p00290 have 57% identity

[i] DdrA, DdrE, DdrI and DdrN homologs with more than 40% identity are present in *T. thermophilus*. Other common genes/proteins are specific to *Deinococcus*.

[j] Deide_20570 and Deide_3p02170 have 84% identity


1. Sandigursky M, Sandigursky S, Sonati P, Daly MJ, Franklin WA (2004) Multiple uracil-DNA glycosylase activities in *Deinococcus radiodurans*. DNA Repair (Amst) 3: 163-169.
2. Huang LF, Zhang SW, Hua XT, Gao GJ, Hua YJ (2006) Construction of the recQ double mutants and analysis of adversity in *Deinococcus radiodurans*. Wei Sheng Wu Xue Bao 46: 205-209.
3. Killoran MP, Keck JL (2006) Three HRDC domains differentially modulate *Deinococcus radiodurans* RecQ DNA helicase biochemical activity. J Biol Chem 281: 12849-12857.
4. Huang L, Hua X, Lu H, Gao G, Tian B, et al. (2007) Three tandem HRDC domains have synergistic effect on the RecQ functions in *Deinococcus radiodurans*. DNA Repair (Amst) 6: 167-176.
5. Xu W, Shen J, Dunn CA, Desai S, Bessman MJ (2001) The Nudix hydrolases of *Deinococcus radiodurans*. Mol Microbiol 39: 286-290.
