# Supplementary material for: Alliance of Proteomics and Genomics to Unravel the Specificities of Sahara Bacterium Deinococcus deserti
Source: PLoS Genet. 2009 Mar 27;5(3):e1000434. doi: 10.1371/journal.pgen.1000434 (PMC2669436; doi:10.1371/journal.pgen.1000434)
Supplement: Table S11 — Radiation/desiccation response motif identified in D. deserti. (0.11 MB PDF) [file pgen.1000434.s016.pdf]

**Table S11. Radiation/desiccation response motif identified in *D. deserti***			
**gene**[a]	**position**	**site**	**score**[b]
Deide_23280 (correct *ddrC*)	-37	TTATGTTTTTAGCATAA	15.00
Deide_02990 (*ddrB*)	-30	TTATGTTATTTACATAA	14.86
Deide_01160 (*ddrD*)	-27	TTCTGCTTTAAACATAA	14.56
Deide_15490 (*gyrB*)	-145	TTACGTTATATACGTAA	14.21
Deide_18730 (operon, conserved genes: *Deide_18730*, *Deide_18720*, *Deide_18710*, *Deide_18700*, *Deide_18690*)	-43	TTACGTTCAAAGCGTAA	14.20
Deide_12520 (*gyrA*)	-119	TTACGCCCATAACATAA	14.11
Deide_3p00210 (*recA-P3*)	-64	TTATGCTGGAATCATAA	14.11
Deide_1p01260 (*recA-P1*)	-64	TTATGCTGCAATCATAA	14.06
Deide_19430 (*cinA*; operon with *ligT* + *recA*)	-2	TTATGCTTCTAGCAGAA	13.98
Deide_11320 (*recQ*)	-35	TTACGCTAATGACATAA	13.96
Deide_2p01380 (*pprA*)	-91	TTCTGTTTTATACAGAA	13.94
Deide_12100 (*uvrD*)	-39	TTACGCTTTCAACAGAA	13.84
Deide_09150 (*ddrA*)	-42<br>-20	TTACGCCTTGACCAGAA<br>TTCTGTTATAAACTAAA	13.40<br>13.79
Deide_00600 (transketolase)	-191	TTCTGTTCTCACCGGAA	13.79
Deide_12760 (*uvrA*)	-67	TTACGCACGCAACGTAA	13.74
Deide_16210 (*recD*)	—45	TTCTGCCACAAACAGAA	13.65
Deide_16610 (membrane protein)	+15	TTCTGTCAGAAGCGGAA	13.57
Deide_03120 (*uvrB*)	-92	TTACGTCAGGGGCGTAA	13.53
Deide_00120 (*ssb*)	-132	TTACGGCATTGACGTAA	13.52
Deide_04721 (conserved protein)	-18	TTCTGCTAGTATCAGAA	13.52
Deide_08700 (conserved protein)	-395<br>-159	TTACGGCGGGATCATAA<br>TTATGATGAGTTCATAA	13.34<br>12.91
Deide_20312 (glycosyl transferase)	-66	TTTTGTTGCCATCGGAA	13.31
Deide_07131 (membrane protein; operon with *recJ*)	-32	TTTTGCTCTGCACGAAA	12.96
Deide_22810 (*Deinococcus*-specific)	-67	TTTCGCTTACAACGAAA	12.86
Deide_20570 (*ddrO*)	-173	TTCTGTATTGACCGTAC	12.84

[a] Grey indicates gene products identified in proteome analysis after standard cultivation.

[b] The motifs were identified using Virtual Footprint (1). First, a position weight matrix was created using the palindromic sequences near genes from *D. radiodurans* and *D. geothermalis*, listed in Makarova et al (2), to search for matches near homologous genes in *D. deserti*. Then the identified sequences were added to those from *D. radiodurans* and *D. geothermalis* to create a new position weight matrix and to scan the entire *D. deserti* genome.

1. Münch R, Hiller K, Grote A, Scheer M, Klein J, et al. (2005) Virtual Footprint and PRODORIC: an integrative framework for regulon prediction in prokaryotes. Bioinformatics 21: 4187-4189.

2. Makarova KS, Omelchenko MV, Gaidamakova EK, Matrosova VY, Vasilenko A, et al. (2007) *Deinococcus geothermalis*: the pool of extreme radiation resistance genes shrinks. PLoS ONE 2: e955.
