# Supplementary material for: Alliance of Proteomics and Genomics to Unravel the Specificities of Sahara Bacterium Deinococcus deserti
Source: PLoS Genet. 2009 Mar 27;5(3):e1000434. doi: 10.1371/journal.pgen.1000434 (PMC2669436; doi:10.1371/journal.pgen.1000434)
Supplement: Table S12 — Primers for RT-PCR. (0.06 MB PDF) [file pgen.1000434.s017.pdf]

**Table S12. Primers for RT-PCR**		
**Gene**	**Primer**	**Primer sequence**
Deide_06120 (*tufA*)	Dd06120FW	TTTGAGCGCACGAAGCCCCA
	Dd06120RV	TGACGGGTCGGCGTGTTGTA
Deide_19450 (*recA-C*)	recAC-FW2	AGCTACGGTGACGAGCGCAT
	recAC-DN	AGCCAGGGTAAGGAAGACTGGA
Deide_1p01260 (*recA-P1*)	recAP-FW2	AGCTACGGGGACGAACGCAT
	recAP1-DN	GGGCAGCGGGATTGATATTAAGG
Deide_3p00210 (*recA-P3*)	recAP-FW2	AGCTACGGGGACGAACGCAT
	recAP3-DN	GCCATACCAGTGACCCCGGA
Deide_1p00180 (*polB*)	DdP100180FW2	ACCTGCCGTTTCTGCATTAC
	DdP100180RV2	CCAAATAGACCCGTCCTTCA
Deide_1p01880 (Y polymerase)	DdP101880FW2	TGCGTGCCAAAGCGGGAGAA
	DdP101880RV2	TCGGTGCGGTGCCCTTTCAA
Deide_1p01900 (*dnaE2*)	DdP101900FW	TTGCGGTCCATTACGCCGGA
	DdP101900RV	AAATGCGTGGGCGTGCGACT
Deide_3p02150 (photolyase-like)	DdP302150FW	TTTTCCTGACGCCCAGCGCA
	DdP302150RV	AGGACAGCGCGCACCCATTT
Deide_23280 (*ddrC*)	Dd23280FW	TCAGCTGGGCAGCATCAGCA
	Dd23280RV	TGCGCGCCGTGTTCCTGAAT
